# Synthesis and antimitotic activity of 2-phenyl-6-pyridinyl-2*H*-pyrazolo[4,3-*c*]pyridines

**DOI:** 10.1039/d5ra09208f

**Published:** 2026-05-12

**Authors:** Vaida Aleksienė, Eva Řezníčková, Aurimas Bieliauskas, Veronika Vojáčková, Veronika Molitorová, Austėja Šalvytė-Nikliauzienė, Sergey Belyakov, Asta Žukauskaitė, Eglė Arbačiauskienė, Vladimír Kryštof, Algirdas Šačkus

**Affiliations:** a Institute of Synthetic Chemistry, Faculty of Chemical Technology, Kaunas University of Technology K. Baršausko g. 59 LT-51423 Kaunas Lithuania algirdas.sackus@ktu.lt; b Department of Experimental Biology, Faculty of Science, Palacký University Šlechtitelů 27 CZ-77900 Olomouc Czech Republic vladimir.krystof@upol.cz; c Department of Organic Chemistry, Faculty of Chemical Technology, Kaunas University of Technology Radvilėnų pl. 19 LT-50254 Kaunas Lithuania; d Latvian Institute of Organic Synthesis Aizkraukles 21 LV-1006 Riga Latvia; e Department of Chemical Biology, Faculty of Science, Palacký University Šlechtitelů 27 CZ-77900 Olomouc Czech Republic

## Abstract

An efficient synthetic route to 2-phenyl-6-pyridinyl-2*H*-pyrazolo[4,3-*c*]pyridines, alongside comprehensive structural elucidation and biological evaluation, is reported. Among the newly synthesized compounds, 7f, which contains 4-fluorophenyl and pyridin-2-yl substituents at the 2- and 6-positions, respectively, exhibited the strongest submicromolar cytotoxicity across various cancer cell lines. This compound compromised microtubule integrity, induced mitotic defects and aberrant cytokinesis, triggered endoreduplication, and ultimately resulted in cell death. Our findings highlight the potential of these pyrazolo[4,3-*c*]pyridine derivatives as antimitotic agents, providing a basis for further development of anticancer therapeutics.

## Abstract 2

A series of new 2-phenyl-6-pyridinyl-2*H*-pyrazolo[4,3-*c*]pyridines was prepared. The most active compound 7f disrupts the microtubule cytoskeleton, alters cell morphology, causes mitotic defects, and ultimately leads to cell death.

## Graphical abstract

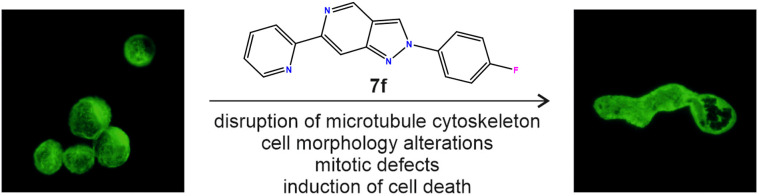

## Introduction

Pyrazoles are a class of important nitrogen-containing heterocyclic compounds that cover a range of natural products and synthetic derivatives.^1–4^ Due to excellent pharmacological properties, the pyrazole moiety is frequently used in drug discovery,^5^ resulting in numerous approved pharmaceuticals and veterinary drugs.^3,6^ For instance, sildenafil is used to treat erectile dysfunction^7^ and pulmonary arterial hypertension,^8^ while celecoxib, tepoxalin, epirizole, and lonazolac are non-steroidal anti-inflammatory drugs.^9–11^

**Fig. 1:**
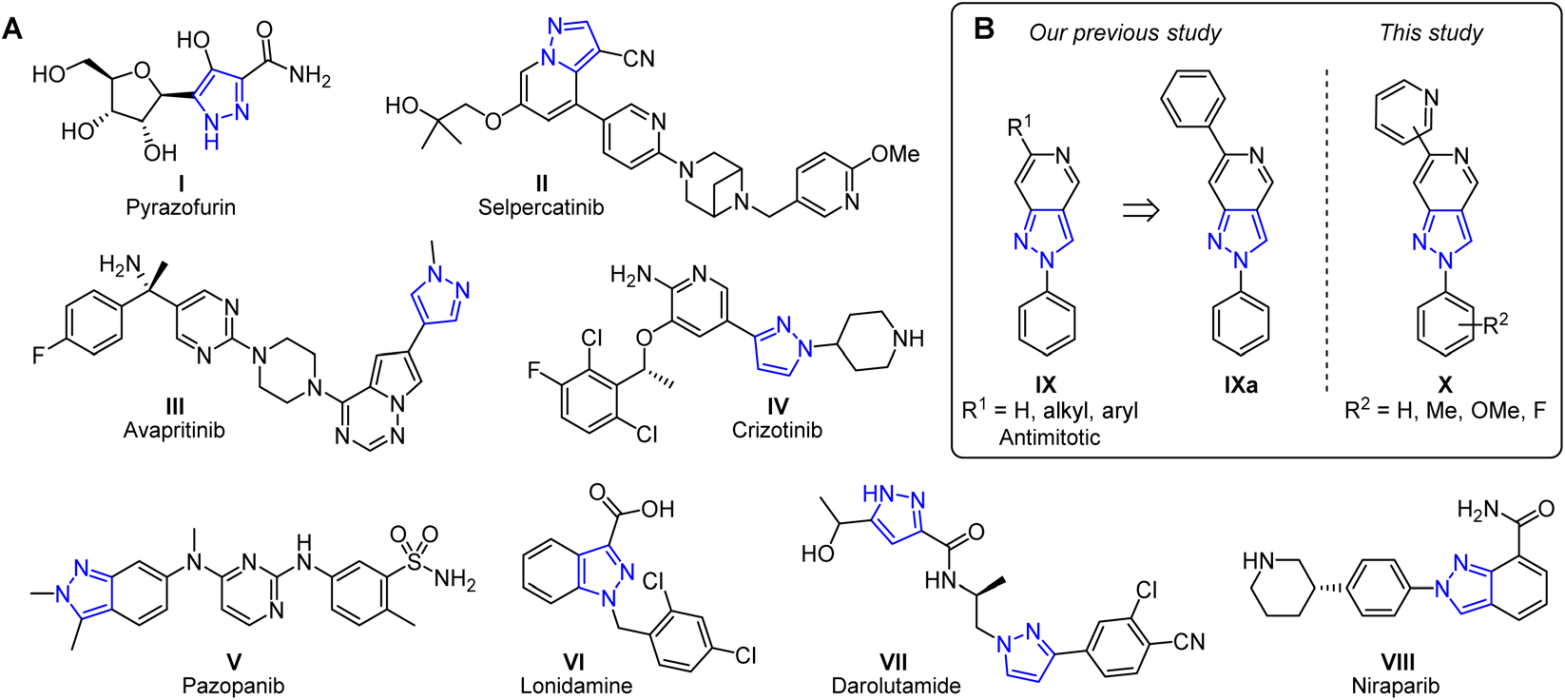
Pyrazole moiety-containing anticancer agents (A) and the structural framework of our previous study that inspired the present work (B).

Pyrazole-based derivatives are also widely investigated for their anticancer and antitumor activities.^12,13^ Among natural products, pyrazofurin (I) (Fig. 1A), a C-nucleoside analog produced by *Streptomyces candidus* and other actinobacteria, exhibits antitumor properties, though a phase I clinical trial primarily revealed toxicity without therapeutic effect.^14–16^ Synthetic pyrazole drugs include several clinically approved kinase inhibitors. For instance, tyrosine kinase inhibitors selpercatinib (II), avapritinib (III), and crizotinib (IV) target the RET, KIT/PDGFRA, and ALK/ROS1 pathways, respectively, and are used to treat thyroid, multidrug-resistant gastrointestinal stromal tumors, and non-small-cell lung cancers.^17–20^ Indazole moiety-containing pazopanib (V) acts as a multi-targeted tyrosine kinase inhibitor and is approved for the treatment of renal cell carcinoma.^21^ Beyond kinase inhibition, pyrazole drugs also work *via* other mechanisms. For instance, lonidamine (VI) selectively inhibits aerobic glycolysis and energy metabolism in tumor cells and is capable of sensitizing tumors to chemo-, radio-, and photodynamic-therapy and hyperthermia,^22–24^ while darolutamide (VII) is a nonsteroidal androgen receptor antagonist used to treat non-metastatic castration-resistant prostate cancer.^25^ Finally, the poly(ADP-ribose) polymerase (PARP) inhibitor niraparib (VII) represses DNA damage repair in cancer cells and is used for the maintenance treatment of epithelial ovarian, fallopian tube, and primary peritoneal cancer.^26^

Although anticancer pyrazoles exhibit a wide range of structural diversity, recent research increasingly focuses on condensed systems that combine the pharmacological properties of the pyrazole ring with other heterocyclic frameworks.^27,28^ Among condensed pyrazole derivatives, pyrazolopyridines, which can exist in either the more prevalent 1*H*- or synthetically more demanding 2*H*-tautomeric form, are renowned for their anticancer properties.^29^ 1*H*-Pyrazolo[3,4-*b*]pyridines, in particular, are reported as potent inhibitors of various kinases^30^ and antileukemic agents.^31^ Although less prevalent in the scientific literature, the bioactivities of other pyrazolopyridines are also notable. 1*H*-Pyrazolo[3,4-*c*]pyridines were identified as antiproliferative pro-apoptotic agents^32,33^ and kinase inhibitors.^34^ 1*H*-Pyrazolo[4,3-*b*]pyridines were evaluated as selective c-Met^35^ or dual FLT3/CDK4 inhibitors.^36^ In the case of 2*H*-pyrazolo[4,3-*c*]pyridines, inhibitory activity against p38α, aurora A, CK1δ^37^ and p90 ribosomal S6 kinases 2 (RSK2)^38^ was reported, while in our recent works, we also studied antiproliferative,^39^ photodynamic anticancer^40^ and antimitotic^41^ properties of their derivatives. Our previous study investigated the structure–activity relationships of 6-alkyl- and 6-aryl-2-phenyl-2*H*-pyrazolo[4,3-*c*]pyridines IX (Fig. 1B). Several of them showed promising anticancer activity *in vitro*, including cell cycle arrest in mitosis and induction of apoptosis.^41^ One of the most potent compounds, 2,6-diphenyl-2*H*-pyrazolo[4,3-*c*]pyridine IXa, exhibited *in vitro* cytotoxicity in the K562 cell line with a micromolar GI_50_ value of 3.4 µM. Building on these results, in the current work, we explore the synthesis and antimitotic activity of new 2*H*-pyrazolo[4,3-*c*]pyridines X bearing pyridinyl substituents at the 6-position and variously substituted phenyl groups at the 2-position, as structural modifications to enhance the properties of the compounds.^42,43^

## Results and discussion

### Synthesis

**Scheme 1:**
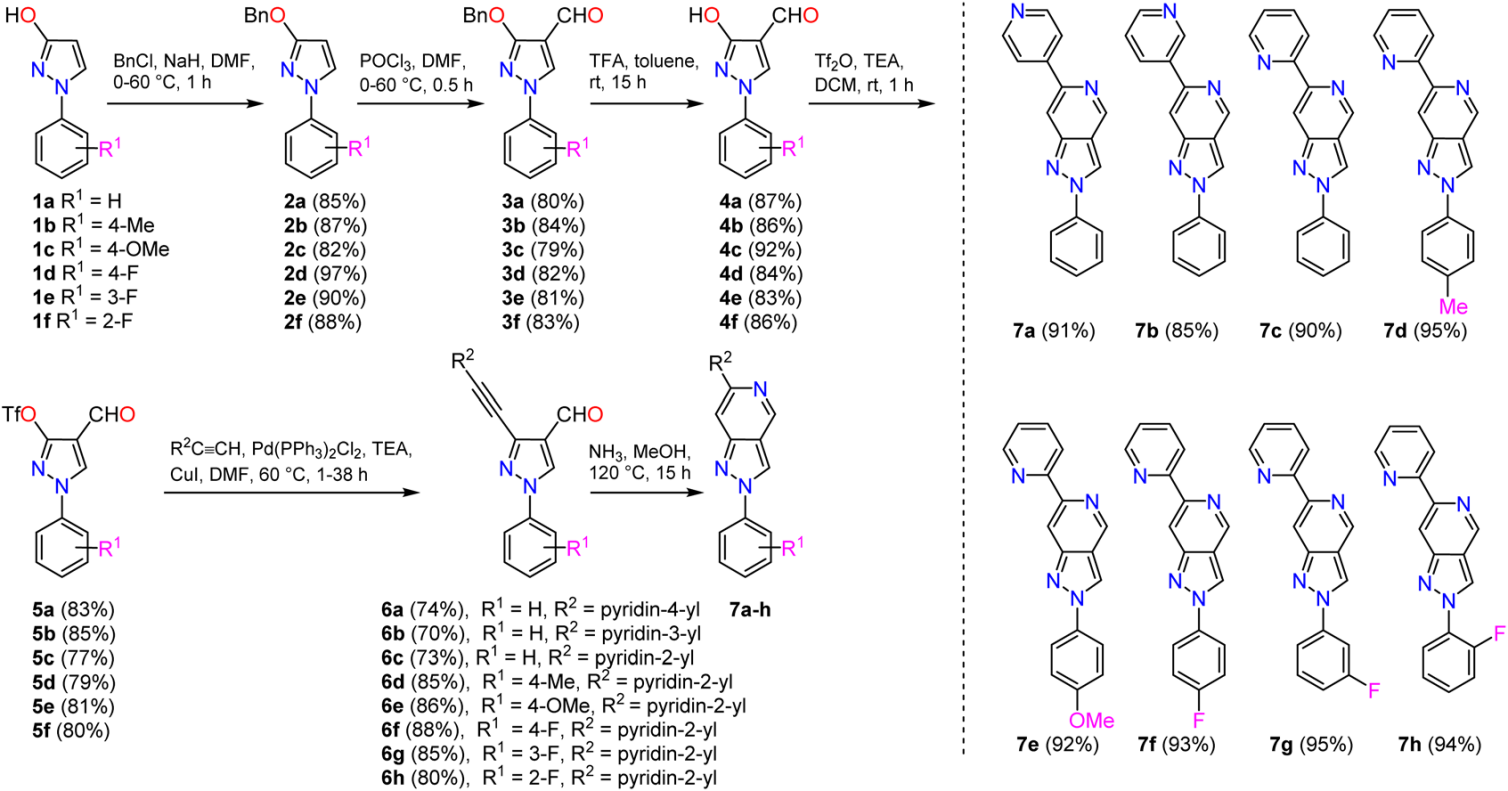
Synthesis of 2-phenyl-6-pyridinyl-2*H*-pyrazolo[4,3-*c*]pyridines 7a–h.

For the synthesis of 2-phenyl-6-pyridinyl-2*H*-pyrazolo[4,3-*c*]pyridines 7a–h (Scheme 1), the starting 1-phenyl-1*H*-pyrazol-3-ols 1a–f were prepared *via* addition–cyclisation reaction of commercially available variously 2-, 3- and 4-substituted phenyl hydrazine hydrochlorides and ethyl acrylate in the presence of a base and a subsequent oxidation of the obtained pyrazolidin-3-ones by FeCl_3_ as previously described.^44–47^ 1-Phenyl-1*H*-pyrazol-3-ols 1a–f were deprotonated by NaH and subjected to *O*-benzylation by benzyl chloride to provide 3-*O*-protected pyrazoles 2a–f in very good yields (82–97%). The compounds were further formylated under Vilsmeier–Haack reaction conditions to yield pyrazole-4-carbaldehydes 3a–f in good yields (79–84%). For the *O*-deprotection, trifluoroacetic acid was employed, and corresponding 3-hydroxy-1*H*-pyrazole-4-carbaldehydes 4a–f were obtained in very good yields (83–92%). The latter compounds underwent the triflation reaction smoothly with triflic anhydride in the presence of TEA, stirring the reaction mixture in DCM at room temperature. Obtained pseudohalides 5a–f were coupled with pyridin-2-yl, pyridin-3-yl, or pyridin-4-yl acetylenes under the standard sonogashira cross-coupling reaction conditions (Pd(PPh_3_)_2_Cl_2_, CuI, TEA in DMF) to yield appropriate 3-alkynylpyrazoles 6a–h in yields of 70–88%. We have demonstrated previously that 3-alkynyl-1*H*-pyrazole-4-carbaldehydes can serve as excellent starting materials for the synthesis of various pyrazole-containing fused heterocyclic systems, including but not limited to pyrazolo[4,3-*c*]pyridines, pyrazolo[4,3-*f*][1,2,3]triazolo[5,1-*c*][1,4]oxazepines, pyrazolo[4′,3′:3,4]pyrido[1,2-*a*]benzimidazoles, and related compounds.^48–50^ In this work, compounds 6a–h were subjected to dry ammonia-induced cyclisation, and 2-phenyl-6-pyridinyl-2*H*-pyrazolo[4,3-*c*]pyridines 7a–h were formed in excellent yields (85–95%).

### NMR spectroscopic investigations

**Fig. 2:**
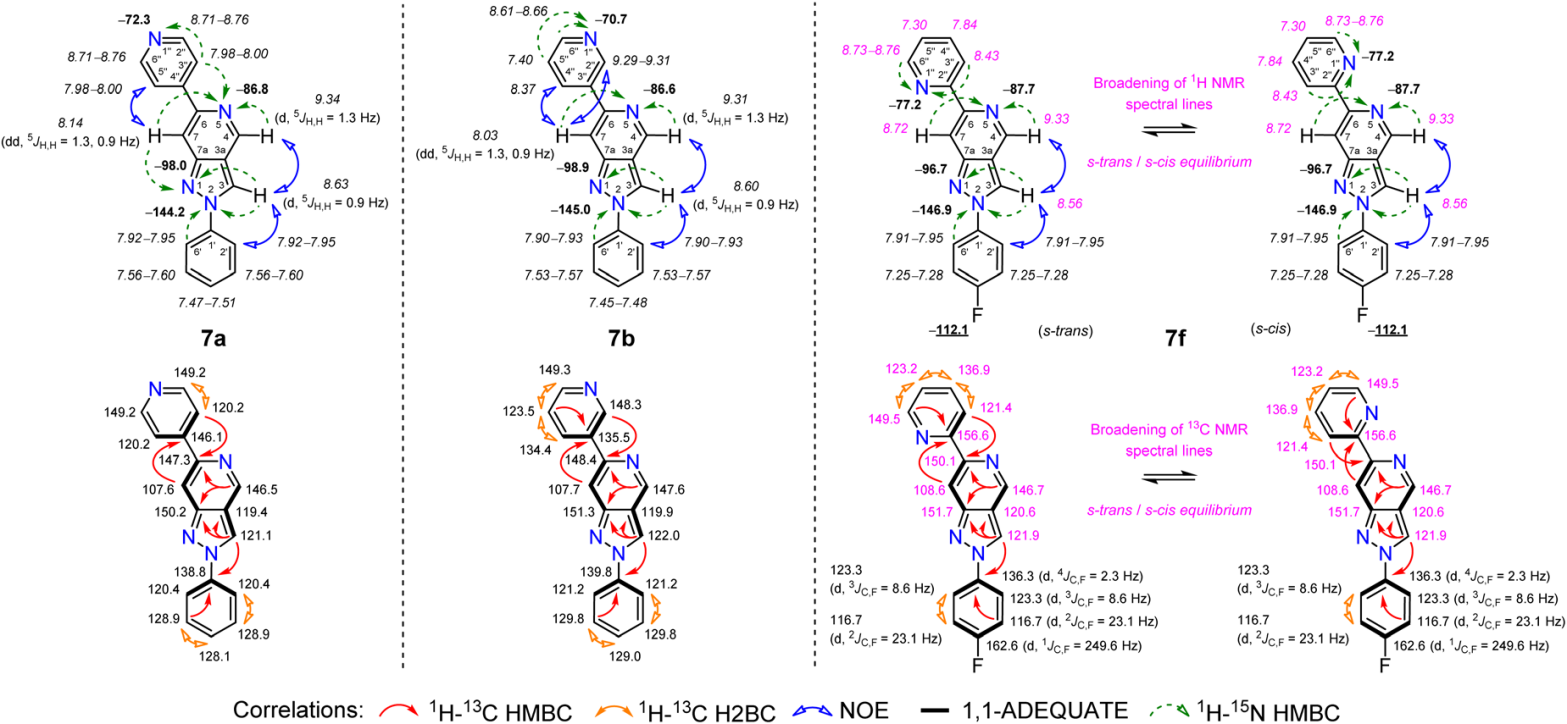
Relevant ^1^H–^13^C HMBC, ^1^H–^13^C H2BC, ^1^H–^15^N HMBC, 1,1-ADEQUATE, and ^1^H–^1^H NOESY correlations, as well as ^1^H NMR (italic), ^13^C NMR, ^15^N NMR (bold), and ^19^F NMR (bold, underlined) chemical shifts of compounds 7a, 7b, and 7f (CDCl_3_).

The structures of all targeted 2-phenyl-6-pyridinyl-2*H*-pyrazolo[4,3-*c*]pyridines 7a–h were unambiguously confirmed using various spectroscopic methods, including multinuclear NMR spectroscopy, infrared spectroscopy (IR), and high-resolution mass spectrometry (HRMS). The main information for structure elucidation was obtained through a combination of standard and advanced NMR techniques, including ^1^H–^13^C HMBC, ^1^H–^13^C HSQC, ^1^H–^13^C H2BC, ^1^H–^15^N HMBC, ^1^H–^1^H COSY, ^1^H–^1^H TOCSY, ^1^H–^1^H NOESY, 1D selective NOESY, and 1,1-ADEQUATE experiments, which provided an unambiguous assignment of the signals. The NMR data for all studied compounds are provided in the Experimental section and SI; moreover, an in-depth data analysis showed that the chemical shift values were highly consistent across the 2-phenyl-6-pyridinyl-2*H*-pyrazolo[4,3-*c*]pyridine series, confirming the shifts for each position. The NMR data for the representative compounds 7a, 7b, and 7f are shown in Fig. 2.

Key information about the formation of the pyrazolo[4,3-*c*]pyridine ring system was easily obtained through various one- and two-dimensional ^1^H and ^15^N NMR techniques. This was mainly due to three clearly distinguishable methine protons, which are separated from other aromatic protons and are, most importantly, next to two “pyridine-like” and one “pyrrole-like” nitrogen atoms. In all targeted compounds 7a–h, these methine protons did not show any ^1^H–^1^H COSY correlations but showed clear ^1^H–^1^H TOCSY cross-peaks in the spectral data, enabling the identification of two distinct spin systems within the pyrazolo[4,3-*c*]pyridine moiety. Specifically, in the ^1^H–^1^H TOCSY spectra, the most downfield methine proton 4-H, resonating from *δ* 9.25 to 9.34 ppm in compounds 7a–h, shared a TOCSY cross-peak with the methine proton 7-H. Then, 7-H shared a TOCSY cross-peak with the methine proton 3-H. This was supported by the ^1^H NMR spectra of compounds 7a and 7b, where long-range ^5^*J* couplings were clearly seen.

The methine protons 3-H (d, ^5^*J*_H,H_ = 0.9 Hz) and 4-H (d, ^5^*J*_H,H_ = 1.3 Hz) appeared as doublets, while the proton 7-H (dd, ^5^*J*_H,H_ = 1.3, 0.9 Hz) appeared as a doublet of doublets. In compounds 7c–h, a noticeable broadening of the ^1^H NMR spectral lines was seen throughout the 6-pyridinyl-2*H*-pyrazolo[4,3-*c*]pyridine ring system.

Next, the NOESY spectral data provided additional insights into connectivity based on through-space correlations in compounds 7a–h. For instance, a clear NOE was observed between the most downfield 4-H proton and the nearby 3-H proton, followed by an NOE correlation between the 3-H and the nearby phenyl group protons, confirming their close proximity in space. Meanwhile, the methine proton 7-H showed distinct NOEs only in compounds 7a and 7b with the nearby protons from the pyridin-4-yl (*δ* 7.98–8.00 ppm) and pyridin-3-yl (*δ* 8.37 and 9.29–9.31 ppm) groups attached at C-6 of the pyrazolo[4,3-*c*]pyridine ring system. Interestingly, for compounds 7c–h with a pyridin-2-yl group at site 6, NOE correlations were also expected but not observed with the methine proton 7-H.

Furthermore, after successfully identifying the methine protons of the pyrazolo[4,3-*c*]pyridine ring system, the ^15^N NMR spectral analysis of compounds 7a–h was straightforward. The ^1^H–^15^N HMBC spectra showed that the proton 3-H had long-range correlations with neighboring N-2 “pyrrole-like” (from *δ* −144.2 to −148.3 ppm, except for compound 7h where it was at *δ* −157.4 ppm due to the 2-fluorophenyl group) and N-1 “pyridine-like” (from *δ* −95.3 to −98.9 ppm) nitrogen atoms, while the “pyridine-like” N-5 (from *δ* −86.6 to −88.5 ppm) nitrogen atom correlated with the nearby H-4 and H-7 protons. The most downfield ^15^N resonances appeared in the 6-pyridinyl groups, ranging from *δ* −70.7 to −77.4 ppm.

Finally, assigning ^13^C resonances for the rest of the pyrazolo[4,3-*c*]pyridine ring system was straightforward using a combination of ^1^H–^13^C HSQC and ^1^H–^13^C HMBC techniques. For the representative compounds 7a, 7b, and 7f, a 1,1-ADEQUATE experiment was also performed. Specifically, for compound 7a, the multiplicity-edited ^1^H–^13^C HSQC spectrum showed that the distinct methine protons 3-H, 4-H, and 7-H each connect by one bond to carbons C-3 (*δ* 121.1 ppm), C-4 (*δ* 146.5 ppm), and C-7 (*δ* 107.6 ppm), respectively. This finding, along with data from the 1,1-ADEQUATE and ^1^H–^13^C HMBC experiments, helped us to unambiguously assign the signals of the quaternary carbons C-3a (*δ* 119.4 ppm), C-6 (*δ* 147.3 ppm), and C-7a (*δ* 150.2 ppm). In all the compounds analyzed, a quaternary carbon C-7a was a bit more downfield than C-6, while C-3a was the most upfield. For compounds 7c–h containing a pyridin-2-yl group at site 6, a significant broadening of the ^13^C NMR spectral lines was also observed, similar to what was seen in the ^1^H NMR spectra. However, an in-depth 2D NMR analysis allowed us to identify the derivatives obtained. In the case of a representative compound 7f, the long-range ^1^H–^13^C HMBC spectral data clearly confirmed the connectivity between the quaternary carbon C-6 (*δ* 150.1 ppm) in the pyrazolo[4,3-*c*]pyridine ring system and the methine proton 3-H″ (*δ* 8.43 ppm) from the neighboring pyridin-2-yl group. Furthermore, this was supported by the 1,1-ADEQUATE and ^1^H–^13^C HSQC experiments, which clearly showed that the methine carbon C-3″ (*δ* 121.4 ppm) was adjacent to the quaternary carbon C-2″ (*δ* 156.6 ppm), consistent with data reported for compounds containing the pyridin-2-yl moiety.^51^

Derivatives 7c–h containing a pyridin-2-yl group at site 6 can be described as “2,2′-bipyridine”-like compounds, existing in an equilibrium between s*-trans* and s*-cis* conformers in solution.^52,53^ Many “2,2′-bipyridine”-like compounds related to metal complexes favor the s*-cis* conformer, but it is well known that the main form of unchelated “2,2′-bipyridine”-like compounds is the s*-trans* conformer.^51^ Some biheterocycles, including 2,2′-bipyridine derivatives, can form weak intramolecular hydrogen bonds between their heterocyclic fragments, which leads to a characteristic downfield shift in the ^1^H NMR spectrum.^54,55^ However, intramolecular hydrogen bonds are very weak; thus, they are insufficient to stabilize the corresponding conformations. Comparing the ^1^H NMR spectral data for all pyridin-2-yl derivatives 7c–h with those of pyridin-4-yl 7a and pyridin-3-yl 7b derivatives, substituted at site 6, clearly shows more key differences. In addition to the significant broadening of NMR lines in the 7c–h series, the distinctive methine proton 7-H (*δ* 8.64–8.75 ppm) is noticeably downfield by 0.5–0.7 ppm compared to derivatives 7a and 7b, suggesting possible intramolecular hydrogen bonding. Overall, the absence of NOE correlations, along with other key differences in the ^1^H NMR spectral data between the pyridin-2-yl derivatives 7c–h and derivatives 7a and 7b, is mainly attributed to the equilibrium between s*-trans* and s*-cis* conformers, with the s*-trans* form being predominant, as reported in the literature.

### Single-crystal X-ray diffraction analysis

**Fig. 3:**
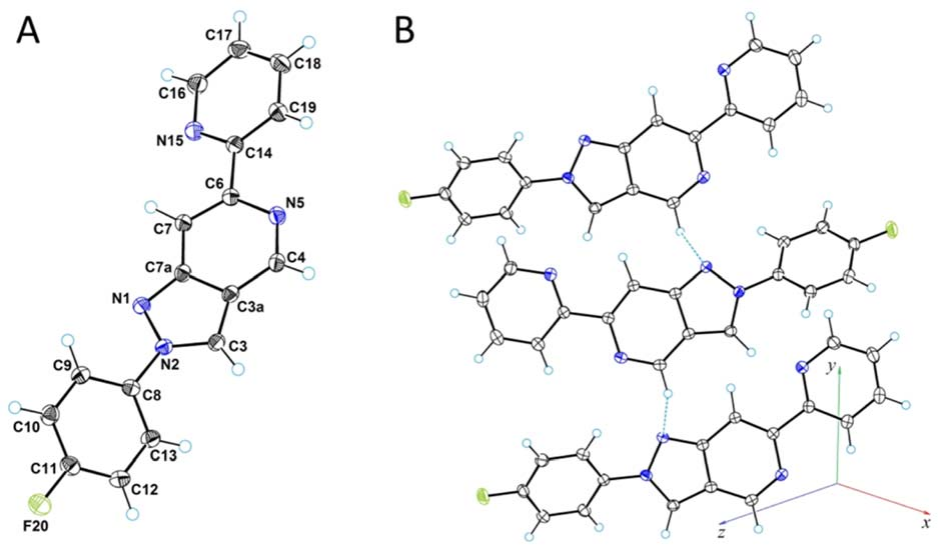
ORTEP diagram for compound 7f (A) and a fragment of its molecular packing in the crystal structure showing CH⋯N hydrogen bonds (B).

To objectively establish the structure of the prepared derivatives, a single-crystal X-ray diffraction analysis of the most active compound 7f was carried out. Fig. 3A illustrates a perspective view of the molecule with thermal ellipsoids and the atom-numbering scheme followed in the text. In the molecular structure, the 4-fluorophenyl substituent forms a dihedral angle of 36.5(2)° with the plane of the pyrazolo[4,3-*c*]pyridine system. The 2-pyridyl substituent forms an even smaller dihedral angle of 16.0(2)° with this system. Low dihedral angle values promote conjugation throughout the molecule.

The C4 carbon atom has increased electronegativity; this makes the C4–H group capable of forming CH⋯ N-type hydrogen bonds. In the crystal structure, a moderate intermolecular C4–H⋯N1 hydrogen bond with a length of 3.322(2) Å (H⋯*N* = 2.75(2) Å, C–H⋯O = 117(3)°) was detected. These interactions lead to the formation of molecular chains along the crystallographic direction [010] in the crystal structure. Fig. 3B shows a molecular chain formed by CH⋯N hydrogen bonds. The crystal structure is chiral (space group is *P*2_1_2_1_2_1_) despite the absence of asymmetric atoms.

### Biology

Prepared derivatives 7a–h were evaluated for their cytotoxicity against three human cancer cell lines: K562 (chronic myeloid leukemia), MV4-11 (acute myeloid leukemia), and CEM (acute lymphoblastic leukemia). The compound IXa from the previous series, along with two standards (taxol and vinflunine), were included for comparison purposes. The majority of the compounds exhibited good cytotoxicity, with GI_50_ values in the low micromolar or submicromolar range (Table 1).

**Table 1:** *In vitro* cytotoxicity of 2-phenyl-6-pyridinyl-2*H*-pyrazolo[4,3-*c*]pyridines 7a–h

Cmpd.	GI_50_ (µM)		
	K562	MV4-11	CEM
7a	23.07 ± 2.73	>25	>25
7b	8.93 ± 3.52	17.94 ± 2.46	18.57 ± 1.03
7c	0.61 ± 0.23	0.55 ± 0.32	0.39 ± 0.08
7d	1.76 ± 0.38	2.18 ± 0.55	6.28 ± 2.47
7e	19.41 ± 4.36	20.47 ± 5.24	22.77 ± 3.16
7f	0.03 ± 0.02	0.05 ± 0.00	0.06 ± 0.01
7g	0.04 ± 0.00	0.15 ± 0.03	0.18 ± 0.07
7h	0.65 ± 0.20	0.44 ± 0.08	0.38 ± 0.06
IXa	3.83 ± 0.86	3.86 ± 0.96	7.24 ± 3.28
Taxol	0.02 ± 0.00	0.01 ± 0.00	0.02 ± 0.01
Vinflunine	0.22 ± 0.02	0.10 ± 0.01	0.23 ± 0.12

The cytotoxicity of the compounds was strongly influenced by the position of the nitrogen atom in the pyridine substituent. Specifically, among unsubstituted 2-phenyl derivatives 7a–c, the pyridin-2-yl derivative 7c exhibited significantly higher cytotoxicity, whereas pyridin-3-yl and pyridin-4-yl derivatives 7b and 7a only possessed moderate and low cytotoxicity, respectively. The enhanced activity of pyridin-2-yl derivatives (*e.g.*, 7c, 7f, 7g) compared to their pyridin-3-yl (7b) and pyridin-4-yl (7a) analogues may be attributed to the ability of the 2-pyridyl moiety to adopt conformations enabling more favourable intramolecular interactions and potentially improved binding to the biological target.

Compound 7c was therefore chosen for further optimization of its biological activity. Introduction of substituents on the phenyl ring at the 2-position revealed a clear substituent effect. Addition of electron-donating groups such as methyl and methoxy (7d, 7e) diminished the activity relative to the parent compound 7c, whereas incorporation of fluorine resulted in enhanced cytotoxicity. Specifically, introduction of fluorine at the *ortho*-position on the 2-phenyl ring did not affect the cytotoxicity of the compound, while substitution with both *meta*- and especially *para*-fluorine resulted in the most active compounds of the series, 7g and 7f, respectively. The increased activity of fluorinated derivatives can be explained by a combination of electronic and physicochemical effects. In particular, the electron-withdrawing nature of fluorine, together with increased lipophilicity, may enhance cell membrane permeability and improve interactions with the cellular target, for example, by modulating π–π stacking or hydrogen bonding interactions.

**Fig. 4:**
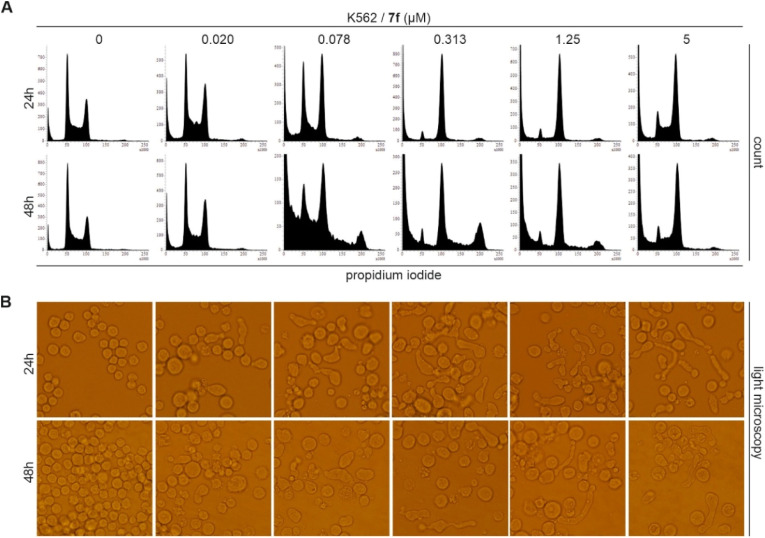
Cell cycle analysis (A) and microscopic observations (B) of K562 cells treated with compound 7f for 24 and 48 h.

Subsequently, compound 7f, which displayed the strongest antiproliferative activity in the panel of selected cancer cell lines, was selected for further evaluation of its biological effects *in vitro*. The potential of 7f was further confirmed by evaluating its effects on two non-cancer cell lines, MRC-5 and BJ fibroblasts. Viability of confluent cultures of these cells, mimicking the state of nonproliferating healthy cells, was not significantly affected by 7f up to the concentration of 10 µM. Initial flow cytometry analysis of K562 leukemic cells treated with increasing concentrations of 7f showed a marked enrichment of G2/M cell population after 24 h treatment (Fig. 4A). Moreover, parallel microscopic observations of 7f-treated cells (Fig. 4B) revealed significant morphological alterations characterized by a substantial number of strikingly elongated cells together with a concentration- and time-dependent increase in the population of cells showing signs of cell death.

**Fig. 5:**
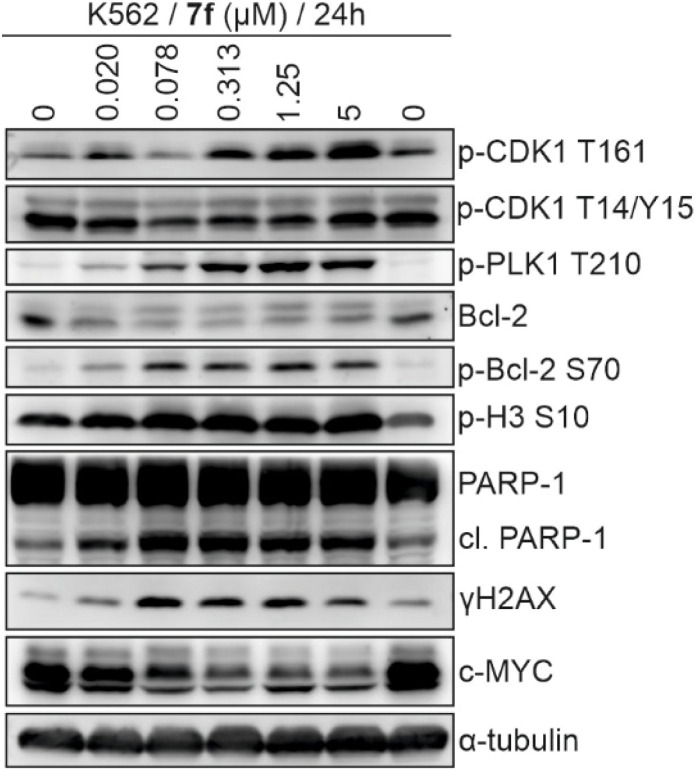
Immunoblotting analysis of mitotic and apoptotic markers in K562 cells treated with compound 7f. α-tubulin levels were detected to verify equal protein loading.

To distinguish between the G2 and M phase of the cell cycle, several markers of mitotic progression were further evaluated at the protein level by immunoblotting (Fig. 5). A crucial step of mitotic entry^56^ is the activation of several mitotic kinases, including cyclin-dependent kinase 1 (CDK1) and polo-like kinase 1 (Plk-1). Activation of CDK1 requires phosphorylation of T161 residue by CAK complex and dephosphorylation of T14/Y15 by Cdc25 phosphatases. Activating phosphorylation of Plk-1 at T210 is necessary for the proper function of this kinase.

Changes in the phosphorylation of these residues in CDK1 as well as Plk-1 were detected after 24 h treatment with 7f in a concentration-dependent manner (Fig. 5). Analysis of Bcl-2 protein *via* immunoblotting showed a slowly migrating band attributed to phosphorylated Bcl-2. This result was further confirmed using a specific antibody against S70-phosphorylated Bcl-2, clearly showing a dose-dependent increase of this form of Bcl-2, which belongs to another well-known marker of ongoing mitosis,^57^ as well as histone H3 phosphorylated at S10, whose levels were also rising with increasing concentrations of 7f.

**Fig. 6:**
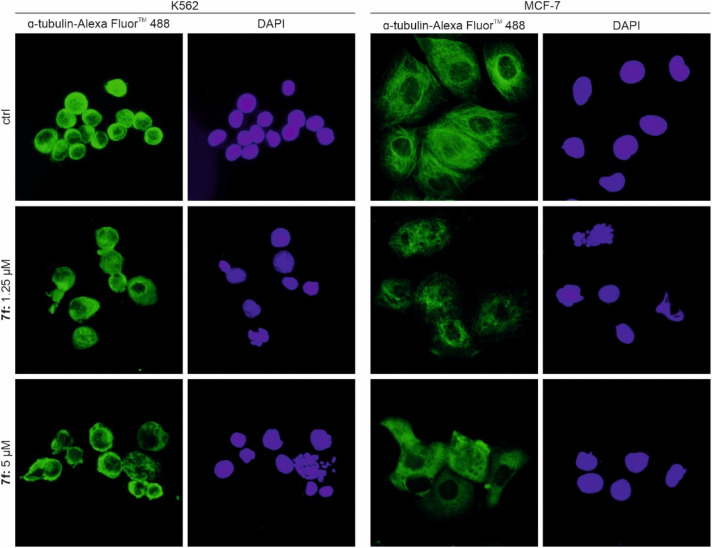
Immunofluorescence staining of K562 and MCF-7 cells upon 24 h treatment with indicated concentrations of 7f. α-tubulin was visualized by Alexa Fluor™ 488-conjugated antibody, and cell nuclei by DAPI.

The induction of mitotic arrest, accompanied by morphological changes, may indicate a disruption of cytoskeletal integrity. Although pyrazole derivatives are not commonly associated with tubulin inhibition, a few have been reported to act as tubulin-targeting anticancer agents.^58–61^ Therefore, immunofluorescence labelling of α-tubulin after 7f treatment was performed in K562 leukemic cells as well as in MCF-7 breast cancer cells (Fig. 6). The initial experiment demonstrated that 7f at micromolar concentrations disrupts the structure of microtubules and acts as a destabilising agent.

**Fig. 7:**
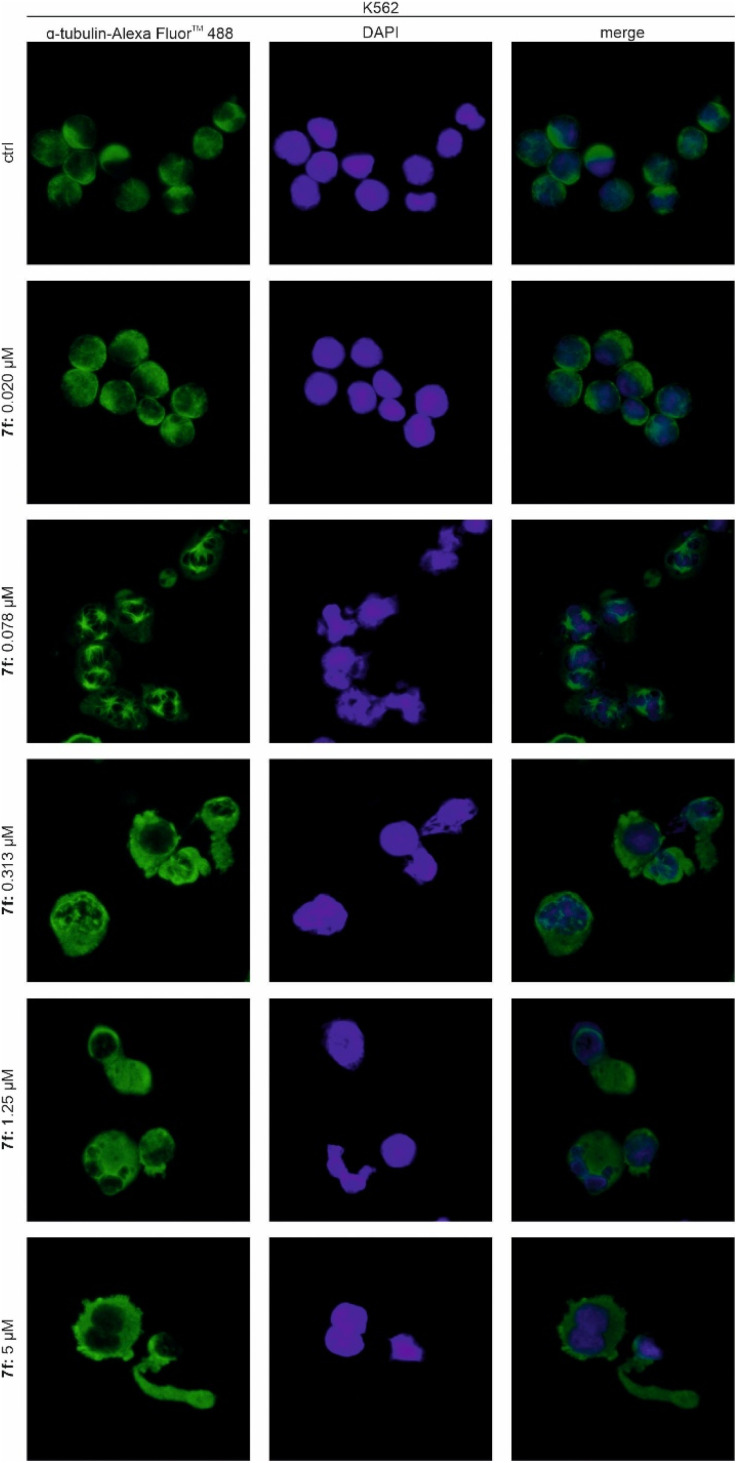
Immunofluorescence staining of K562 cells upon 24 h treatment with indicated concentrations of 7f. α-tubulin was visualized by Alexa Fluor™ 488-conjugated antibody, and cell nuclei by DAPI.

To further evaluate the concentration-dependent effect of 7f on the microtubule cytoskeleton, K562 cells were seeded on poly-l-lysine-coated cover slides to improve their attachment to the surface and subsequently treated with 7f. Immunofluorescence labelling of α-tubulin (Fig. 7) showed that nanomolar concentrations of 7f induced multipolar spindle formation, a common consequence of disruption of microtubule dynamics leading to aberrant mitosis. At higher concentrations, microtubules were disrupted, and the usual round cell morphology of K562 cells was altered to a significantly elongated form with nuclei located at one pole of the cell.

In addition to changes in cell cycle phase distribution, flow cytometry analysis revealed that 7f treatment increased the number of sub-G1 cells undergoing cell death (Fig. 4A). This was further confirmed *via* detection of massive PARP-1 cleavage, a common marker of ongoing apoptosis, and also increased phosphorylation of histone H2AX at S139 (γH2AX) in the treated samples (Fig. 5). The presence of γH2AX highlights a cellular response to DNA damage that in this case can be a consequence of genomic instability caused by alterations in microtubule dynamics and mitotic defects. Cell death probably also affected expression of several important proteins including common oncogene c-MYC whose levels dramatically decreased after treatment with nanomolar concentrations of 7f (Fig. 5). Moreover, already after 24 h treatment with 7f we observed an increasing population of cells characterized by >4N DNA amount in comparison to the untreated control (Fig. 4A). As the mitotic defects can prevent the proper chromosome segregation and lead to endoreduplication, the duration of treatment was prolonged to 48 h and subsequent analysis revealed that longer exposure of K562 cells to 7f expanded the population having 8N DNA content (Fig. 4A) proving the hypothesis. Analysis of BrdU-pulse labelled K562 cells treated with 0.313 µM 7f for 24 and 48 h also confirmed that DNA replication is maintained (BrdU-FITC-positive cells) in a significant fraction of the cells leading, in the absence of proper cell division, to polyploidy (SI, Fig. S1).

## Experimental

### Chemistry

#### General

All chemicals were purchased from Sigma-Aldrich and Fluorochem and were used as received without further purification. Organic solvents were purified and dried by standard methods.^62^ Melting points were determined on a Reichert–Kofler hot-stage microscope or in capillary tubes on an Electrothermal MEL-TEMP^®^ capillary melting point apparatus and are uncorrected. Mass spectra were obtained on a Shimadzu LCMS 2020 Single Quadrupole Liquid Chromatograph Mass Spectrometer. IR spectra of samples prepared as KBr pellets were recorded on a Bruker Tensor 27 spectrometer or on a Bruker Vertex v70 FTIR spectrometer equipped with a diamond ATR accessory and are reported in wavenumbers (cm^−1^). HRMS spectra were recorded with a Bruker micrOTOF-QIII spectrometer (ESI). ^1^H NMR, ^13^C NMR and ^15^N NMR spectra of CDCl_3_ or DMSO-*d*_6_ solutions at 25 °C were recorded on either a Bruker Avance III 400 instrument (400 MHz for ^1^H, 101 MHz for ^13^C) using a directly detecting BBO probe or on a Bruker Avance III 700 instrument (700 MHz for ^1^H, 176 MHz for ^13^C) equipped with a 5 mm TCI ^1^H–^13^C/^15^N/D z-gradient cryoprobe. The solvent (residual) signals were used as internal standards which were related to TMS with *δ* 7.26 ppm (^1^H, CDCl_3_), *δ* 2.49 ppm (^1^H, DMSO-*d*_6_), *δ* 77.00 ppm (^13^C, CDCl_3_), and *δ* 39.50 ppm (^13^C, DMSO-*d*_6_). ^15^N NMR spectra (41 MHz or 71 MHz) were obtained on a Bruker Avance III 400 or Bruker Avance III 700 instrument and were referenced against neat external nitromethane. ^19^F NMR spectra (376 MHz, absolute referencing *via Ξ* ratio) were obtained on a Bruker Avance III 400 using a directly detecting BBO probe. The full and unambiguous assignments of the ^1^H, ^13^C, and ^15^N NMR resonances were achieved by the combined application of NMR spectroscopic techniques such as ^1^H–^13^C HMBC, ^1^H–^13^C HSQC, ^1^H–^15^N HMBC, ^1^H–^1^H COSY, and others.

Diffraction data for compound 7f were collected at low temperature (150 K) on a Rigaku, XtaLAB Synergy, Dualflex, HyPix diffractometer using monochromated Cu-Kα radiation (*λ* = 1.54184 Å). The crystal structure was solved with the SIR2011 structure solution program^63^ using direct methods and refined with the ShelXL refinement package^64^ using least squares minimisation. All nonhydrogen atoms were refined in anisotropic approximation. The hydrogen atom involved in the formation of H-bond were refined isotropically; all other H-atoms were refined by riding model with *U*_iso_(H) = 1.2*U*_eq_(C). Crystal data: orthorhombic, *a* = 5.90879(4), *b* = 10.20119(8), *c* = 21.8526(2) Å; *V* = 1317.20(2) Å^3^, *Z* = 4, *µ* = 0.828 mm^−1^, *D*_calc_ = 1.464 g cm^−3^; space group is *P*2_1_2_1_2_1_. The final *R*_1_ was 0.0284 (*I* > 2*σ*(*I*)) and w*R*_2_ was 0.0767 (all data). For further details, see crystallographic data for compound 7f deposited at the Cambridge Crystallographic Data Centre. Deposition Number (https://www.ccdc.cam.ac.uk/services/structures) CCDC 2524805.

### General procedure for the synthesis of compounds 2a–f

A solution of appropriate 1-phenyl-1*H*-pyrazol-3-ol (1a–f)^44–47^ (7.2 mmol) in dry DMF (20 mL) was cooled to 0 °C under an inert atmosphere, and NaH (60% dispersion in mineral oil, 288 mg, 7.2 mmol) was added portion-wise. After stirring for 15 min, benzyl chloride (0.82 mL, 7.2 mmol) was added dropwise. The reaction mixture was stirred at 60 °C for 1 h, then poured into water and extracted with ethyl acetate. The organic layers were combined, washed with brine, dried over Na_2_SO_4_, filtered, and the solvent was evaporated. The residue was purified by column chromatography (SiO_2_, eluent : ethyl acetate/*n*-hexane, 1 : 7, v/v) to give pure compounds 2a–f.

#### 3-(Benzyloxy)-1-phenyl-1*H*-pyrazole (2a)

Previously reported by Arbačiauskienė *et al.*^49^ The data are consistent with that reported.

#### 3-(Benzyloxy)-1-(4-methylphenyl)-1*H*-pyrazole (2b)

Brownish solid; yield 87% (1.654 g); mp 57.1–59.9 °C. IR (KBr, *v*_max_, cm^−1^): 3148, 3030 (CH_arom_), 2932, 2881 (CH_aliph_), 1548, 1518, 1485, 1451, 1353 (C–O–C, C=C, C–N), 1266, 1234, 1051, 1022, 828, 806, 733, 698 (CH=CH of benzenes). ^1^H NMR (700 MHz, CDCl_3_): *δ* 2.35 (s, 3H, CH_3_), 5.30 (s, 2H, OCH_2_), 5.85–5.88 (m, 1H, 4-H), 7.16–7.22 (m, 2H, NPh 3,5-H), 7.29–7.34 (m, 1H, CPh 4-H), 7.34–7.40 (m, 2H, CPh 3,5-H), 7.44–7.52 (m, 4H, NPh 2,6-H, CPh 2,6-H), 7.65–7.68 (m, 1H, 5-H). ^13^C NMR (176 MHz, CDCl_3_): *δ* 20.9 (OCH_3_), 70.9 (OCH_2_), 93.6 (C-4); 117.9 (NPh C-2,6), 127.7 (C-5), 128.10 (CPh C-4), 128.15 (CPh C-2,6), 128.5 (CPh C-3,5), 129.9 (NPh C-3,5), 135.1 (NPh C-4), 137.1 (CPh C-1), 138.1 (NPh C-1), 164.2 (C-3). ^15^N NMR (41 MHz, CDCl_3_): *δ* −185.7 (N-1), N-2 was not found. MS *m*/*z* (%): 265 ([M + H]^+^, 100). HRMS (ESI) for C_17_H_16_N_2_ONa ([M + Na]^+^): calcd 287.1154, found 287.1155.

#### 3-(Benzyloxy)-1-(4-methoxyphenyl)-1*H*-pyrazole (2c)

White solid; yield 82% (1.653 g); mp 90.9–92.2 °C. IR (KBr, *v*_max_, cm^−1^): 3031 (CH_arom_), 2949, 2831 (CH_aliph_), 1543, 1519, 1500, 1483, 1352, 1250, 1051, 1025 (C–O–C, C=C, C–N), 913, 834, 826, 746, 735, 670, 549 (CH=CH of benzenes). ^1^H NMR (700 MHz, CDCl_3_): *δ* 3.83 (s, 3H, OCH_3_), 5.30 (s, 2H, OCH_2_), 5.87–5.91 (m, 1H, 4-H), 6.95–6.97 (m, 2H, NPh 3,5-H), 7.32–7.35 (m, 1H, CPh 4-H), 7.36–7.42 (m, 2H, CPh 3,5-H), 7.46–7.50 (m, 2H, CPh 2,6-H), 7.50–7.54 (m, 2H, NPh 2,6-H), 7.61–7.64 (m, 1H, 5-H). ^13^C NMR (176 MHz, CDCl_3_): *δ* 55.7 (OCH_3_), 71.0 (OCH_2_), 93.3 (C-4), 114.6 (NPh C-3,5), 119.8 (NPh C-2,6), 127.8 (C-5), 128.1 (CPh C-4), 128.2 (CPh 2,6), 128.6 (CPh C-3,5), 134.2 (NPh C-1), 137.2 (CPh C-1), 157.6 (NPh C-4), 164.2 (C-3). ^15^N NMR (71 MHz, CDCl_3_): *δ* −186.4 (N-1), N-2 was not found. MS *m/z* (%): 281 ([M + H]^+^, 100). HRMS (ESI) for C_17_H_16_N_2_O_2_Na ([M + Na]^+^): calcd 303.1104, found 303.1104.

#### 3-(Benzyloxy)-1-(4-fluorophenyl)-1*H*-pyrazole (2d)

Brown solid; yield 97% (1.871 g); mp 55.3–56.5 °C. IR (KBr, *v*_max_, cm^−1^): 3139 (CH_arom_), 2926 (CH_aliph_), 1553, 1539, 1490, 1488, 1391, 1361, 1234, 1211, 1065, 1036 (C–F, C–O–C, C=C, C–N), 1029, 836, 756, 745, 733, 695, 612 (CH=CH of benzenes). ^1^H NMR (400 MHz, CDCl_3_): *δ* 5.30 (s, 2H, CH_2_), 5.89–5.93 (m, 1H, 4-H), 7.06–7.14 (m, 2H, NPh 3,5-H), 7.29–7.35 (m, 1H, CPh 4-H), 7.35–7.42 (m, 2H, CPh 3,5-H), 7.46–7.51 (m, 2H, CPh 2,6-H), 7.53–7.59 (m, 2H, NPh 2,6-H), 7.63–7.67 (m, 1H, 5-H). ^13^C NMR (101 MHz, CDCl_3_): *δ* 71.0 (CH_2_), 94.2 (C-4), 116.2 (d, ^2^*J*_C,F_ = 22.9 Hz, NPh C-3,5), 119.6 (d, ^3^*J*_C,F_ = 8.2 Hz, NPh C-2,6), 127.9 (C-5), 128.14 (CPh C-2,6), 128.18 (CPh C-4), 128.6 (CPh C-3,5), 136.7 (d, ^4^*J*_C,F_ = 2.7 Hz, NPh C-1), 137.0 (CPh C-1), 160.5 (d, ^1^*J*_C,F_ = 244.5 Hz, NPh C-4), 164.4 (C-3). ^15^N NMR (41 MHz, CDCl_3_): *δ* −187.7 (N-1), N-2 was not found. MS *m*/*z* (%): 269 ([M + H]^+^, 100). HRMS (ESI) for C_16_H_13_FN_2_ONa ([M + Na]^+^): calcd 291.0904, found 291.0904.

#### 3-(Benzyloxy)-1-(3-fluorophenyl)-1*H*-pyrazole (2e)

Brown solid; yield 90% (1.737 g); mp 58.0–61.0 °C. IR (KBr, *v*_max_, cm^−1^): 3035 (CH_arom_), 2960, 2892 (CH_aliph_), 1737, 1614, 1598, 1548, 1501, 1480, 1458, 1356, 1265, 1237, 1187, 1026 (C=C, C–N, C–O–C, C–F), 981, 951, 844, 761, 751, 735, 696, 522 (CH=CH of benzenes). ^1^H NMR (700 MHz, CDCl_3_): *δ* 5.33 (s, 2H, CH_2_), 5.95 (d, ^3^*J* = 2.6 Hz, 1H, 4-H), 6.88–6.92 (m, 1H, NPh 4-H), 7.33–7.36 (m, 3H, CPh 3,4,5-H), 7.39–7.41 (m, 3H, NPh 2,5,6-H), 7.49–7.51 (m, 2H, CPh 2,6-H), 7.73 (d, ^3^*J* = 2.6 Hz, 1H, 5-H). ^13^C NMR (176 MHz, CDCl_3_): *δ* 71.0 (CH_2_), 95.0 (C-4), 105.6 (d, ^2^*J*_C,F_ = 26.4 Hz, NPh C-2), 112.0 (d, ^2^*J*_C,F_ = 21.4 Hz, NPh C-4), 112.8 (d, ^4^*J*_C,F_ = 2.8 Hz, NPh C-6), 128.0 (CPh C-4), 128.21 (CPh C-2,6), 128.22 (^5^*J*_C,F_ = 2.6 Hz, C-5), 128.6 (CPh C-3,5), 130.7 (d, ^3^*J*_C,F_ = 9.2 Hz, NPh C-5), 137.0 (CPh C-1), 141.7 (d, ^3^*J*_C,F_ = 7.9 Hz, NPh C-1), 163.5 (d, ^1^*J*_C,F_ = 245.8 Hz, NPh C-3), 164.5 (C-3). ^19^F NMR (376 MHz, CDCl_3_): *δ* −111.2. MS *m*/*z* (%): 269 ([M + H]^+^, 100). HRMS (ESI) for C_16_H_13_FN_2_ONa ([M + Na]^+^): calcd 291.0905, found 291.0904.

#### 3-(Benzyloxy)-1-(2-fluorophenyl)-1*H*-pyrazole (2f)

Previously reported by Mueller *et al.*^65^ Brown amorphous solid; yield 88% (1.698 g). IR (KBr, *v*_max_, cm^−1^): 3008 (CH_arom_), 2978, 2940, 2864 (CH_aliph_), 1692, 1646, 1469, 1404, 1367, 1302, 1265, 1211, 1152 (C=C, C–N, C–O–C, C–F), 931, 771, 525 (CH=CH of benzenes). ^1^H NMR (700 MHz, CDCl_3_): *δ* 5.31 (s, 2H, CH_2_), 5.94 (d, *J* = 2.6 Hz, 1H, 4-H), 7.14–7.22 (m, 3H, NPh 3,4,5-H), 7.31–7.34 (m, 1H, CPh 4-H), 7.37–7.40 (m, 2H, CPh 3,5-H), 7.47–7.50 (m, 2H, CPh 2,6-H), 7.84 (d, ^3^*J* = 2.6 Hz, 1H, 5-H), 7.87–7.90 (m, 1H, NPh 6-H). ^13^C NMR (176 MHz, CDCl_3_): *δ* 71.0 (CH_2_), 94.4 (d, ^5^*J*_C,F_ = 2.4 Hz, C-4), 116.8 (d, ^2^*J*_C,F_ = 20.4 Hz, NPh C-3), 123.3 (CPh C-4), 125.0 (d, ^4^*J*_C,F_ = 3.6 Hz, NPh C-5), 126.3 (d, ^3^*J*_C,F_ = 7.8 Hz, NPh C-6), 128.15 (CPh C-2,6), 128.16 (d, ^4^*J*_C,F_ = 1.7 Hz, C-5), 128.5 (d, ^2^*J*_C,F_ = 8.9 Hz, NPh C-1), 128.6 (CPh C-3,5), 132.6 (d, ^3^*J* = 12.4 Hz, NPh C-4), 137.0 (CPh C-1), 153.2 (d, ^1^*J*_C,F_ = 247.6 Hz, NPh C-2), 164.2 (C-3). MS *m*/*z* (%): 269 ([M + H]^+^, 100). HRMS (ESI) for C_16_H_13_N_2_OFNa ([M + Na]^+^): calcd 291.0904, found 291.0909.

### General procedure for the synthesis of compounds 3a–f

A solution of phosphorus oxychloride (0.37 mL, 4 mmol) in DMF (0.30 mL, 4 mmol) was cooled to 0 °C for 15 min, then appropriate pyrazole 2a–f (1 mmol) was added. The reaction mixture was stirred at 60 °C for 1 hour, then diluted with a 10% Na_2_CO_3_ solution and extracted with ethyl acetate (three times, 10 mL each). The organic layers were combined, washed with brine, dried over Na_2_SO_4_, filtered, and the solvent was evaporated. The residue was purified by flash column chromatography (SiO_2_, eluent : ethyl acetate/*n*-hexane, 1 : 2, v/v) to give pure compounds 3a–f.

#### 3-(Benzyloxy)-1-phenyl-1*H*-pyrazole-4-carbaldehyde (3a)

Previously reported by Arbačiauskienė *et al.*^49^ The data are consistent with that reported.

#### 3-(Benzyloxy)-1-(4-methylphenyl)-1*H*-pyrazole-4-carbaldehyde (3b)

White solid; yield 84% (245 mg); mp 123.0–123.6 °C. IR (KBr, *v*_max_, cm^−1^): 3098 (CH_arom_), 2820 (CH_aliph_), 1667 (C=O), 1558, 1506, 1364, 1225, 1204 (C–O–C, C=C, C–N), 1010, 817, 735 (CH=CH of benzenes). ^1^H NMR (400 MHz, CDCl_3_): *δ* 2.39 (s, 3H, CH_3_), 5.44 (s, 2H, CH_2_), 7.23–7.29 (m, 2H, NPh 3,5-H), 7.32–7.38 (m, 1H, CPh 4-H), 7.38–7.44 (m, 2H, CPh 3,5-H), 7.49–7.55 (m, 4H, NPh 2,6-H, CPh 2,6-H), 8.22 (s, 1H, 5-H), 9.87 (s, 1H, CHO). ^13^C NMR (101 MHz, CDCl_3_): *δ* 21.1 (CH_3_), 71.2 (CH_2_), 111.3 (C-4), 118.9 (NPh C-2,6), 128.3 (CPh C-2,6), 128.4 (CPh C-4), 128.6 (CPh C-3,5), 129.2 (C-5), 130.2 (NPh C-3,5), 136.3 (CPh C-1), 136.9 (NPh C-1), 137.4 (NPh C-4), 163.7 (C-3), 183.4 (CHO). ^15^N NMR (41 MHz, CDCl_3_): *δ* −178.8 (N-1), N-2 was not found. MS *m*/*z* (%): 293 ([M + H]^+^, 100). HRMS (ESI) for C_18_H_16_N_2_O_2_Na ([M + Na]^+^): calcd 315.1104, found 315.1104.

#### 3-(Benzyloxy)-1-(4-methoxyphenyl)-1*H*-pyrazole-4-carbaldehyde (3c)

White solid: yield 79% (243 mg); mp 140.0–141.7 °C. IR (KBr, *v*_max_, cm^−1^): 3093, 3034 (CH_arom_), 2835 (CH_aliph_), 1662 (C=O), 1557, 1520, 1505, 1497, 1359, 1249, 1212, 1202 (C–O–C, C=C, C–N) 829, 627 (CH=CH of benzenes). ^1^H NMR (700 MHz, CDCl_3_): *δ* 3.85 (s, 3H, CH_3_), 5.43 (s, 2H, CH_2_), 6.95–7.00 (m, 2H, NPh 3,5-H), 7.33–7.37 (m, 1H, CPh 4-H), 7.39–7.43 (m, 2H, CPh 3,5-H), 7.50–7.53 (m, 2H, CPh 2,6-H), 7.54–7.57 (m, 2H, NPh 2,6-H), 8.16 (s, 1H, 5-H), 9.86 (s, 1H, CHO). ^13^C NMR (176 MHz, CDCl_3_): *δ* 55.7 (CH_3_), 71.2 (CH_2_), 111.2 (C-4), 114.8 (NPh C-3,5), 120.6 (NPh C-2,6), 128.3 (CPh C-2,6), 128.4 (CPh C-4), 128.6 (CPh C-3,5), 129.2 (C-5), 132.8 (NPh C-1), 136.3 (CPh C-1), 158.9 (NPh C-4), 163.7 (C-3), 183.4 (CHO). ^15^N NMR (71 MHz, CDCl_3_): *δ* −179.1 (N-1), N-2 was not found. MS *m*/*z* (%): 309 ([M + H]^+^, 100). HRMS (ESI) for C_18_H_16_N_2_O_3_Na ([M + Na]^+^): calcd 331.1053, found 331.1053.

#### 3-(Benzyloxy)-1-(4-fluorophenyl)-1*H*-pyrazole-4-carbaldehyde (3d)

White solid; yield 82% (244 mg); mp 171.9–172.9 °C. IR (KBr, *v*_max_, cm^−1^): 3128, 3097 (CH_arom_), 2963, 2954, 2916 (CH_aliph_), 1662 (C=O), 1559, 1519, 1505, 1495, 1454, 1357, 1297, 1226, 1218, 1205, (C–F, C–O–C, C=C, C–N), 836, 830, 208, 696 (CH=CH of benzenes). ^1^H NMR (700 MHz, CDCl_3_): *δ* 5.44 (s, 2H, CH_2_), 7.14–7.20 (m, 2H, NPh 3,5-H), 7.34–7.38 (m, 1H, CPh 4-H), 7.39–7.43 (m, 2H, CPh 3,5-H), 7.49–7.54 (m, 2H, CPh 2,6-H), 7.60–7.65 (m, 2H, NPh 2,6-H), 8.20 (s, 1H, 5-H), 9.88 (s, 1H, CHO). ^13^C NMR (176 MHz, CDCl_3_): *δ* 71.3 (CH_2_), 111.7 (C-4), 116.6 (d, ^2^*J*_C,F_ = 23.2 Hz, NPh C-3,5), 120.8 (d, ^3^*J*_C,F_ = 8.4 Hz, NPh C-2,6), 128.3 (CPh C-2,6), 128.5 (CPh C-4), 128.7 (CPh C-3,5), 129.5 (C-5), 135.5 (d, ^4^*J*_C,F_ = 3.0 Hz, NPh C-1), 136.2 (CPh C-1), 161.6 (d, ^1^*J*_C,F_ = 247.7 Hz, NPh C-4), 163.8 (C-3), 183.4 (CHO). ^15^N NMR (71 MHz, CDCl_3_): *δ* −181.0 (N-1), N-2 was not found. MS *m*/*z* (%): 297 ([M + H]^+^, 100). HRMS (ESI) for C_17_H_13_FN_2_O_2_Na ([M + Na]^+^): calcd 319.0853, found 319.0853.

#### 3-(Benzyloxy)-1-(3-fluorophenyl)-1*H*-pyrazole-4-carbaldehyde (3e)

White solid; yield 81% (240 mg); mp 116.5–118.9 °C. IR (KBr, *v*_max_, cm^−1^): 3102, 3072 (CH_arom_), 2850 (CH_aliph_), 1662 (C=O), 1605, 1565, 1509, 1451, 1362,1264, 1234, 1195 (C–F, C–O–C, C=C, C–N), 877, 743, 733, 674, 458 (CH=CH of benzenes). ^1^H NMR (700 MHz, CDCl_3_): *δ* 5.43 (s, 2H, CH_2_), 6.99–7.02 (m, 1H, NPh 4-H), 7.33–7.36 (m, 1H, CPh 4-H), 7.38–7.45 (m, 5H, CPh 3,5-H, NPh 2,5,6-H), 7.50–7.52 (m, 2H, CPh 2,6-H), 8.25 (s, 1H, 5-H), 9.87 (s, 1H, CHO). ^13^C NMR (176 MHz, CDCl_3_): *δ* 71.3 (CH_2_), 106.8 (d, ^2^*J*_C,F_ = 26.5 Hz, NPh C-2), 112.0 (C-4), 113.8 (d, ^4^*J*_C,F_ = 3.2 Hz, NPh C-6), 114.0 (d, ^2^*J*_C,F_ = 21.3 Hz, NPh C-4), 128.3 (CPh C-2,6), 128.5 (CPh C-4), 128.6 (CPh C-3,5), 129.6 (C-5), 131.0 (d, ^3^*J*_C,F_ = 9.1 Hz, NPh C-5), 136.1 (CPh C-1), 140.4 (d, ^3^*J*_C,F_ = 10.2 Hz, NPh C-1), 163.3 (d, ^1^*J*_C,F_ = 247.3 Hz, NPh C-3), 163.6 (C-3), 183.3 (CHO). ^19^F NMR (376 MHz, CDCl_3_): *δ* −110.1. MS *m*/*z* (%): 297 ([M + H]^+^, 100). HRMS (ESI) for C_17_H_13_FN_2_O_2_Na ([M + Na]^+^): calcd 319.0853, found 319.0853.

#### 3-(Benzyloxy)-1-(2-fluorophenyl)-1*H*-pyrazole-4-carbaldehyde (3f)

White solid; yield 83% (246 mg); mp 94.0–96.5 °C. IR (KBr, *v*_max_, cm^−1^): 3069, 3033 (CH_arom_), 2965, 2948, 2821 (CH_aliph_), 1681 (C=O), 1561, 1501, 1454, 1361, 1230, 1208, 1199, 1112, (C–F, C–O–C, C=C, C–N), 978, 968, 940, 756, 739, 697, 612 (CH=CH of benzenes). ^1^H NMR (700 MHz, CDCl_3_): *δ* 5.44 (s, 2H, CH_2_), 7.22–7.31 (m, 3H, NPh 3,4,5-H), 7.34–7.37 (m, 1H, CPh 4-H), 7.39–7.42 (m, 2H, CPh 3,5-H), 7.50–7.53 (m, 2H, CPh 2,6-H), 7.89 (m, 1H, NPh 6-H), 8.37 (d, *J* = 1.8 Hz, 1H, 5-H), 9.89 (s, 1H, CHO). ^13^C NMR (176 MHz, CDCl_3_): *δ* 71.3 (CH_2_), 111.8 (d, ^5^*J*_C,F_ = 1.9 Hz, C-4), 117.1 (d, ^2^*J*_C,F_ = 20.2 Hz, NPh C-3), 124.0 (C-5), 125.2 (d, ^4^*J*_C,F_ = 3.7 Hz, NPh C-5), 127.3 (d, ^2^*J*_C,F_ = 8.5 Hz, NPh C-1), 128.3 (CPh C-2,6), 128.4 (d, ^3^*J*_C,F_ = 7.9 Hz, NPh C-6), 128.5 (CPh C-4), 128.7 (CPh C-3,5), 134.5 (d, ^3^*J*_C,F_ = 12.3 Hz, NPh C-4), 136.2 (CPh C-1), 153.6 (d, ^1^*J*_C,F_ = 249.6 Hz, NPh C-2), 163.3 (C-3), 183.4 (CHO). ^19^F NMR (376 MHz, CDCl_3_): *δ* −109.84. MS *m*/*z* (%)*:* 297 ([M + H]^+^, 100). HRMS (ESI) for C_17_H_13_FN_2_O_2_Na ([M + Na]^+^): calcd 319.0853, found 319.0853.

### General procedure for the synthesis of compounds 4a–f

Into the solution of appropriate 3-benzyloxypyrazole 3a–f (1 mmol) in toluene (10 mL), TFA (10 mL) was added. The mixture was stirred at room temperature for 18 h. Toluene and TFA were evaporated. The residue was purified by flash column chromatography (SiO_2_, eluent : ethyl acetate/*n*-hexane, 1 : 1, v/v) to give pure compounds 4a–f.

#### 3-Hydroxy-1-phenyl-1*H*-pyrazole-4-carbaldehyde (4a)

Previously reported by Arbačiauskienė *et al.*^49^ The data are consistent with that reported.

#### 3-(Hydroxy)-1-(4-methylphenyl)-1*H*-pyrazole-4-carbaldehyde (4b)

Yellowish solid; yield 86% (174 mg); mp 213.7–214.9 °C. IR (KBr, *v*_max_, cm^−1^): 3107 (OH), 3039 (CH_arom_), 2950, 2852, 2707, 2574 (CH_aliph_), 1681 (C=O), 1601, 1587, 1537, 1524, 1319, 1220, (C–O–C, C=C, C–N), 812 (CH=CH of benzene). ^1^H NMR (400 MHz, CDCl_3_): *δ* 2.40 (s, 3H, CH_3_), 7.23–7.27 (m, 2H, Ph 3,5-H), 7.49–7.56 (m, 2H, Ph 2,6-H), 8.12 (s, 1H, 5-H), 9.89 (s, 1H, CHO). ^13^C NMR (101 MHz, CDCl_3_): *δ* 21.1 (CH_3_), 109.8 (C-4), 119.3 (Ph C-2,6), 129.6 (C-5), 130.3 (Ph C-3,5), 136.6 (Ph C-1), 137.9 (Ph C-4), 163.7 (C-3), 186.0 (CHO). ^15^N NMR (41 MHz, CDCl_3_): *δ* −178.3 (N-1), N-2 was not found. MS *m*/*z* (%): 203 ([M + H]^+^, 100). HRMS (ESI) for C_11_H_10_N_2_O_2_Na ([M + Na]^+^): calcd 225.0635, found 225.0634.

#### 3-(Hydroxy)-1-(4-methoxyphenyl)-1*H*-pyrazole-4-carbaldehyde (4c)

Brown solid; yield 92% (200 mg); mp 185.6–190.0 °C. IR (KBr, *v*_max_, cm^−1^): 3272 (OH), 3122 (CH_arom_), 2962 (CH_aliph_), 1658 (C=O), 1574, 1517, 1498, 1459, 1428, 1304, 1252, 1177, 1169, (C–O–C, C=C, C–N) 1042, 1024, 828, 794 (CH=CH of benzene). ^1^H NMR (400 MHz, CDCl_3_): *δ* 3.85 (s, 1H, CH_3_), 6.96–7.01 (m, 2H, Ph 3,5-H), 7.53–7.59 (m, 2H, Ph 2,6-H), 8.06 (s, 1H, 5-H), 8.59–9.25 (br s, 1H, OH), 9.89 (s, 1H, CHO). ^13^C NMR (101 MHz, CDCl_3_): *δ* 55.7 (OCH_3_), 109.6 (C-4), 114.9 (Ph C-3,5), 121.0 (Ph C-2,6), 129.5 (C-5), 132.5 (Ph C-1), 159.2 (Ph C-4), 163.7 (C-3), 186.1 (CHO). ^15^N NMR (41 MHz, CDCl_3_): *δ* −178.4 (N-1), N-2 was not found. MS *m*/*z* (%): 219 ([M + H]^+^, 100). HRMS (ESI) for C_11_H_10_N_2_O_3_Na ([M + Na]^+^): calcd 241.0584, found 241.0584.

#### 1-(4-Fluorophenyl)-3-(hydroxy)-1*H*-pyrazole-4-carbaldehyde (4d)

White solid; yield 84% (173 mg); mp 210.2–210.5 °C. IR (KBr, *v*_max_, cm^−1^): 3102 (CH_arom_), 3077 (CH_aliph_), 1673 (C=O), 1594, 1539, 1521, 1316, 1223, (C–F, C–O–C, C=C, C–N), 1156, 827 (CH=CH of benzene). ^1^H NMR (400 MHz, CDCl_3_): *δ* 7.12–7.22 (m, 2H, Ph 3,5-H), 7.61–7.68 (m, 2H, Ph 2,6-H), 8.11 (s, 1H, 5-H), 8.30–9.12 (br s, 1H, OH), 9.91 (s, 1H, CHO). ^13^C NMR (101 MHz, CDCl_3_): *δ* 110.0 (C-4), 116.7 (d, ^2^*J*_C,F_ = 23.2 Hz, Ph C-3,5), 121.3 (d, ^3^*J*_C,F_ = 8.6 Hz, Ph C-2,6), 129.8 (C-5), 135.3 (d, ^4^*J*_C,F_ = 2.9 Hz, Ph C-1), 161.9 (d, ^1^*J*_C,F_ = 284.4 Hz, Ph C-4), 163.7 (C-3), 186.2 (CHO). ^15^N NMR (41 MHz, CDCl_3_): *δ* −180.2 (N-1), N-2 was not found. MS *m*/*z* (%): 207 ([M + H]^+^, 100). HRMS (ESI) for C_10_H_7_FN_2_O_2_Na ([M + Na]^+^): calcd 229.0384, found 229.0384.

#### 1-(3-Fluorophenyl)-3-hydroxy-1*H*-pyrazole-4-carbaldehyde (4e)

White solid; yield 83% (171 mg); mp 208–210 °C. IR (KBr, *v*_max_, cm^−1^): 3103 (CH_arom_), 2949 (CH_aliph_), 1673, 1590, 1522, 1503, 1451, 1317, 1259, 1243, 1222, 1156 (C–F, C=C, C–N), 828, 812, 722, 646, 608, 506 (CH=CH of benzene). ^1^H NMR (400 MHz, CDCl_3_): *δ* 7.04–7.08 (m, 1H, Ph 4-H), 7.26 (s, 1H, Ph 2-H), 7.44–7.46 (m, 2H, Ph 5,6-H), 8.18 (s, 1H, 5-H), 8.69–8.86 (br s, 1H, OH), 9.92 (s, 1H, CHO). ^13^C NMR (101 MHz, CDCl_3_): *δ* 107.3 (d, ^2^*J*_C,F_ = 26.6 Hz, Ph C-2), 110.3 (C-4), 114.4 (d, ^4^*J*_C,F_ = 3.2 Hz, Ph C-6), 114.7 (d, ^2^*J*_C,F_ = 21.1 Hz, Ph C-4), 129.9 (C-5), 131.2 (d, ^3^*J*_C,F_ = 9.1 Hz, Ph C-5), 140.3 (d, ^3^*J*_C,F_ = 10.1 Hz, Ph C-1), 163.4 (d, ^1^*J*_C,F_ = 247.9 Hz, Ph C-3), 163.7 (C-3), 186.3 (CHO). ^19^F NMR (376 MHz, CDCl_3_): *δ* −125.3. MS *m*/*z* (%): 207 ([M + H]^+^, 100). HRMS (ESI) for C_10_H_7_FN_2_O_2_Na ([M + Na]^+^): calcd 229.0384, found 229.0385.

#### 1-(2-Fluorophenyl)-3-hydroxy-1*H*-pyrazole-4-carbaldehyde (4f)

White solid; yield 86% (177 mg), mp 188.5–191.3 °C. IR (KBr, *v*_max_, cm^−1^): 3143, 3065 (CH_arom_), 2947, 2833 (CH_aliph_), 1672, 1583, 1541, 1504, 1453, 1307, 1232, 1194, 1051, (C–F, C=C, C–N), 813, 795, 763, 655, 615, 465 (CH=CH of benzene). ^1^H NMR (700 MHz, CDCl_3_): *δ* 7.24–7.37 (m, 3H, Ph 3,4,5-H), 7.88 (t, ^3^*J* = 7.9 Hz, 1H, Ph 6-H), 8.31 (s, 1H, 5-H), 9.91 (s, 1H, CHO). ^13^C NMR (176 MHz, CDCl_3_): *δ* 110.3 (C-4), 117.1 (d, ^2^*J*_C,F_ = 20.4 Hz, Ph C-3), 124.3 (C-5), 125.4 (d, ^4^*J*_C,F_ = 3.8 Hz, Ph C-5), 127.0 (d, ^2^*J*_C,F_ = 8.5 Hz, Ph C-1), 128.8 (d, ^3^*J*_C,F_ = 7.9 Hz, Ph C-6), 134.7 (d, ^3^*J*_C,F_ = 12.3 Hz, Ph C-4), 153.7 (d, ^1^*J*_C,F_ = 249.3 Hz, Ph C-2), 163.3 (C-3), 186.2 (CHO). MS *m*/*z* (%): 207 ([M + H]^+^, 100). HRMS (ESI) for C_10_H_7_FN_2_O_2_Na ([M + Na]^+^): calcd 229.0384, found 229.0385.

### General procedure for the synthesis of compounds 5a–f

Appropriate 3-hydroxypyrazole 4a–f (1 mmol), trifluoromethanesulfonic anhydride (0.17 mL, 1 mmol), and TEA (0.17 mL, 1.2 mmol) were dissolved in DCM (5 mL), and stirred at room temperature for 1 h. The reaction mixture was poured into water and extracted with ethyl acetate. The combined organic layers were washed with brine and dried over Na_2_SO_4_; the solvent was evaporated. The residue was purified by flash column chromatography (SiO_2_, eluent : ethyl acetate/*n*-hexane, 1 : 6, v/v) to give pure compounds 5a–f.

#### 4-Formyl-1-phenyl-1*H*-pyrazol-3-yl trifluoromethanesulfonate (5a)

Previously reported by Arbačiauskienė *et al.*^49^ The data are consistent with that reported.

#### 4-Formyl-1-(4-methylphenyl)-1*H*-pyrazol-3-yl trifluoromethanesulfonate (5b)

White solid; yield 85% (332 mg); mp 89.6–90.6 °C. IR (KBr, *v*_max_, cm^−1^): 3138, 3100 (CH_arom_), 2924, 2865 (CH_aliph_), 1681 (C=O), 1557, 1520, 1461, 1429, 1236, 1215, 1136 (C–O–C, C=C, C–N, C–F), 885, 603 (CH=CH of benzene). ^1^H NMR (400 MHz, CDCl_3_): *δ* 2.42 (s, 3H, CH_3_), 7.28–7.35 (m, 2H, Ph 3,5-H), 7.50–7.58 (m, 2H, Ph 2,6-H), 8.35 (s, 1H, 5-H), 9.91 (s, 1H, CHO). ^13^C NMR (101 MHz, CDCl_3_): *δ* 21.2 (CH_3_), 114.6 (C-4), 118.7 (d, ^1^*J*_C,F_ = 321.3 Hz, CF_3_), 119.6 (Ph C-2,6), 130.5 (Ph C-3,5), 130.8 (C-5), 136.1 (Ph C-1), 139.1 (Ph C-4), 152.2 (C-3), 181.1 (CHO). ^15^N NMR (41 MHz, CDCl_3_): *δ* −170.3 (N-1), N-2 was not found. MS *m*/*z* (%): 335 ([M + H]^+^, 100). HRMS (ESI) for C_12_H_9_F_3_N_2_O_4_SNa ([M + Na]^+^): calcd 357.0127, found 357.0127.

#### 4-Formyl-1-(4-methoxyphenyl)-1*H*-pyrazol-3-yl trifluoromethanesulfonate (5c)

White solid; yield 77% (270 mg); mp 65.2–66.5 °C. IR (KBr, *v*_max_, cm^−1^): 3138, 3096 (CH_arom_), 2946, 2840 (CH_aliph_), 1683 (C=O), 1556, 1518, 1465, 1427, 1254, 1233, 1175, 1135 (C–O–C, C=C, C–N, C–F), 1027, 883, 832, 604 (CH=CH of benzene). ^1^H NMR (700 MHz, CDCl_3_): *δ* 3.87 (s, 3H, CH_3_), 6.98–7.04 (m, 2H, Ph 3,5-H), 7.54–7.59 (m, 2H, Ph 2,6-H), 8.29 (s, 1H, 5-H), 9.90 (s, 1H, CHO). ^13^C NMR (176 MHz, CDCl_3_): *δ* 55.8 (CH_3_), 114.5 (C-4), 115.0 (Ph C-3,5), 118.77 (d, ^1^*J*_C,F_ = 321.5 Hz, CF_3_), 121.3 (Ph C-2,6), 130.8 (C-5), 131.8 (Ph C-1), 152.1 (C-3), 160.0 (Ph C-4), 181.2 (CHO). ^15^N NMR (71 MHz, CDCl_3_): *δ* −170.7 (N-1), N-2 was not found. MS *m*/*z* (%): 351 ([M + H]^+^, 100). HRMS (ESI) for C_12_H_19_F_3_N_2_O_5_SNa ([M + Na]^+^): calcd 373.0078, found 373.0076.

#### 1-(4-Fluorophenyl)-4-formyl-1*H-*pyrazol-3-yl trifluoromethanesulfonate (5d)

White solid; yield 79% (267 mg); mp 80.2–80.4 °C. IR (KBr, *v*_max_, cm^−1^): 3132, 3093 (CH_arom_), 3077 (CH_aliph_), 1678 (C=O), 1557, 1517, 1463, 1434, 1392, 1252, 1229, 1203 (C–F, C–O–C, C=C, C–N), 1146, 954, 887, 838, 802, 768, 733, 617, 516 (CH=CH of benzene). ^1^H NMR (400 MHz, CDCl_3_): *δ* 7.19–7.25 (m, 2H, Ph 3,5-H), 7.63–7.69 (m, 2H, Ph 2,6-H), 8.35 (s, 1H, 5-H), 9.92 (s, 1H, CHO). ^13^C NMR (101 MHz, CDCl_3_): *δ* 114.9 (C-4), 117.0 (d, ^2^*J*_C,F_ = 23.4 Hz, Ph C-3,5), 118.7 (^1^*J*_C,F_ = 321.3 Hz, CF_3_), 121.7 (d, ^3^*J*_C,F_ = 8.7 Hz, Ph C-2,6), 131.1 (C-5), 134.6 (d, ^4^*J*_C,F_ = 3.1 Hz, NPh C-1), 152.4 (C-3), 162.5 (d, ^1^*J* = 249.8 Hz, Ph C-4), 181.0 (CHO). ^15^N NMR (41 MHz, CDCl_3_): *δ* −172.6 (N-1), N-2 was not found. MS *m*/*z* (%)*:* 339 ([M + H]^+^, 100). HRMS (ESI) for C_11_H_6_F_4_N_2_O_4_SNa ([M + Na]^+^): calcd 360.9877, found 360.9877.

#### 1-(3-Fluorophenyl)-4-formyl-1*H*-pyrazol-3-yl trifluoromethanesulfonate (5e)

White solid; yield 81% (274 mg); mp 81.0–84.0 °C. IR (KBr, *v*_max_, cm^−1^): 3130 (CH_arom_), 2862 (CH_aliph_), 1606, 1557, 1460, 1432, 1391, 1231, 1222, 1181, 1131 (C–F, C=C, C–N), 909, 854, 800, 787, 677, 657, 591, 513 (CH=CH of benzene). ^1^H NMR (700 MHz, CDCl_3_): *δ* 7.13–7.16 (m, 1H, Ph 4-H), 7.44–7.52 (m, 3H, Ph 2,5,6-H), 8.41 (s, 1H, 5-H), 9.92 (s, 1H, CHO). ^13^C NMR (176 MHz, CDCl_3_): *δ* 107.7 (d, ^2^*J*_C,F_ = 26.7 Hz, Ph C-2), 115.2 (C-4), 114.8 (d, ^4^*J*_C,F_ = 3.3 Hz, Ph C-6), 115.9 (d, ^2^*J*_C,F_ = 21.1 Hz, Ph C-4), 118.8 (q, ^1^*J*_C,F_ = 321.3 Hz, CF_3_), 131.2 (C-5), 131.5 (d, ^3^*J*_C,F_ = 9.0 Hz, Ph C-5), 139.5 (d, ^3^*J*_C,F_ = 10.1 Hz, Ph C-1), 152.5 (C-3), 163.3 (d, ^1^*J*_C,F_ = 249.5 Hz, Ph C-3), 181.1 (CHO). ^19^F NMR (376 MHz, CDCl_3_): *δ* −109.1. MS *m*/*z* (%): 339 ([M + H]^+^, 100). HRMS (ESI) for C_11_H_6_F_4_N_2_O_2_SNa ([M + Na]^+^): calcd 360.9877, found 360.9876.

#### 1-(2-Fluorophenyl)-4-formyl-1*H*-pyrazol-3-yl trifluoromethanesulfonate (5f)

White solid; yield 80% (270 mg); mp 85.0–88.0 °C. IR (KBr, *v*_max_, cm^−1^): 3148, 3099 (CH_arom_), 2856, 2824 (CH_aliph_), 1697, 1559, 1458, 1435, 1218, 1136 (C–F, C=C, C–N), 879, 821, 787, 761, 652, 607, 505 (CH=CH of benzene). ^1^H NMR (400 MHz, CDCl_3_): *δ* 7.26–7.35 (m, 2H, Ph 3,6-H), 7.39–7.45 (m, 1H, Ph 4-H), 7.83–7.87 (m, 1H, Ph 5-H), 8.49 (s, 1H, 5-H), 9.93 (s, 1H, CHO). ^13^C NMR (101 MHz, CDCl_3_): *δ* 114.8 (C-4), 117.4 (d, ^2^*J*_C,F_ = 20.1 Hz, Ph C-3), 118.8 (q, ^1^*J*_C,F_ = 321.4 Hz, CF_3_), 124.5 (C-5), 125.6 (d, ^4^*J*_C,F_ = 3.8 Hz Ph C-5), 126.5 (d, ^2^*J*_C,F_ = 9.1 Hz, Ph C-1), 130.2 (d, ^3^*J*_C,F_ = 8.0 Hz, Ph C-4), 135.9 (d, ^3^*J*_C,F_ = 11.6 Hz, C-4), 152.3 (C-3), 153.8 (d, ^1^*J*_C,F_ = 250.7 Hz, Ph C-2), 181.1 (CHO). ^19^F NMR (376 MHz, CDCl_3_): *δ* −125.0. MS *m*/*z* (%): 339 ([M + H]^+^, 100). HRMS (ESI) for C_11_H_6_F_4_N_2_O_2_SNa ([M + Na]^+^): calcd 360.9877, found 360.9880.

### General procedure for the synthesis of compounds 6a–h

To a solution of appropriate pyrazolyl trifluoromethanesulfonate 5a–f (0.5 mmol) in dry DMF (1 mL) under an inert atmosphere, TEA (4.0 mL, 2.5 mmol), appropriate pyridine acetylene (0.75 mmol), Pd(PPh_3_)_2_Cl_2_ (35 mg, 0.05 mmol), and CuI (18 mg, 0.1 mmol) were added. The reaction mixture was irradiated (100 W) at 130 °C for 1 h. After the completion of the reaction, as indicated by TLC, the mixture was quenched with water (10 mL) and extracted with ethyl acetate (3 × 10 mL). The organic layers were combined, washed with brine, dried over Na_2_SO_4_, and concentrated under reduced pressure. The obtained residue was purified by column chromatography (SiO_2_, eluent : ethyl acetate/*n*-hexane, 1 : 8, v/v) to yield compounds 6a–h.

#### 1-Phenyl-3-[(pyridin-4-yl)ethynyl]-1*H*-pyrazole-4-carbaldehyde (6a)

Brownish solid; yield 74% (101 mg); mp 134–135 °C. IR (KBr, *v*_max_, cm^−1^): 3118, 3086, 3045, 3025 (CH_arom_), 2226 (C≡C), 1680 (CHO), 1595, 1531, 1507, 1406, 1359, 1217 (C=C, C–N), 780, 748, 689, 681, 590 (CH=CH of benzene). ^1^H NMR (700 MHz, CDCl_3_): *δ* 7.42–7.44 (m, 1H, Ph 4-H), 7.48–7.49 (m, 2H, Pyr 3,5-H), 7.51–7.54 (m, 2H, Ph 3,5-H), 7.74–7.75 (m, 2H, Ph 2,6-H), 8.46 (s, 1H, 5-H), 8.67 (d, *J* = 5.5 Hz, 2H, Pyr 2,6-H), 10.01 (s, 1H, CHO). ^13^C NMR (176 MHz, CDCl_3_): *δ* 83.3 (Pyr-C≡C), 91.7 (Pyr-C≡C), 120.1 (Ph C-2,6), 125.8 (Pyr C-3,5), 130.1 (Pyr C-1), 126.3 (C-4), 128.8 (Ph C-4), 129.3 (C-5), 130.0 (Ph C-3,5), 137.2 (C-3), 138.8 (Ph C-4), 150.1 (Pyr C-2,6), 183.8 (CHO). ^15^N NMR (71 MHz, CDCl_3_): *δ* −156.1 (N-1), −67.5 (Pyr N-1), N-2 was not found. MS *m*/*z* (%): 274 ([M + H]^+^, 100). HRMS (ESI) for C_17_H_12_N_3_O ([M + H]^+^): calcd 274.0975, found 274.0975.

#### 1-Phenyl-3-[(pyridin-3-yl)ethynyl]-1*H*-pyrazole-4-carbaldehyde (6b)

Brownish solid; yield 70% (96 mg); mp 155–156 °C. IR (KBr, *v*_max_, cm^−1^): 3087, 3033 (CH_arom_), 2224 (C≡C), 1688 (C=O), 1596, 1566, 1527, 1500, 1412, 1360, 1300, 1236, 1226, 1159, 1022 (C=C, C–N), 957, 867, 810, 783, 768, 702, 695, 688, 619, 512 (CH=CH of benzene). ^1^H NMR (700 MHz, CDCl_3_): *δ* 7.38–7.40 (m, 1H, Pyr 5-H), 7.41–7.43 (m, 1H, Ph 4-H), 7.51–7.53 (m, 2H, Ph 3,5-H), 7.74–7.75 (m, 2H, Ph 2,6-H), 7.95–7.97 (m, 1H, Pyr 4-H), 8.46 (s, 1H, 5-H), 8.64 (br s, 1H, Pyr 6-H), 8.88 (br s, 1H, Pyr 2-H), 10.10 (s, 1H, CHO). ^13^C NMR (176 MHz, CDCl_3_): *δ* 82.8 (Pyr-C≡C), 90.9 (Pyr-C≡C), 119.6 (Pyr C-3), 120.1 (Ph C-2,6), 123.6 (Pyr C-5), 126.2 (C-4), 128.7 (Ph C-4), 129.3 (C-5), 129.9 (Ph C-3,5), 137.5 (C-3), 138.8 (Ph C-1), 139.7 (Pyr C-4), 148.9 (Pyr C-6), 151.9 (Pyr C-2), 183.8 (CHO). ^15^N NMR (71 MHz, CDCl_3_): *δ* −157.4 (N-1), −97.5 (N-2), −69.6 (Pyr N-1). MS *m*/*z* (%): 274 ([M + H]^+^, 100). HRMS (ESI) for C_17_H_12_N_3_O ([M + H]^+^): calcd 274.0975, found 274.0974.

#### 1-Phenyl-3-[(pyridin-2-yl)ethynyl]-1*H*-pyrazole-4-carbaldehyde (6c)

Brownish solid; yield 73% (100 mg); mp 114–115 °C. IR (KBr, *v*_max_, cm^−1^): 3128, 3063, 3045, 3000 (CH_arom_), 2228 (C≡C), 1682 (C=O), 1598, 1581, 1562, 1530, 1505, 1486, 1465, 1425, 1363, 1318, 1285, 1221, 1150, 1074, 1053 (C=C, C–N), 864, 780, 761, 755, 703, 686, 621, 525, 509, 438 (CH=CH of benzene). ^1^H NMR (700 MHz, CDCl_3_): *δ* 7.32–7.33 (m, 1H, Pyr 5-H), 7.41–7.43 (m, 1H, Ph 4-H), 7.51–7.53 (m, 2H, Ph 3,5-H), 7.64–7.65 (m, 1H, Pyr 3-H), 7.73–7.76 (m, 3H, Ph 2,6-H, Pyr 4-H), 8.46 (s, 1H, 5-H), 8.63–8.74 (m, 1H, Pyr 6-H), 10.15 (s, 1H, CHO). ^13^C NMR (176 MHz, CDCl_3_): *δ* 78.5 (Pyr-C≡C), 93.6 (Pyr-C≡C), 120.1 (Ph C-2,6), 123.9 (Pyr C-5), 126.5 (C-4), 127.9 (Pyr C-3), 128.6 (C-5), 128.7 (Ph C-4), 129.9 (Ph C-3,5), 136.4 (Pyr C-4), 137.8 (C-3), 138.9 (Ph C-1), 142.4 (Pyr C-2), 150.5 (Pyr C-6), 184.4 (CHO). ^15^N NMR (71 MHz, CDCl_3_): *δ* −157.2 (N-1), −65.8 (Pyr N-1), N-2 was not found. MS *m*/*z* (%): 274 ([M + H]^+^, 100). HRMS (ESI) for C_17_H_12_N_3_O ([M + H]^+^): calcd 274.0975, found 274.0975.

#### 1-(4-Methylphenyl)-3-[(pyridin-2-yl)ethynyl]-1*H*-pyrazole-4-carbaldehyde (6d)

Brownish solid; yield 85% (122 mg); mp 135.5–140.0 °C. IR (KBr, *v*_max_, cm^−1^): 3121, 3042 (CH_arom_), 2223 (C≡C), 1682 (C=O), 1581, 1535, 1522, 1485, 1426, 1362, 1271, 1234, 1223, 1155, 1052 (C=C, C–N, C–F), 959, 866, 813, 784, 772, 703, 628, 612, 506 (CH=CH of benzene). ^1^H NMR (700 MHz, CDCl_3_): *δ* 2.16 (s, 3H, CH_3_), 7.28–7.32 (m, 3H, Pyr 5-H, Ph 3,5-H), 7.60–7.65 (m, 3H, Pyr 3-H, Ph 2,6-H), 7.71–7.74 (m, 1H, Pyr 4-H), 8.40 (s, 1H, 5-H), 8.65–8.69 (m, 1H, Pyr 6-H), 10.12 (s, 1H, CHO). ^13^C NMR (176 MHz, CDCl_3_): *δ* 21.2 (CH_3_), 78.0 (Pyr-C≡C), 93.5 (Pyr-C≡C), 119.9 (Ph C-2,6), 123.8 (Pyr C-5), 126.3 (C-4), 127.9 (Pyr C-3), 128.6 (C-5), 130.4 (Ph C-3,5), 136.4 (Pyr C-4), 136.6 (Ph C-4), 137.5 (C-3), 138.7 (Ph C-1), 142.4 (Pyr C-2), 150.4 (Pyr C-6), 184.4 (CHO). MS *m*/*z* (%): 288 ([M + H]^+^, 100). HRMS (ESI) for C_18_H_14_N_3_O ([M + H]^+^): calcd 288.1131, found 288.1125.

#### 1-(4-Methoxyphenyl)-3-[(pyridin-2-yl)ethynyl]-1*H*-pyrazole-4-carbaldehyde (6e)

Brown solid; yield 86% (130 mg); mp 150.8–153.6 °C. IR (KBr, *v*_max_, cm^−1^): 3124 (CH_arom_), 2222 (C≡C), 1683 (C=O), 1533, 1518, 1487, 1455, 1437, 1257, 1219, 1173, 1025 (C=C, C–N), 959, 825, 777, 758, 721, 696, 617, 541, 520, 442 (CH=CH of benzene). ^1^H NMR (700 MHz, CDCl_3_): *δ* 3.86 (s, 3H, CH_3_), 7.00–7.02 (m, 2H, Ph 3,5-H), 7.31–7.34 (m, 1H, Pyr 5-H), 7.63–7.67 (m, 3H, Pyr 3-H, Ph 2,6-H), 7.72–7.75 (m, 1H, Pyr 4-H), 8.36 (s, 1H, 5-H), 8.67–8.68 (m, 1H, Pyr 6-H), 10.13 (s, 1H, CHO). ^13^C NMR (176 MHz, CDCl_3_): *δ* 55.8 (CH_3_), 78.6 (Pyr-C≡C), 93.4 (Pyr-C≡C), 114.9 (Ph C-3,5), 121.6 (Ph C-2,6), 123.8 (Pyr C-5), 126.3 (C-4), 127.9 (Pyr C-3), 128.5 (C-5), 132.4 (Ph C-1), 136.4 (Pyr C-4), 137.5 (C-3), 142.4 (Pyr C-2), 150.4 (Pyr C-6), 159.8 (Ph C-4), 184.4 (CHO). MS *m*/*z* (%): 304 ([M + H]^+^, 100). HRMS (ESI) for C_13_H_13_N_3_O_2_Na ([M + Na]): calcd 326.0900, found 326.0904.

#### 1-(4-Fluorophenyl)-3-[(pyridin-2-yl)ethynyl]-1*H*-pyrazole-4-carbaldehyde (6f)

Brownish solid; yield 88% (128 mg); mp 115.5–118.5 °C. IR (KBr, *v*_max_, cm^−1^): 3120, 3083 (CH_arom_), 2230 (C≡C), 1683 (C=O), 1586, 1534, 1488, 1480, 1471, 1362, 1241, 1226, 1157, 1112, 1054 (C=C, C–N, C–F), 958, 931, 875, 830, 793, 774, 762, 747, 657, 513, 435 (CH=CH of benzene). ^1^H NMR (700 MHz, CDCl_3_): *δ* 7.19–7.22 (m, 2H, Ph 3,5-H), 7.31–7.34 (m, 1H, Pyr 5-H), 7.63–7.64 (m, 1H, Pyr 3-H), 7.71–7.75 (m, 3H, Ph 2,6-H, Pyr 4-H), 8.40 (s, 1H, 5-H), 8.66–8.69 (m, 1H, Pyr 6-H), 10.13 (s, 1H, CHO). ^13^C NMR (176 MHz, CDCl_3_): *δ* 78.3 (Pyr-C≡C), 93.7 (Pyr-C≡C), 116.9 (d, ^2^*J*_C,F_ = 23.3 Hz, Ph C-3,5), 122.0 (d, ^3^*J*_C,F_ = 8.6 Hz, Ph C-2,6), 123.9 (Pyr C-5), 126.5 (C-4), 127.9 (Pyr C-3), 128.8 (C-5), 135.2 (d, ^4^*J*_C,F_ = 3.0 Hz, Ph C-1), 136.5 (Pyr C-4), 137.8 (C-3), 142.3 (Pyr C-2), 150.5 (Pyr C-6), 162.4 (d, ^1^*J*_C,F_ = 249.2 Hz, Ph C-2), 184.4 (CHO). MS *m*/*z* (%): 292 ([M + H]^+^, 100). HRMS (ESI) for C_17_H_10_N_3_OFNa ([M + Na]^+^): calcd 314.0700, found 314.0699.

#### 1-(3-Fluorophenyl)-3-[(pyridin-2-yl)ethynyl]-1*H*-pyrazole-4-carbaldehyde (6g)

Brownish solid; yield 85% (124 mg); mp 98.2–101.4 °C. IR (KBr, *v*_max_, cm^−1^): 3426 (CH_arom_), 1613, 1605, 1583, 1564, 1538, 1505, 1483, 1428, 1363, 1228, 1175, 1158, 1046 (C=C, C–N, C–F), 898, 860, 775, 673, 611, 454 (CH=CH of benzenes). ^1^H NMR (700 MHz, CDCl_3_): *δ* 7.07–7.11 (m, 1H, Ph 4-H), 7.30–7.32 (m, 1H, Pyr 5-H), 7.50–7.55 (m, 3H, Ph 2,5,6-H), 7.62–7.64 (m, 1H, Pyr 3-H), 7.71–7.74 (m, 1H, Pyr 4-H), 8.46 (s, 1H, 5-H), 8.64–8.66 (m, 1H, Pyr 6-H) 10.12 (s, 1H, CHO). ^13^C NMR (176 MHz, CDCl_3_): *δ* 78.2 (Pyr-C≡C), 93.8 (Pyr-C≡C), 107.9 (d, ^2^*J*_C,F_ = 26.6 Hz, Ph C-2), 115.1 (d, ^4^*J*_C,F_ = 3.2 Hz, Ph C-6), 115.4 (d, ^2^*J*_C,F_ = 21.1 Hz, Ph C-4), 123.9 (Pyr C-5), 126.6 (C-4), 127.9 (Pyr C-3), 128.5 (C-5), 131.3 (d, ^3^*J*_C,F_ = 9.1 Hz, Ph C-5), 136.4 (Pyr C-4), 137.8 (C-3), 139.9 (d, ^3^*J*_C,F_ = 10.1 Hz, Ph C-1), 142.2 (Pyr C-2), 150.5 (Pyr C-6), 163.3 (d, ^1^*J*_C,F_ = 248.6 Hz, Ph C-3), 184.2 (CHO). MS *m*/*z* (%): 292 ([M + H]^+^, 100). HRMS (ESI) for C_17_H_10_N_3_OFNa ([M + Na]^+^): calcd 314.0700, found 314.0698.

#### 1-(2-Fluorophenyl)-3-[(pyridin-2-yl)ethynyl]-1*H*-pyrazole-4-carbaldehyde (6h)

Brownish solid; yield 80% (116 mg); mp 110.8–112.5 °C. IR (KBr, *v*_max_, cm^−1^): 3111 (CH_arom_), 1685, 1632, 1598, 1583, 1532, 1515, 1484, 1427, 1358, 1255, 1223, 1205, 1114, 1049 (C=C, C–N, C–F), 990, 960, 850, 817, 780, 773, 754, 720, 675, 634, 607 (CH=CH of benzene). ^1^H NMR (700 MHz, CDCl_3_): *δ* 7.28–7.34 (m, 3H, Ph 3,4,5-H), 7.39–7.41 (m, 1H, Pyr 5-H), 7.65 (d, *J* = 7.8 Hz, 1H, Pyr 3-H), 7.73–7.76 (m, 1H, Pyr 4-H), 7.95–7.98 (m, 1H, Ph 6-H), 8.55 (d, *J* = 2.1 Hz, 1H, 5-H), 8.68 (d, *J* = 4.3 Hz, 1H, Pyr 6-H), 10.15 (s, 1H, CHO). ^13^C NMR (176 MHz, CDCl_3_): *δ* 78.3 (Pyr-C≡C), 93.7 (Pyr-C≡C), 117.2 (d, ^2^*J*_C,F_ = 20.2 Hz, Ph C-3), 123.9 (Pyr C-5), 124.8 (Ph C-6), 125.4 (d, ^3^*J*_C,F_ = 3.8 Hz, Ph C-5), 126.3 (C-4), 127.0 (d, ^2^*J*_C,F_ = 9.1 Hz, Ph C-1), 127.9 (Pyr C-3), 129.8 (d, ^3^*J*_C,F_ = 8.0 Hz, Ph C-4), 133.1 (d, ^3^*J*_C,F_ = 11.1 Hz, C-5), 136.4 (Pyr C-4), 137.3 (C-3), 142.3 (Pyr C-2), 150.4 (Pyr C-6), 153.7 (d, ^1^*J*_C,F_ = 250.9 Hz, Ph C-2), 184.2 (CHO). MS *m*/*z* (%): 292 ([M + H]^+^, 100). HRMS (ESI) for C_17_H_10_N_3_OFNa ([M + Na]^+^): calcd 314.0700, found 314.0704.

### General procedure for the synthesis of compounds 7a–h

A solution of compound 6a–h (0.5 mmol) in dry ammonia and methanol (NH_3_/MeOH 2 M, 8 mL) was heated at 120 °C for 15 h in a steel reactor. The solvent was evaporated, and the crude was purified by flash chromatography (SiO_2_, eluent : ethyl acetate/*n*-hexane, 1 : 2, v/v) to yield compounds 7a–h.

#### 2-Phenyl-6-(pyridin-4-yl)-2*H*-pyrazolo[4,3-*c*]pyridine (7a)

Yellowish solid; yield 91% (124 mg); mp 188–189 °C. IR (KBr, *v*_max_, cm^−1^): 3138, 3064, 3033 (CH_arom_), 1616, 1592, 1532, 1502, 1498, 1458, 1404, 1375, 1235, 1262, 1198, 1039 (C=C, C–N), 925, 829, 756, 746, 691, 501, 432 (CH=CH of monosubstituted benzene). ^1^H NMR (700 MHz, CDCl_3_): *δ* 7.47–7.51 (m, 1H, Ph 4-H), 7.56–7.60 (m, 2H, Ph 3,5-H), 7.92–7.95 (m, 2H, Ph 2,6-H), 7.98–8.00 (m, 2H, Pyr 3,5-H), 8.14 (dd, ^5^*J* = 1.3, 0.9 Hz, 1H, 7-H), 8.63 (d, ^5^*J* = 0.9 Hz, 1H, 3-H), 8.71–8.76 (m, 2H, Pyr 2,6-H), 9.34 (d, *J* = 1.3 Hz, 1H, 4-H). ^13^C NMR (176 MHz, CDCl_3_): *δ* 107.6 (C-7), 119.4 (C-3a), 120.2 (Pyr C-3,5), 120.4 (Ph C-2,6), 121.1 (C-3), 128.1 (Ph C-4), 128.9 (Ph C-3,5), 138.8 (Ph C-1), 146.1 (Pyr C-4), 146.5 (C-4), 147.3 (C-6), 149.2 (Pyr C-2,6), 150.2 (C-7a). ^15^N NMR (71 MHz, CDCl_3_): *δ* −144.2 (N-2), −98.0 (N-1), −86.8 (N-5), −72.3 (Pyr N-1). MS *m*/*z* (%): 273 ([M + H]^+^, 100). HRMS (ESI) for C_17_H_12_N_4_ ([M + H]^+^): calcd 273.1135, found 273.1135.

#### 2-Phenyl-6-(pyridin-3-yl)-2*H*-pyrazolo[4,3-*c*]pyridine (7b)

Yellowish solid; yield 85% (116 mg); mp 112–113 °C. IR (KBr, *v*_max_, cm^−1^): 3186, 3055, 3033 (CH_arom_), 1625, 1595, 1507, 1462, 1375, 1285, 1195 (C=C, C–N), 810, 771, 751, 708, 695, 580 (CH=CH of benzene). ^1^H NMR (700 MHz, CDCl_3_): *δ* 7.40 (dd, *J* = 7.9, 4.7 Hz, 1H, Pyr 5-H), 7.45–7.48 (m, 1H, Ph 4-H), 7.53–7.57 (m, 2H, Ph 3,5-H), 7.90–7.93 (m, 2H, Ph 2,6-H), 8.03 (dd, ^5^*J* = 1.3, 0.9 Hz, 1H, 7-H), 8.37 (dt, *J* = 7.9, 1.9 Hz, 1H, Pyr 4-H), 8.60 (d, ^5^*J* = 0.9 Hz, 1H, 3-H), 8.61–8.66 (m, 1H, Pyr 6-H), 9.29–9.31 (m, 1H, Pyr 2-H), 9.31 (d, *J* = 1.3 Hz, 1H, 4-H). ^13^C NMR (176 MHz, CDCl_3_): *δ* 107.7 (C-7), 119.9 (C-3a), 121.2 (Ph C-2,6), 122.0 (C-3), 123.5 (Pyr C-5), 129.0 (Ph C-4), 129.8 (Ph C-3,5), 134.4 (Pyr C-4), 135.5 (Pyr C-3), 139.8 (Ph C-1), 147.6 (C-4), 148.3 (Pyr C-2), 148.4 (C-6), 149.3 (Pyr C-6), 151.3 (C-7a). ^15^N NMR (71 MHz, CDCl_3_): *δ* −145.0 (N-2), −98.9 (N-1), −86.6 (N-5), −70.7 (Pyr N-1). MS *m*/*z* (%): 273 ([M + H]^+^, 100). HRMS (ESI) for C_17_H_12_N_4_ ([M + H]^+^): calcd 273.1135, found 273.1134.

#### 2-Phenyl-6-(pyridin-2-yl)-2*H*-pyrazolo[4,3-*c*]pyridine (7c)

Yellowish solid; yield 90% (122 mg); mp 187–188 °C. IR (KBr, *v*_max_, cm^−1^): 3105, 3054, 3010 (CH_arom_), 1618, 1587, 1566, 1531, 1507, 1465, 1424, 1370, 1329, 1230, 1201, 1077, 1058, 1023 (C=C, C–N), 918, 871, 804, 788, 765, 754, 740, 690, 673, 652, 622, 553, 492, 432, 406 (CH=CH of benzene). ^1^H NMR (700 MHz, CDCl_3_): *δ* 7.27–7.31 (m, 1H, Pyr 5-H), 7.46–7.48 (m, 1H, Ph 4-H), 7.56–7.58 (m, 2H, Ph 3,5-H), 7.81–7.83 (m, 1H, Pyr 4-H), 7.94–7.95 (m, 2H, Ph 2,6-H), 8.42 (d, *J* = 7.7 Hz, 1H, Pyr 3-H), 8.61 (s, 1H, 3-H), 8.71–8.75 (m, 2H, 7-H, Pyr 6-H), 9.32 (s, 1H, 4-H). ^13^C NMR (176 MHz, CDCl_3_): *δ* 108.9 (C-7), 120.7 (C-3a), 121.5 (Pyr C-3), 121.6 (Ph C-2,6), 122.0 (C-3), 123.2 (Pyr C-5), 129.0 (Ph C-4), 129.9 (Ph C-3,5), 137.0 (Pyr C-4), 140.2 (Ph C-1), 146.9 (C-4), 149.6 (Pyr C-6), 150.2 (C-6), 151.8 (C-7a), 156.8 (Pyr C-2). ^15^N NMR (71 MHz, CDCl_3_): *δ* −144.7 (N-2), −97.2 (N-1), −88.5 (N-5), −77.4 (Pyr N-1). MS *m*/*z* (%): 273 ([M + H]^+^, 100). HRMS (ESI) for C_17_H_12_N_4_ ([M + H]^+^): calcd 273.1135, found 273.1135.

#### 2-(4-Methylphenyl)-6-(pyridin-2-yl)-2*H*-pyrazolo[4,3-*c*]pyridine (7d)

White solid; yield 95% (136 mg); mp 187–188 °C. IR (KBr, *v*_max_, cm^−1^): 3105, 3054, 3010 (CH_arom_), 1618, 1587, 1566, 1531, 1507, 1465, 1424, 1370, 1329, 1230, 1201, 1077, 1058, 1023 (C=C, C–N), 918, 871, 804, 788, 765, 754, 740, 690, 673, 652, 622, 553, 492, 432, 406 (CH=CH of disubstituted benzene). ^1^H NMR (400 MHz, CDCl_3_): *δ* 2.38 (s, 3H, CH_3_), 7.21–7.24 (m, 1H, Pyr 5-H), 7.28–7.30 (m, 2H, Ph 3,5-H), 7.74–7.76 (m, 3H, Ph 2,6-H, Pyr 4-H), 8.35 (d, 1H, *J* = 8.0 Hz, Pyr 3-H), 8.57 (s, 1H, 3-H), 8.65–8.67 (m, 2H, 7-H, Pyr 6-H), 9.25 (s, 1H, 4-H). ^13^C NMR (101 MHz, CDCl_3_): *δ* 21.2 (CH_3_), 108.7 (C-7), 120.5 (C-3a), 121.29 (Ph C-2,6), 121.32 (Pyr C-3), 121.8 (C-3), 123.1 (Pyr C-5), 130.3 (Ph C-3,5), 136.8 (Pyr C-4), 137.8 (Ph C-4), 139.1 (Ph C-1), 146.6 (C-4), 149.5 (Pyr C-6), 149.9 (C-6), 151.6 (C-7a), 156.7 (Pyr C-2). MS *m*/*z* (%): 287 ([M + H]^+^, 100). HRMS (ESI) for C_18_H_15_N_4_ ([M + H]^+^): calcd 287.1291, found 287.1295.

#### 2-(4-Methoxyphenyl)-6-(pyridin-2-yl)-2*H*-pyrazolo[4,3-*c*]pyridine (7e)

White solid; yield 92% (139 mg); mp 162–166 °C. IR (KBr, *v*_max_, cm^−1^): 3122, 3062 (CH_arom_), 1684, 1584, 1532, 1519, 1481, 1439, 1251, 1230, 1169, 1048, 1028 (C=C, C–N, C–O–C), 920, 832, 792, 748, 698, 634, 521 (CH=CH of benzene). ^1^H NMR (700 MHz, CDCl_3_): *δ* 3.90 (s, 3H, CH_3_), 7.06–7.08 (m, 2H, Ph 3,5-H), 7.27–7.32 (m, 1H, Pyr 5-H), 7.81–7.87 (m, 3H, Ph 2,6-H, Pyr 4-H), 8.39–8.44 (m, 1H, Pyr 3-H), 8.53 (s 1H, 3-H), 8.71–8.73 (m, 2H, 7-H, Pyr 6-H), 9.31 (s, 1H, 4-H). ^13^C NMR (176 MHz, CDCl_3_): *δ* 55.8 (CH_3_), 108.8 (C-7), 115.0 (Ph C-3,5), 120.7 (C-3a), 121.5 (Pyr C-3), 121.8 (C-3), 123.0 (Ph C-2,6), 123.2 (Pyr C-5), 133.7 (Ph C-1), 137.0 (Pyr C-4), 146.6 (C-4), 149.6 (Pyr C-6), 150.1 (C-6), 151.7 (C-7a), 157.0 (Pyr C-2), 160.1 (Ph C-4). ^15^N NMR (71 MHz, CDCl_3_): *δ* −144.8 (N-2), N-1, N-5, Pyr N-1 were not found. MS *m*/*z* (%): 303 ([M + H]^+^, 100). HRMS (ESI) for C_18_H_15_N_4_O ([M + H]^+^): calcd 303.1240, found 303.1242.

#### 2-(4-Fluorophenyl)-6-(pyridin-2-yl)-2*H*-pyrazolo[4,3-*c*]pyridine (7f)

White solid; yield 93% (135 mg); mp 220–222 °C. IR (KBr, *v*_max_, cm^−1^): 3099, 3049 (CH_arom_), 1621, 1609, 1585, 1480, 1426, 1334, 1234, 1213, 1155, 1090, 1043 (C=C, C–N, C–F), 930, 844, 810, 788, 757, 620 (CH=CH of benzene). ^1^H NMR (700 MHz, CDCl_3_): *δ* 7.25–7.28 (m, 2H, Ph 3,5-H), 7.30 (t, *J* = 6.1 Hz, 1H, Pyr 5-H), 7.84 (t, *J* = 7.8 Hz, 1H, Pyr 4-H), 7.91–7.95 (m, 2H, Ph 2,6-H), 8.43 (d, *J* = 7.8 Hz, 1H, Pyr 3-H), 8.56 (s, 1H, 3-H), 8.72 (s, 1H, 7-H), 8.73–8.76 (m, 1H, Pyr 6-H), 9.33 (s, 1H, 4-H). ^13^C NMR (176 MHz, CDCl_3_): *δ* 108.6 (C-7), 116.7 (d, ^2^*J*_C,F_ = 23.1 Hz, Ph C-3,5), 120.6 (C-3a), 121.4 (Pyr C-3), 121.9 (C-3), 123.2 (Pyr C-5), 123.3 (d, ^3^*J*_C,F_ = 8.6 Hz, Ph C-2,6), 136.3 (d, ^4^*J*_C,F_ = 2.3 Hz, Ph C-1), 136.9 (Pyr C-4), 146.7 (C-4), 149.5 (Pyr C-6), 150.1 (C-6), 151.7 (C-7a), 156.6 (Pyr C-2), 162.6 (d, ^1^*J*_C,F_ = 249.6 Hz, Ph C-4). ^15^N NMR (71 MHz, CDCl_3_): *δ* −146.9 (N-2), −96.7 (N-1), −87.7 (N-5), −77.2 (Pyr N-1). ^19^F NMR (376 MHz, CDCl_3_): *δ* −112.1. MS *m*/*z* (%): 291 ([M + H]^+^, 100). HRMS (ESI) for C_17_H_12_N_4_F ([M + H]^+^): calcd 291.1041, found 291.1038.

#### 2-(3-Fluorophenyl)-6-(pyridin-2-yl)-2*H*-pyrazolo[4,3-*c*]pyridine (7g)

White solid; yield 95% (138 mg); mp 165.4–169.8 °C. IR (KBr, *v*_max_, cm^−1^): 3101, 3056 (CH_arom_), 1614, 1602, 1587, 1535, 1504, 1479, 1438, 1427, 1375, 1333, 1192, 1183, 1120 (C=C, C–N, C–F), 934, 875, 793, 755, 721, 695, 542 (CH=CH of benzene). ^1^H NMR (700 MHz, CDCl_3_): *δ* 7.15–7.19 (m, 1H, Ph 4-H), 7.29–7.31 (m, 1H, Pyr 5-H), 7.51–7.56 (m, 1H, Ph 5-H), 7.72–7.76 (m, 2H, Ph 2,6-H), 7.80–7.84 (m, 1H, Pyr 4-H), 8.42 (d, *J* = 7.7 Hz, 1H, Pyr 3-H), 8.61 (s 1H, 3-H), 8.69–8.75 (m, 2H, 7-H, Pyr 6-H), 9.32 (s, 1H, 4-H). ^13^C NMR (176 MHz, CDCl_3_): *δ* 108.6 (C-7), 109.3 (d, ^2^*J*_C,F_ = 26.3 Hz, Ph C-2), 115.8 (d, ^2^*J*_C,F_ = 21.2 Hz, Ph C-4), 116.6 (d, ^4^*J*_C,F_ = 3.2 Hz, Ph C-6), 120.6 (C-3a), 121.4 (Pyr C-3), 121.9 (C-3), 123.2 (Pyr C-5), 131.1 (d, ^3^*J*_C,F_ = 8.9 Hz, Ph C-5), 136.9 (Pyr C-4), 141.3 (d, ^3^*J*_C,F_ = 10.0 Hz, Ph C-1), 146.9 (C-4), 149.5 (Pyr C-6), 150.3 (C-6), 151.7 (C-7a), 156.6 (Pyr C-2), 163.2 (d, ^1^*J*_C,F_ = 248.5 Hz, Ph C-3). ^15^N NMR (71 MHz, CDCl_3_): *δ* −147.6 (N-2), −96.9 (N-1), −86.8 (N-5), −77.0 (Pyr N-1). ^19^F NMR (376 MHz, CDCl_3_): *δ* −109.7. MS *m*/*z* (%): 291 ([M + H]^+^, 100). HRMS (ESI) for C_17_H_12_N_4_F ([M + H]^+^): calcd 291.1041, found 291.1043.

#### 2-(2-Fluorophenyl)-6-(pyridin-2-yl)-2*H*-pyrazolo[4,3-*c*]pyridine (7h)

White solid; yield 94% (136 mg); mp 158–162.1 °C. IR (KBr, *v*_max_, cm^−1^): 3082, 3057 (CH_arom_), 1620, 1588, 1513, 1498, 1477, 1470, 1425, 1374, 1336, 1260, 1228, 1215, 1200, 1111, 1055 (C=C, C–N, C–F), 930, 876, 831, 806, 793, 673, 434, 408 (CH=CH of benzene). ^1^H NMR (700 MHz, CDCl_3_): *δ* 7.27–7.37 (m, 3H, Pyr 5-H, Ph 3,5-H), 7.41–7.47 (m, 1H, Ph 4-H), 7.81–7.83 (m, 1H, Pyr 4-H), 8.13–8.16 (m, 1H, Ph 6-H), 8.42–8.44 (m, 1H, Pyr 3-H), 8.70–8.75 (m, 3H, 3,7-H, Pyr 6-H), 9.33 (s, 1H, 4-H). ^13^C NMR (176 MHz, CDCl_3_): *δ* 108.6 (C-7), 117.2 (d, ^2^*J*_C,F_ = 20.4 Hz, Ph C-3), 120.6 (C-3a), 121.5 (Pyr C-3), 123.3 (Pyr C-5), 125.4 (d, ^4^*J*_C,F_ = 3.8 Hz, Ph C-5), 126.3 (C-3), 126.33 (d, ^3^*J*_C,F_ = 11.0 Hz, Ph C-6), 128.6 (d, ^2^*J*_C,F_ = 12.3 Hz, Ph C-1), 130.1 (d, ^3^*J*_C,F_ = 8.0 Hz, Ph C-4), 137.0 (Pyr C-4), 147.2 (C-4), 149.5 (Pyr C-6), 150.3 (C-6), 151.0 (C-7a), 154.2 (d, ^1^*J*_C,F_ = 251.3 Hz, Ph C-2), 156.7 (Pyr C-2). ^15^N NMR (71 MHz, CDCl_3_): *δ* −157.4 (N-2), −95.3 (N-1), −87.8 (N-5), −77.4 (Pyr N-1). ^19^F NMR (376 MHz, CDCl_3_): *δ* −124.1. MS *m*/*z* (%): 291 ([M + H]^+^, 100). HRMS (ESI) for C_17_H_12_N_4_ ([M + H]^+^): calcd 273.1135, found 273.1135.

### Biology

#### Cell cultures and viability assays

Human cell lines were obtained from European Collection of Authenticated Cell Cultures (K562, CEM), American Type Culture Collection (BJ, MRC-5) or Cell Lines Service (MV4-11), and they were cultivated according to the provider's instructions. Briefly, the K562, MRC-5, and BJ cell lines were maintained in DMEM medium, and the CEM and MV4-11 cell lines were maintained in an RPMI-1640 medium. All media were supplemented with 10–20% fetal bovine serum, penicillin (100 U mL^−1^), and streptomycin (0.1 mg mL^−1^), and cells were cultivated at 37 °C in 5% CO_2_.

For the viability assays, cells were treated with the tested compounds for 72 h. After treatments, resazurin (Sigma-Aldrich) solution was added for 4 h, and fluorescence of resorufin, corresponding to live cells, was measured at 544 nm/590 nm (excitation/emission) using a Fluoroskan Ascent microplate reader (Labsystems). The GI_50_ value, the drug concentration lethal to 50% of the cells, was calculated from the dose–response curves that resulted from the assays.

#### Cell cycle analysis

Asynchronously growing K562 cells were treated with increasing concentrations of the test compound for 24 and 48 h. After the staining with propidium iodide, DNA content was analyzed by flow cytometry using a 488 nm laser (BD FACSVerse with software BD FACSuite™, version 1.0.6). Cell cycle distribution was analyzed using ModFit LT (Verity Software House).

#### Immunoblotting

Cellular lysates were separated on SDS-polyacrylamide gels and electroblotted onto nitrocellulose membranes. After blocking, the membranes were incubated with specific primary antibodies overnight, washed, and then incubated with peroxidase-conjugated secondary antibodies. Finally, peroxidase activity was detected with SuperSignal West Pico reagents (Thermo Scientific) using a CCD camera LAS-4000 (Fujifilm). Specific antibodies were purchased from Santa Cruz Biotechnology (p-CDK1 T161), Cell Signaling (p-Plk1 T210; p-Bcl-2 S70; PARP-1; c-MYC; peroxidase-labeled secondary antibodies), Millipore (p-histone H3 S10; p-H2AX S319) and Sigma-Aldrich (p-CDK1 T14/Y15; Bcl-2; α-tubulin).

#### Immunofluorescence

Cells were seeded on poly-l-lysine-coated cover slips and, after a preincubation period, treated with 7f. After the treatment, slides were fixed by ice-cold methanol : acetone (1 : 1). Then, slides were rehydrated with PBS, blocked in the solution of 0.5% BSA, and incubated with primary antibody against α-tubulin. Subsequently, cells were washed and incubated with secondary antibody conjugated with Alexa Fluor™ 488, and stained with DAPI. Observations were performed using a fluorescence microscope, Olympus IX51.

#### BrdU incorporation

K562 cells were treated with 7f for 24 and 48 h and 30 min before the end of incubation, the cells were labelled with 10 µM BrdU (Sigma-Aldrich). Subsequently, the cells were washed in PBS, fixed with ice-cold 70% ethanol, and denatured in 2 M HCl. After neutralization, the cells were stained with an anti-BrdU FITC-labelled antibody (eBioscience) and propidium iodide (Sigma-Aldrich). Samples were then analysed by flow cytometry using a 488 nm laser (BD FACSVerse with software BD FACSuite™, version 1.0.6.; BD, Franklin Lakes, NJ, USA).

## Conclusions

In this study, we successfully enhanced the antimitotic properties of 2*H*-pyrazolo[4,3-*c*]pyridines by systematically modifying the 2- and 6-positions of the system. Our efforts culminated in the identification of compound 7f, bearing 4-fluorophenyl and pyridin-2-yl substituents at the 2- and 6-positions, respectively, which demonstrated potent submicromolar cytotoxic activity against a panel of cancer cell lines. The biological evaluation revealed that compound 7f affects the integrity of microtubules, leading to the induction of mitotic defects, disruption of proper cytokinesis, and endoreduplication, resulting in cell death. The pronounced activity of 7f underscores the effectiveness of our chemical modifications and validates the approach of using bioisosteric replacements to optimize biological activity. These findings pave the way for further exploration of 2*H*-pyrazolo[4,3-*c*]pyridines as potential anti-cancer agents, with compound 7f serving as a possible lead for future drug development.

## Author contributions

Conceptualization, A. Š., A. Ž., V. K., and E. A.; investigation, V. A., E. Ř., A. Š.-N.; V. V., V. M., S. B., and A. B.; formal analysis, V. A., E. Ř., and A. Š.-N.; data curation, V. K., and E. A.; funding acquisition, A. Š., and V. K.; methodology, V. K., and E. A.; resources, A. Š., V. K., and E. A.; supervision, V. K., and E. A.; writing—original draft, E. Ř., A. B., S. B., A. Ž., and E. A.; writing—review and editing, A. Š., and V. K. All authors have read and agreed to the published version of the manuscript.

## Conflicts of interest

There are no conflicts of interest to declare.

## Supplementary Material

RA-016-D5RA09208F-s001

RA-016-D5RA09208F-s002

RA-016-D5RA09208F-s003

## Data Availability

The data supporting this article have been included as part of the supplementary information (SI). Supplementary information: ^1^H, ^13^C, ^1^H–^15^N HMBC, ^19^F NMR, HRMS data, BrdU incorporation-based cellular proliferation analysis and raw western blot data. See DOI: https://doi.org/10.1039/d5ra09208f. CCDC 2524805 contains the supplementary crystallographic data for this paper.^66^
