# Supplementary material for: Synthesis and antimitotic activity of 2-phenyl-6-pyridinyl-2H-pyrazolo[4,3-c]pyridines
Source: RSC Adv. 2026 May 12;16(27):24822–37. doi: 10.1039/d5ra09208f (PMC13161753; doi:10.1039/d5ra09208f)
Supplement: RA-016-D5RA09208F-s001 [file RA-016-D5RA09208F-s001.pdf]

Supplementary Information (SI) for RSC Advances.
This journal is © The Royal Society of Chemistry 2026

Supporting Information

Synthesis and antimitotic activity of 2-phenyl-6-pyridinyl-2*H*-pyrazolo[4,3-*c*]pyridines

Vaida Aleksienė,[†a] Eva Řezníčková,[†b] Aurimas Bieliauskas,[a] Veronika Vojáčková,[b] Veronika Molitorová,[b] Austėja Šalvytė-Nikliauzienė,[c] Sergey Belyakov,[d] Asta Žukauskaitė,[e] Eglė Arbačiauskienė,[c] Vladimír Kryštof*[b,f] and Algirdas Šačkus*[a,c]

[a] Institute of Synthetic Chemistry, Faculty of Chemical Technology, Kaunas University of Technology, K. Baršausko g. 59, LT-51423 Kaunas, Lithuania. E-mail: algirdas.sackus@ktu.lt

[b] Department of Experimental Biology, Faculty of Science, Palacký University, Šlechtitelů 27, CZ-77900 Olomouc, Czech Republic. E-mail: vladimir.krystof@upol.cz

[c] Department of Organic Chemistry, Faculty of Chemical Technology, Kaunas University of Technology, Radvilėnų pl. 19, LT-50254 Kaunas, Lithuania

[d] Latvian Institute of Organic Synthesis, Aizkraukles 21, LV-1006 Riga, Latvia

[e] Department of Chemical Biology, Faculty of Science, Palacký University, Šlechtitelů 27, CZ-77900 Olomouc, Czech Republic

[f] Institute of Molecular and Translational Medicine, Faculty of Medicine and Dentistry, Palacký University, Hněvotínská 5, CZ-77900 Olomouc, Czech Republic

† Vaida Aleksienė and Eva Řezníčková contributed equally to this work

* Correspondence:

Algirdas Šačkus
algirdas.sackus@ktu.lt

Vladimír Kryštof
vladimir.krystof@upol.cz

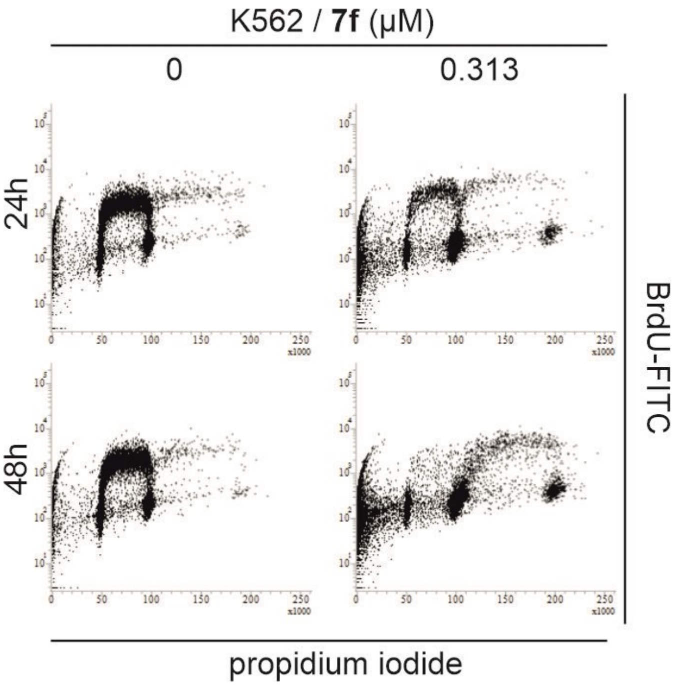

Figure S1. Analysis of cellular proliferation using BrdU incorporation in K562 cells treated with **7f** for 24 and 48 h

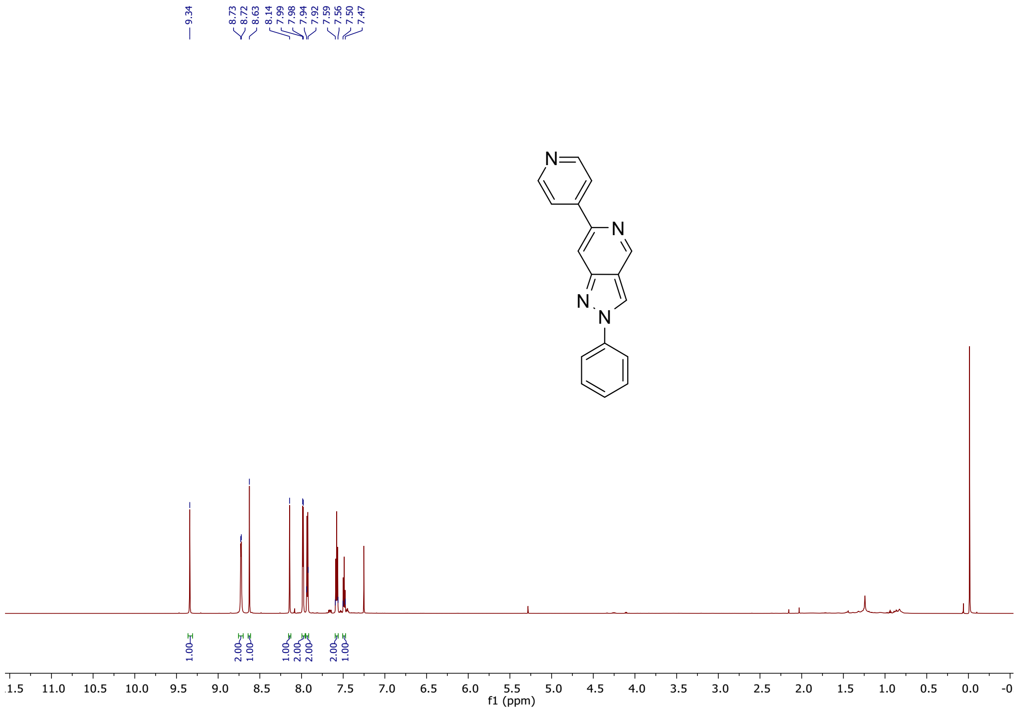

Figure S2. ^{1}H NMR spectrum (700 MHz, $CDCl_3$) of 2-phenyl-6-(pyridin-4-yl)-2*H*-pyrazolo[4,3-*c*]pyridine (**7a**)

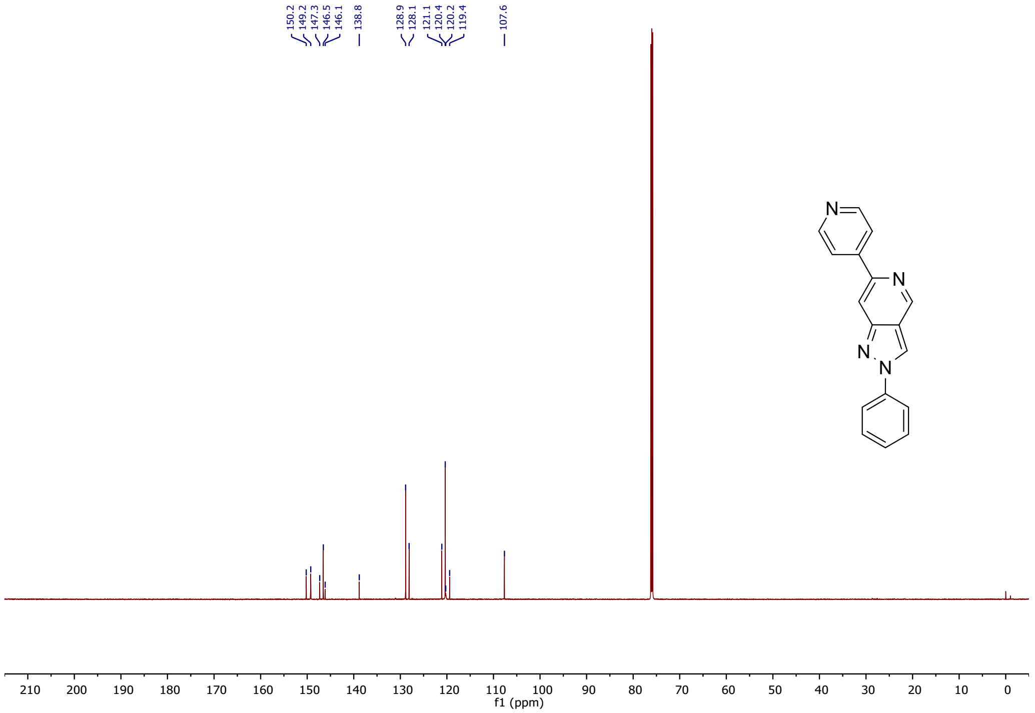

Figure S3. ^{13}C NMR spectrum (176 MHz, $CDCl_3$) of 2-phenyl-6-(pyridin-4-yl)-2*H*-pyrazolo[4,3-*c*]pyridine (**7a**)

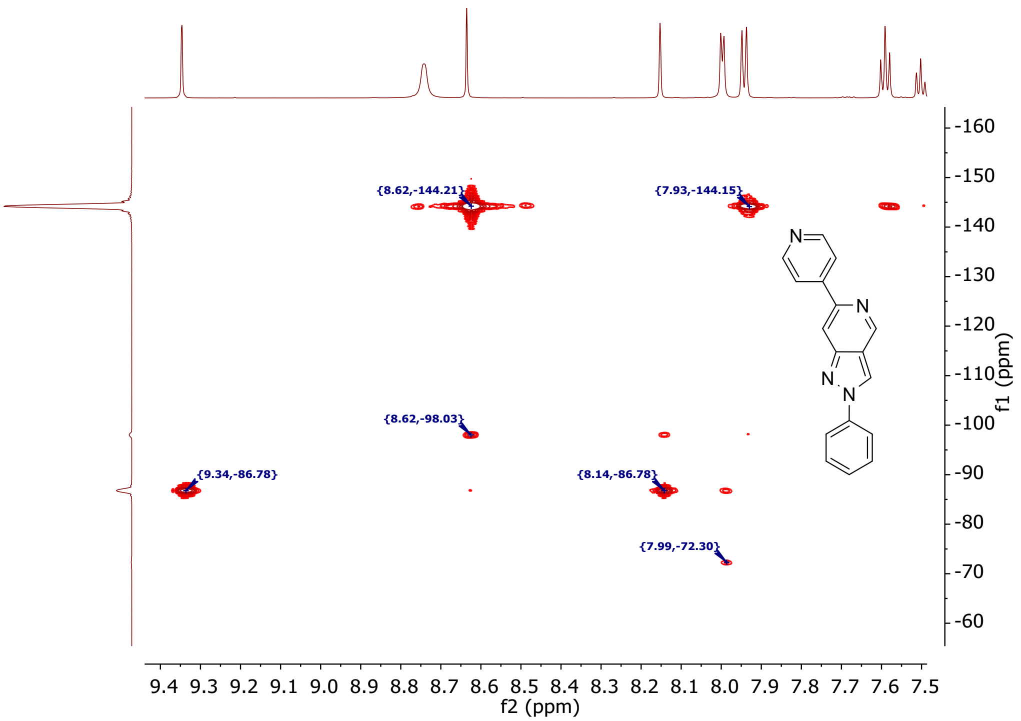

Figure S4. ^{1}H-^{15}N HMBC NMR spectrum (71 MHz, $CDCl_3$) of 2-phenyl-6-(pyridin-4-yl)-2*H*-pyrazolo[4,3-*c*]pyridine (**7a**)

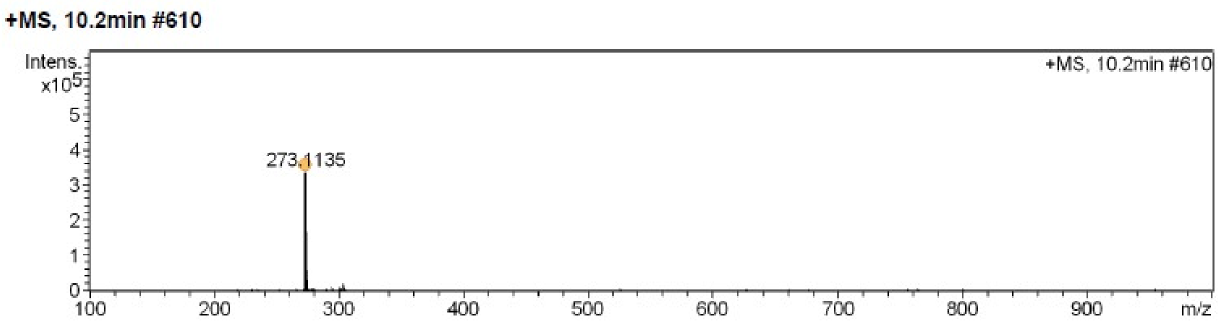

Meas. m/z	#	Ion Formula	m/z	err [ppm]	mSigma	# Sigma	Score	rdb	e⁻ Conf	N-Rule
273.1135	1	C17H13N4	273.1135	-0.1	8.7	1	100.00	13.5	even	ok

Figure S5. HRMS (ESI) report of **7a**

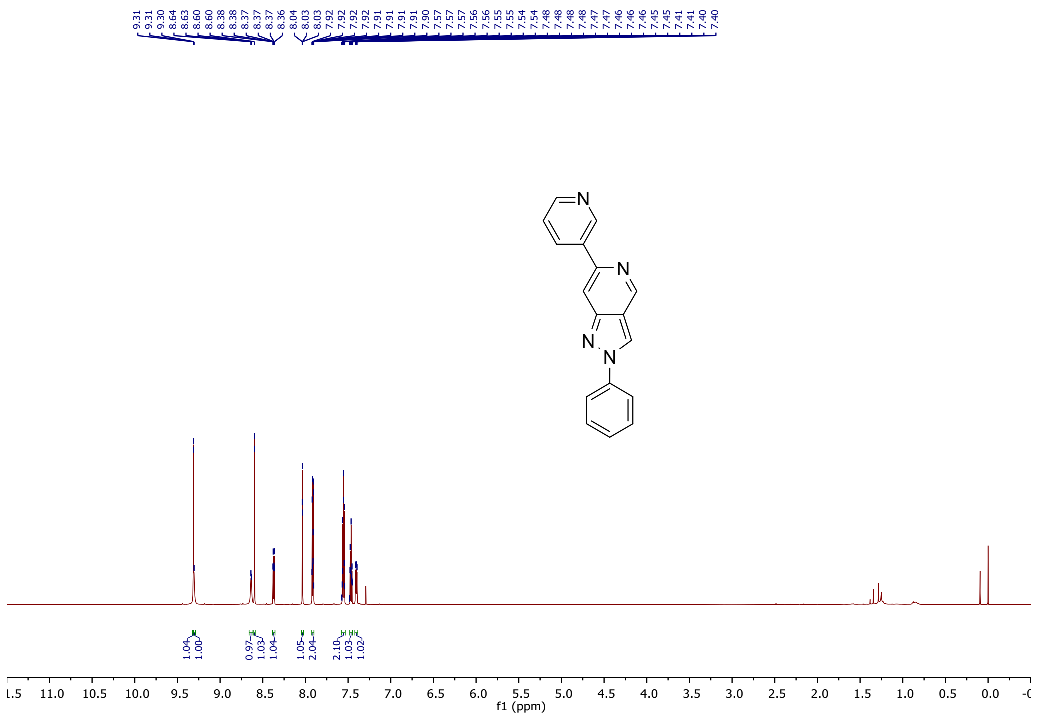

Figure S6. ^{1}H NMR spectrum (700 MHz, $CDCl_3$) of 2-phenyl-6-(pyridin-3-yl)-2*H*-pyrazolo[4,3-*c*]pyridine (**7b**)

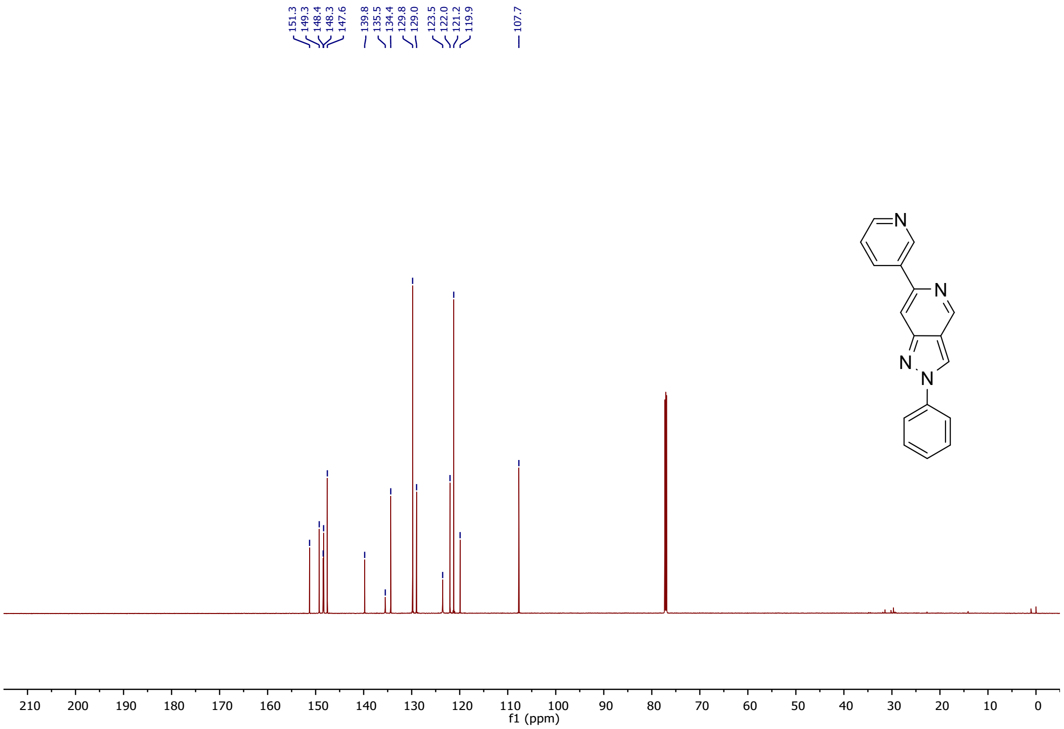

Figure S7. ^{13}C NMR spectrum (176 MHz, $CDCl_3$) of 2-phenyl-6-(pyridin-3-yl)-2*H*-pyrazolo[4,3-*c*]pyridine (**7b**)

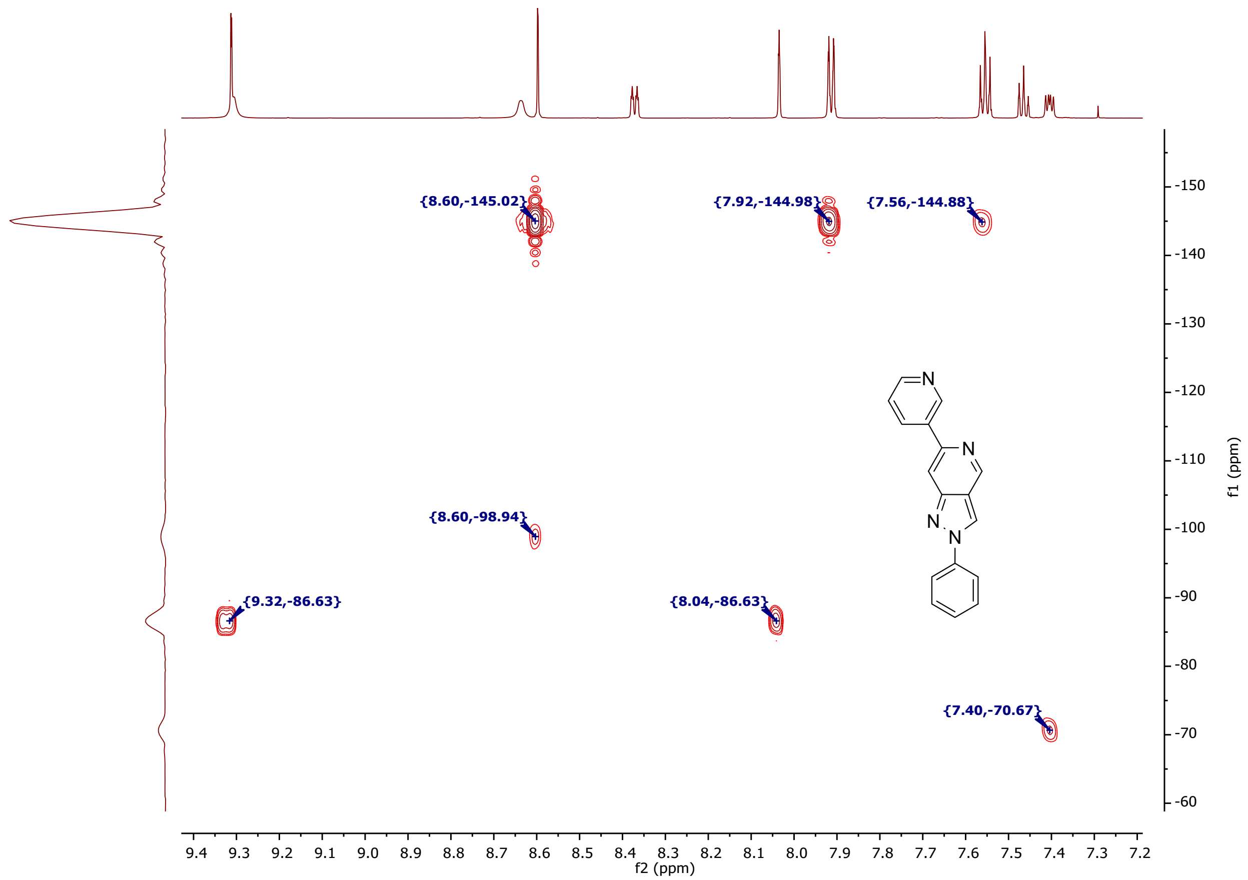

Figure S8. ^{1}H-^{15}N HMBC NMR spectrum (71 MHz, $CDCl_3$) of 2-phenyl-6-(pyridin-3-yl)-2*H*-pyrazolo[4,3-*c*]pyridine (**7b**)

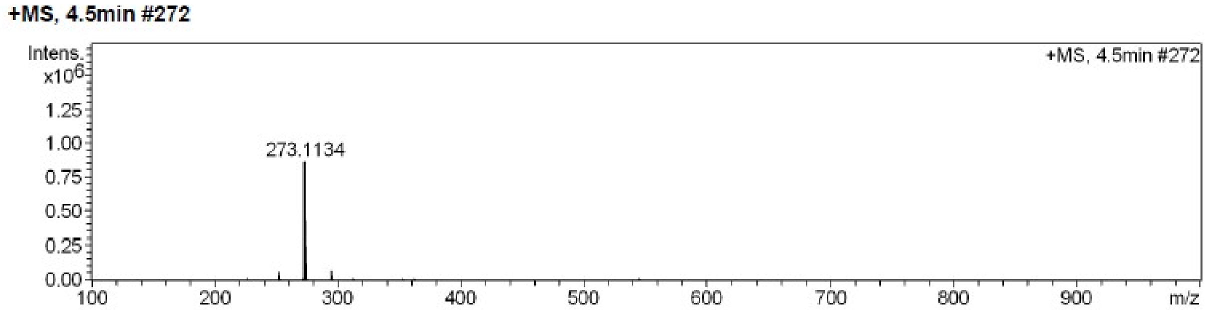

Meas. m/z	#	Ion Formula	m/z	err [ppm]	mSigma	# Sigma	Score	rdb	e¯ Conf	N-Rule
273.1134	1	C17H13N4	273.1135	0.4	52.7	1	100.00	13.5	even	ok

Figure S9. HRMS (ESI) report of **7b**

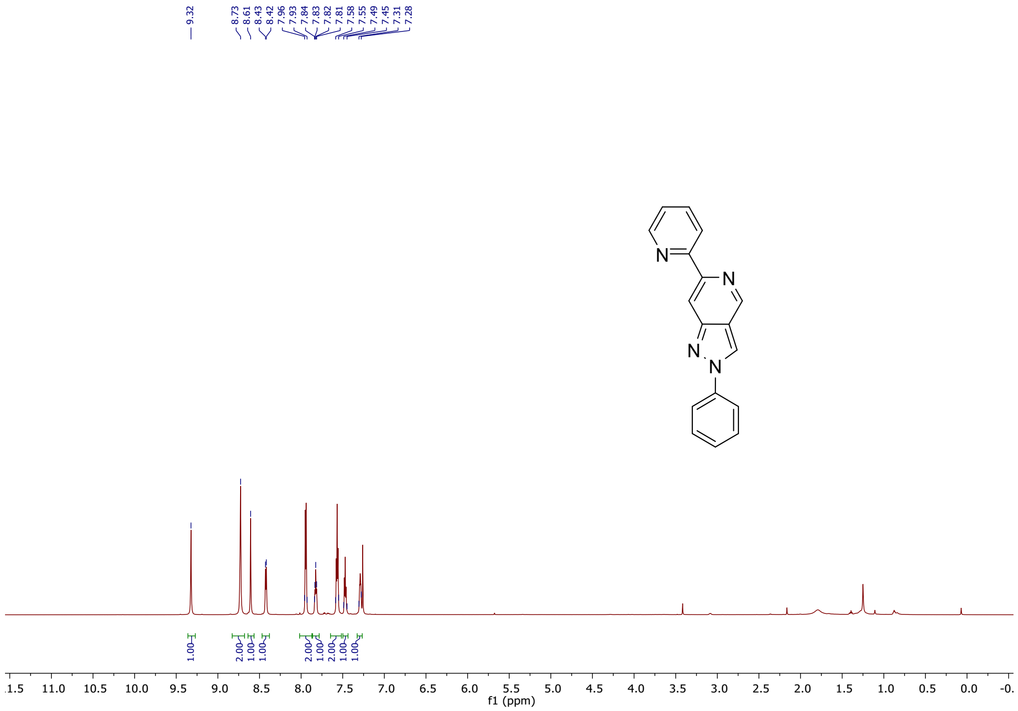

Figure S10. ^{1}H NMR spectrum (700 MHz, $CDCl_3$) of 2-phenyl-6-(pyridin-2-yl)-2*H*-pyrazolo[4,3-*c*]pyridine (**7c**)

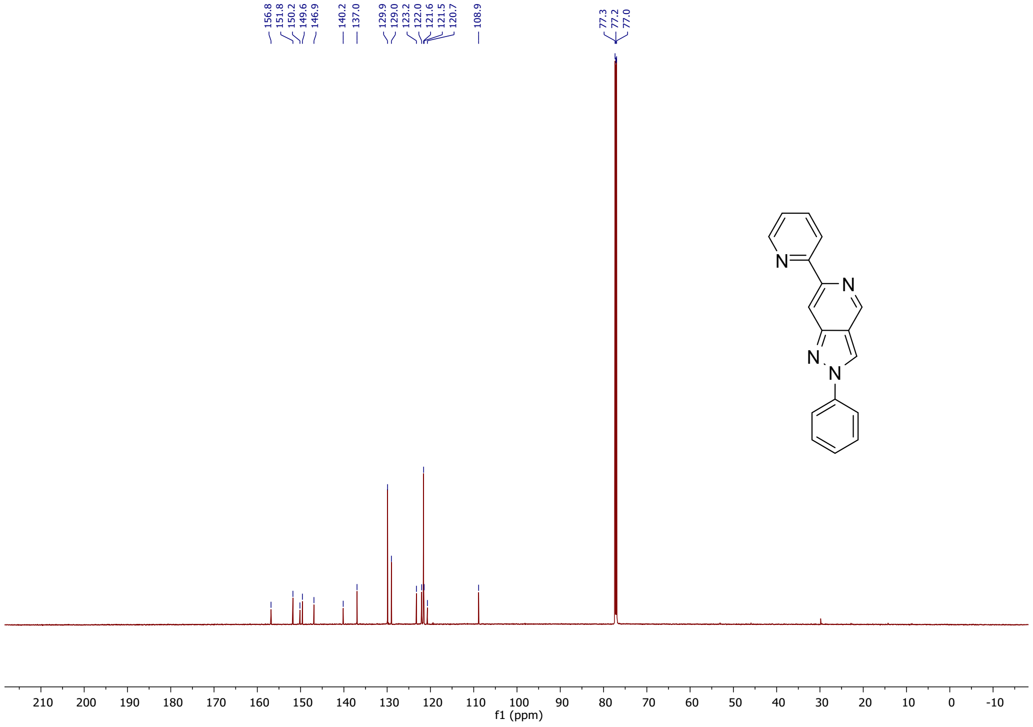

Figure S11. ^{13}C NMR spectrum (176 MHz, $CDCl_3$) of 2-phenyl-6-(pyridin-2-yl)-2*H*-pyrazolo[4,3-*c*]pyridine (**7c**)

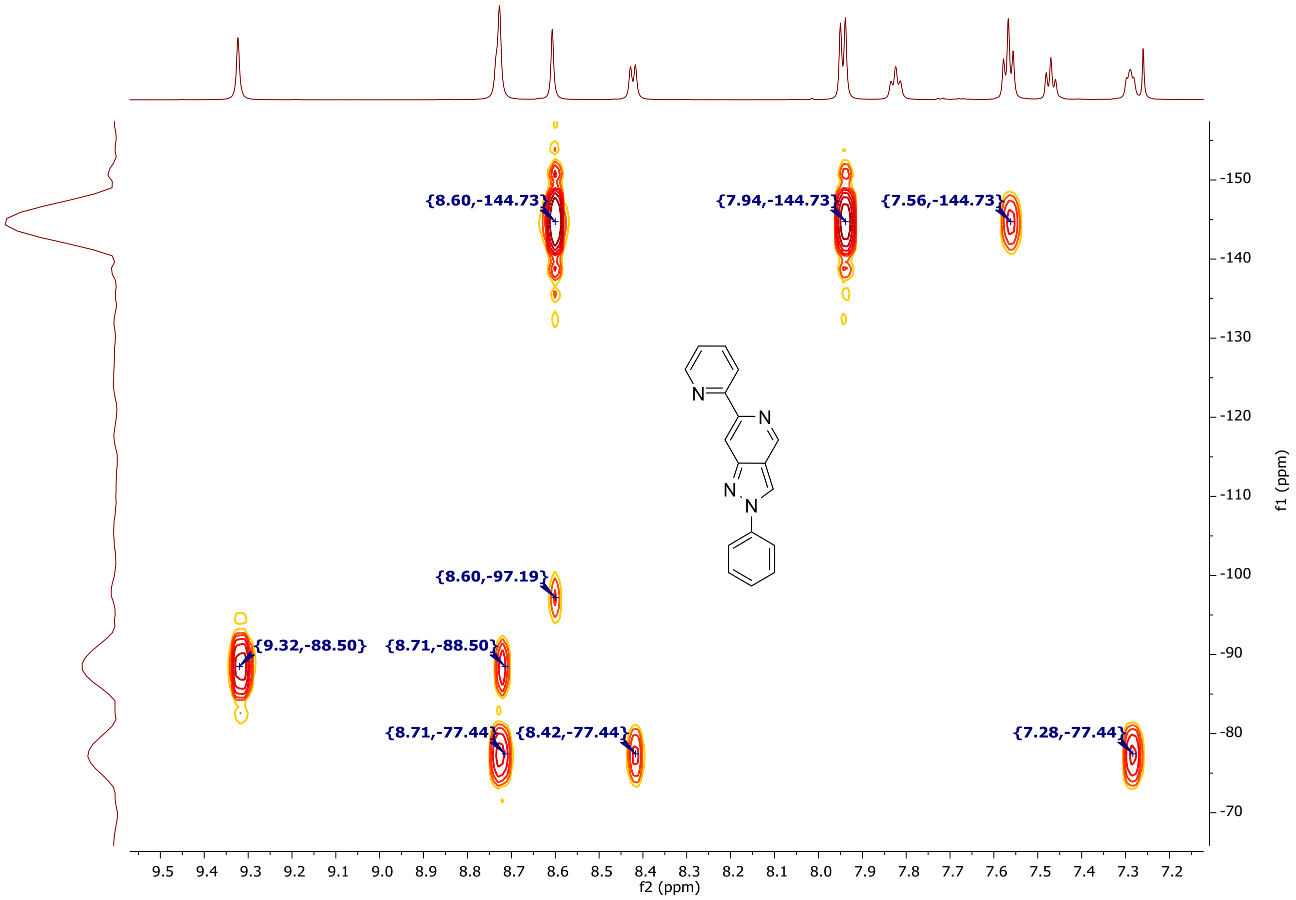

Figure S12. ^{1}H-^{15}N HMBC NMR spectrum (71 MHz, $CDCl_3$) of 2-phenyl-6-(pyridin-2-yl)-2*H*-pyrazolo[4,3-*c*]pyridine (**7c**)

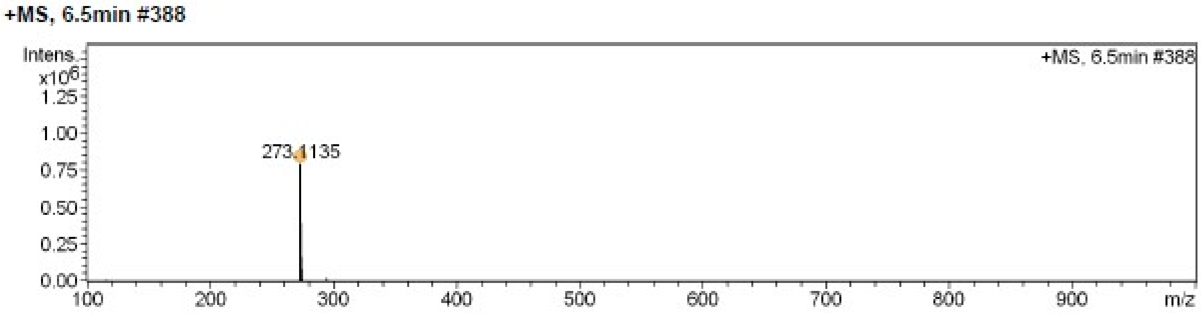

Meas. m/z	#	Ion Formula	m/z	err [ppm]	mSigma	# Sigma	Score	rdb	e⁻ Conf	N-Rule
273.1135	1	C17H13N4	273.1135	-0.1	8.0	1	100.00	13.5	even	ok

Figure S13. HRMS (ESI) report of **7c**

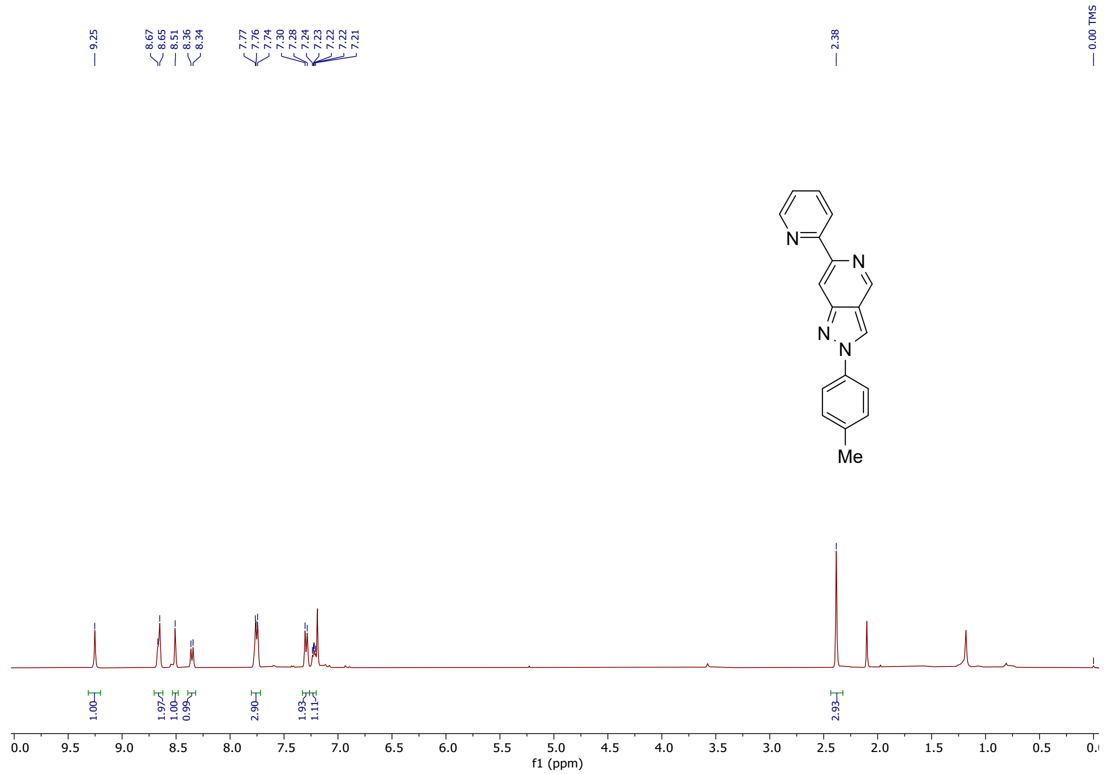

Figure S14. ^{1}H NMR spectrum (400 MHz, $CDCl_3$) of 2-(4-methylphenyl)-6-(pyridin-2-yl)-2*H*-pyrazolo[4,3-*c*]pyridine (**7d**)

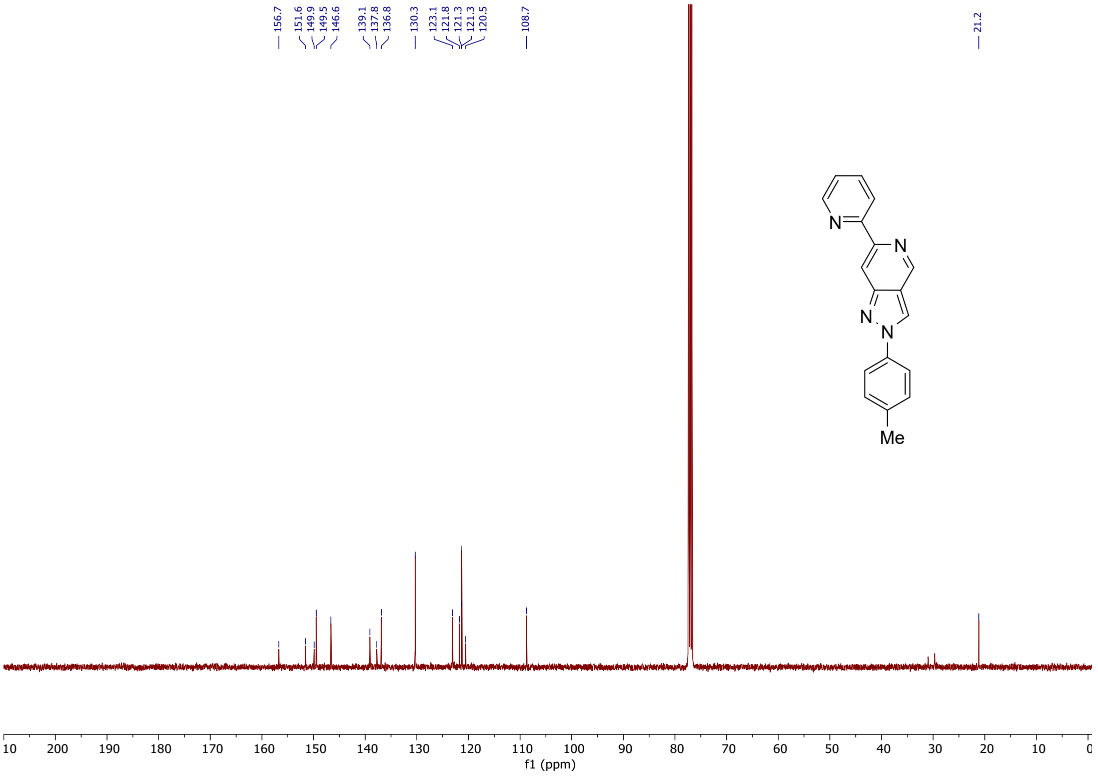

Figure S15. ^{13}C NMR spectrum (101 MHz, $CDCl_3$) of 2-(4-methylphenyl)-6-(pyridin-2-yl)-2*H*-pyrazolo[4,3-*c*]pyridine (**7d**)

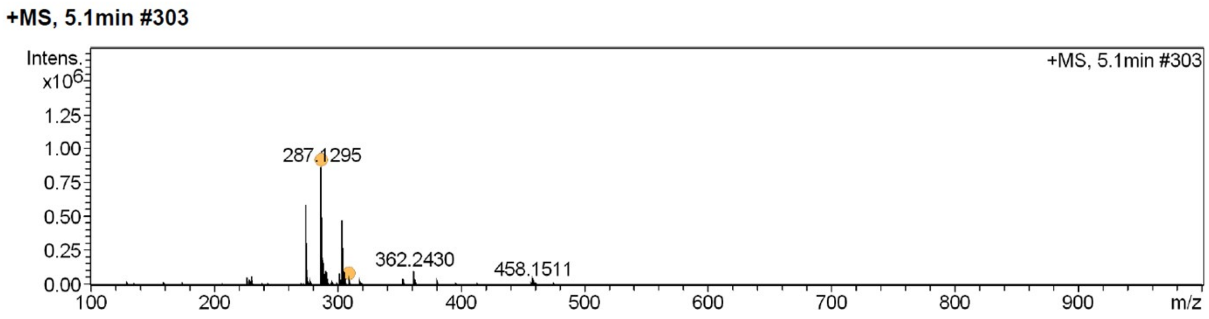

Meas. m/z	#	Ion Formula	m/z	err [ppm]	mSigma	# Sigma	Score	rdb	e⁻ Conf	N-Rule
287.1295	1	C18H15N4	287.1291	1.3	12.7	1	100.00	13.5	even	ok
309.1102	1	C18H14N4Na	309.1111	-2.9	30.1	1	100.00	13.5	even	ok

Figure S16. HRMS (ESI) report of **7d**

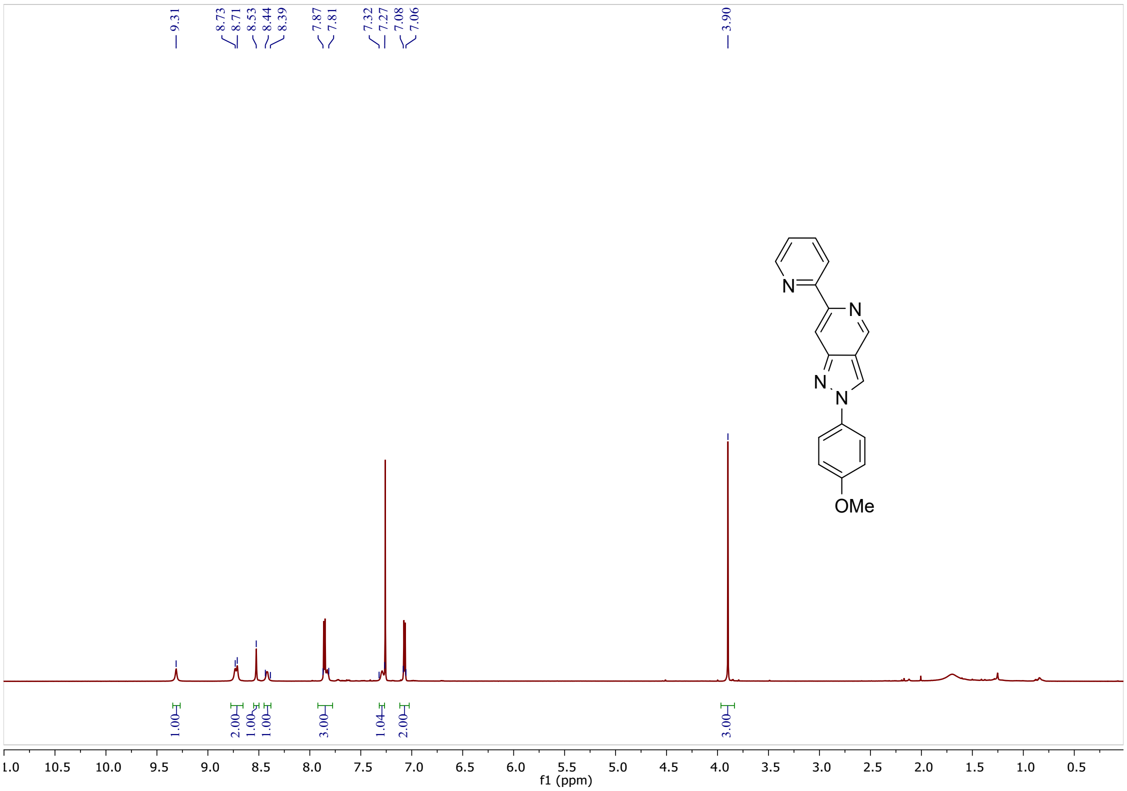

Figure S17. ^{1}H NMR spectrum (700 MHz, $CDCl_3$) of 2-(4-methoxyphenyl)-6-(pyridin-2-yl)-2*H*-pyrazolo[4,3-*c*]pyridine (**7e**)

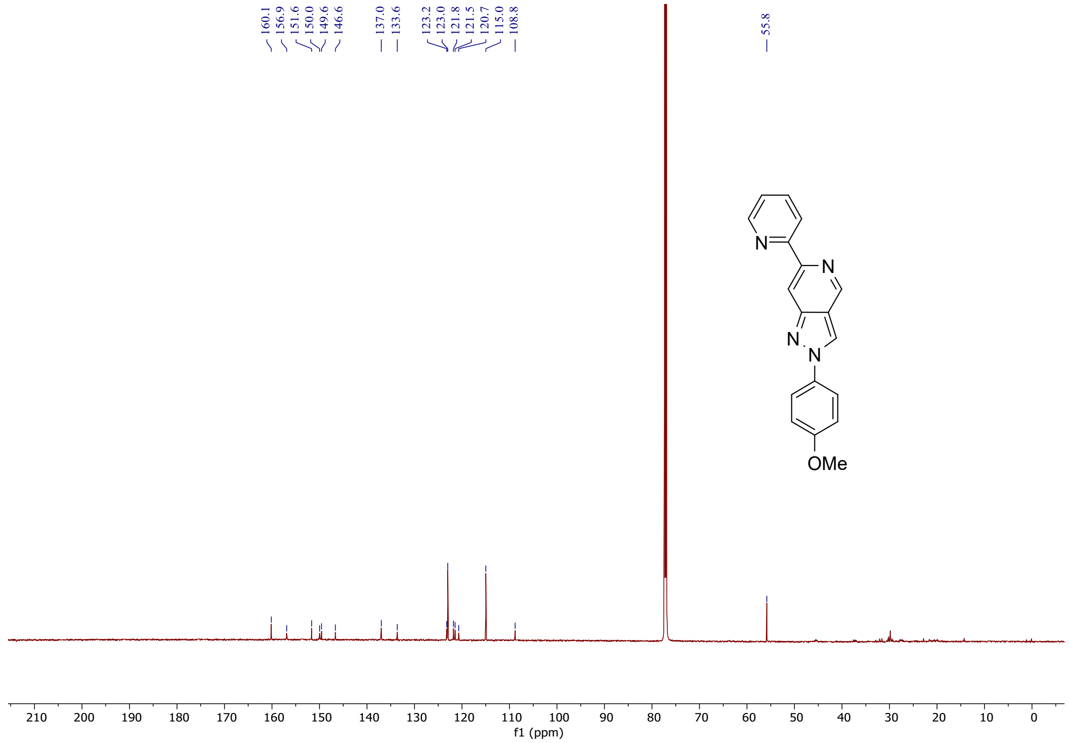

Figure S18. ^{13}C NMR spectrum (176 MHz, $CDCl_3$) of 2-(4-methoxyphenyl)-6-(pyridin-2-yl)-2*H*-pyrazolo[4,3-*c*]pyridine (**7e**)

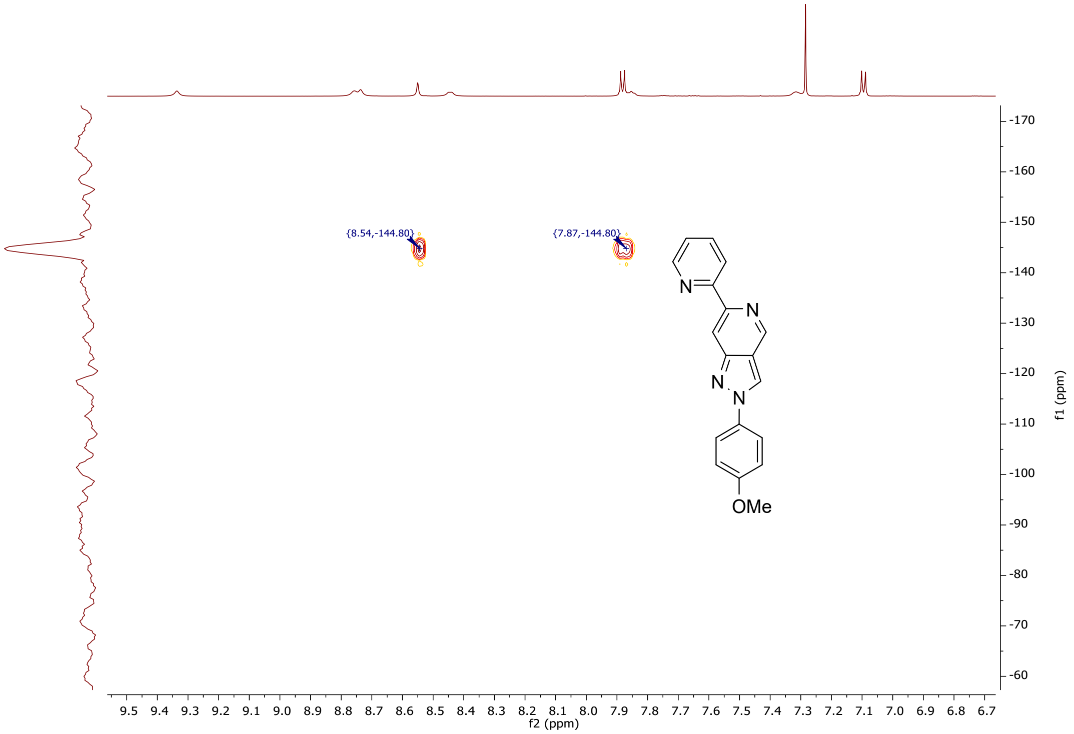

Figure S19. ^{1}H-^{15}N HMBC NMR spectrum (71 MHz, $CDCl_3$) of 2-(4-methoxyphenyl)-6-(pyridin-2-yl)-2*H*-pyrazolo[4,3-*c*]pyridine (**7e**)

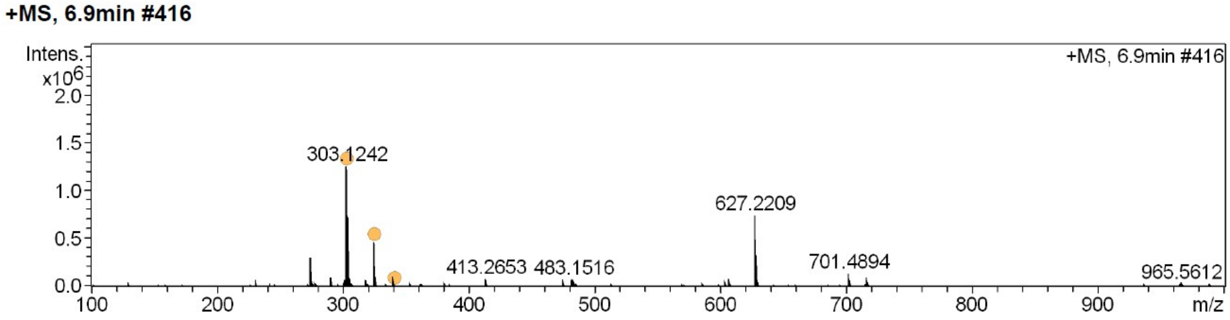

Meas. m/z	#	Ion Formula	m/z	err [ppm]	mSigma	# Sigma	Score	rdb	e⁻ Conf	N-Rule
303.1242	1	C18H15N4O	303.1240	0.4	217.5	1	100.00	13.5	even	ok
325.1057	1	C18H14N4NaO	325.1060	0.8	2.1	1	100.00	13.5	even	ok
341.0822	1	C18H14KN4O	341.0799	-6.7	26.4	1	100.00	13.5	even	ok

Figure S20. HRMS (ESI) report of **7e**

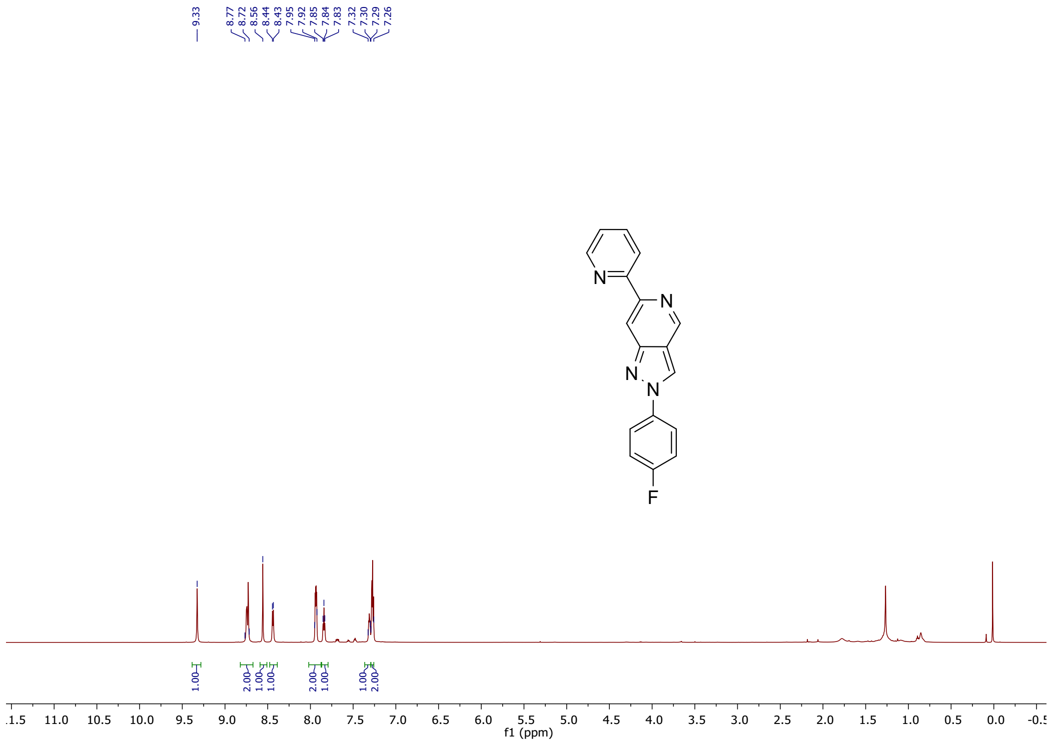

Figure S21. ^{1}H NMR spectrum (700 MHz, $CDCl_3$) of 2-(4-fluorophenyl)-6-(pyridin-2-yl)-2*H*-pyrazolo[4,3-*c*]pyridine (**7f**)

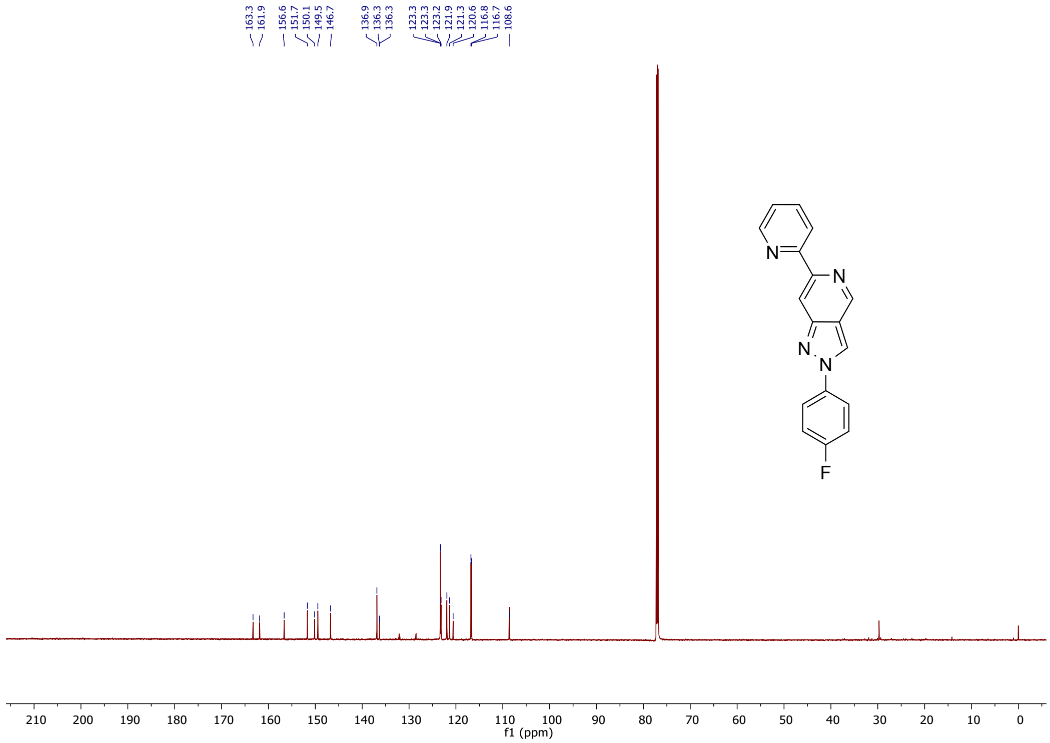

Figure S22. ^{13}C NMR spectrum (176 MHz, $CDCl_3$) of 2-(4-fluorophenyl)-6-(pyridin-2-yl)-2*H*-pyrazolo[4,3-*c*]pyridine (**7f**)

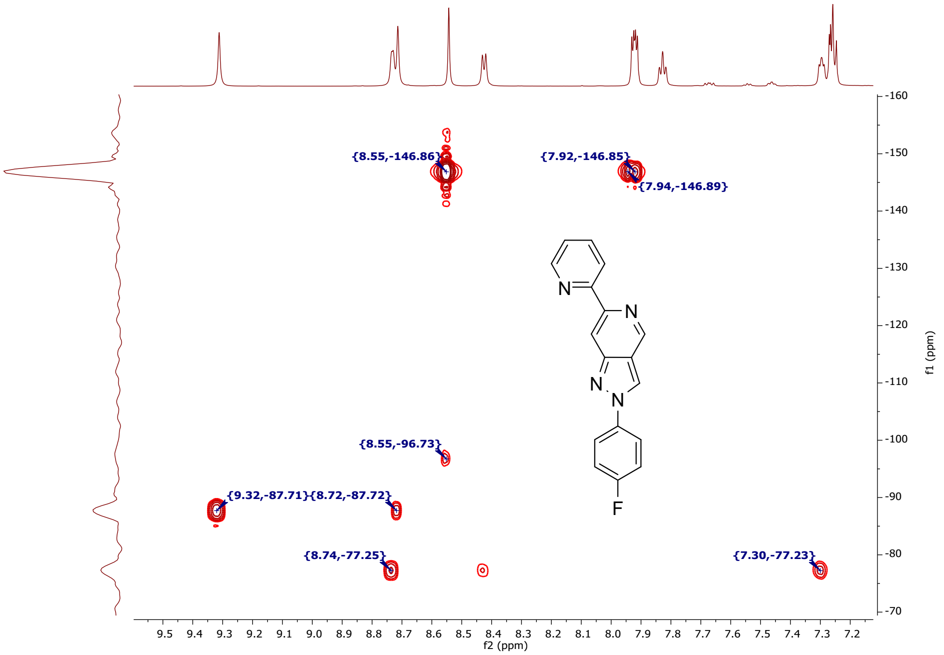

Figure S23. ^{1}H-^{15}N HMBC NMR spectrum (71 MHz, $CDCl_3$) of 2-(4-fluorophenyl)-6-(pyridin-2-yl)-2*H*-pyrazolo[4,3-*c*]pyridine (**7f**)

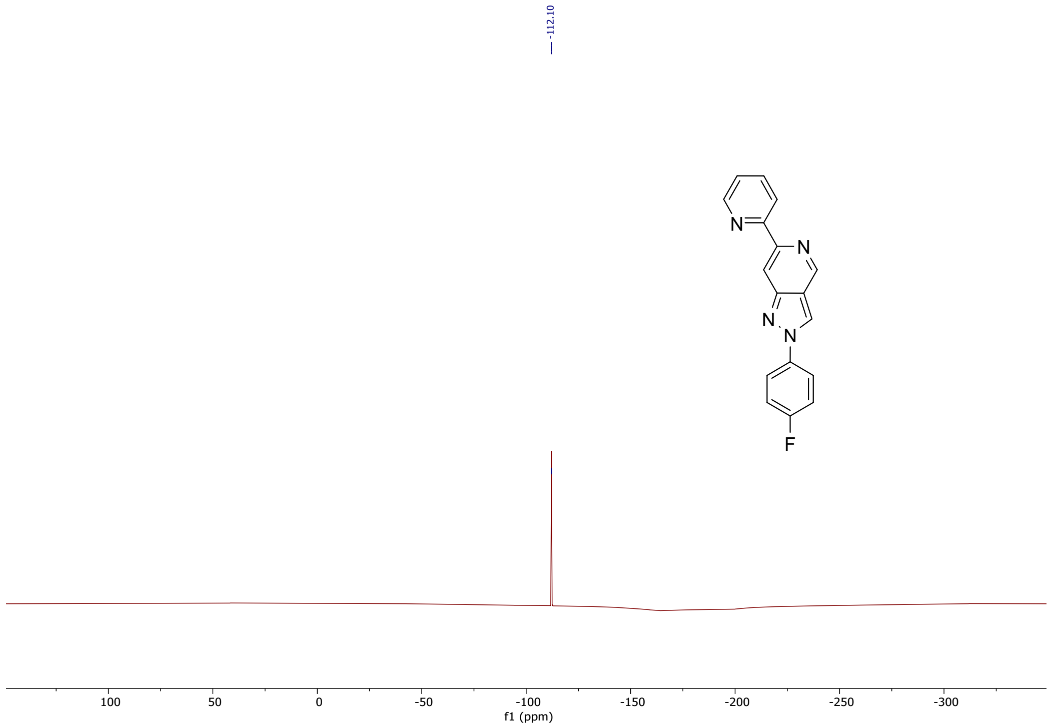

Figure S24. ^{19}F NMR spectrum (376 MHz, $CDCl_3$) of 2-(4-fluorophenyl)-6-(pyridin-2-yl)-2*H*-pyrazolo[4,3-*c*]pyridine (**7f**)

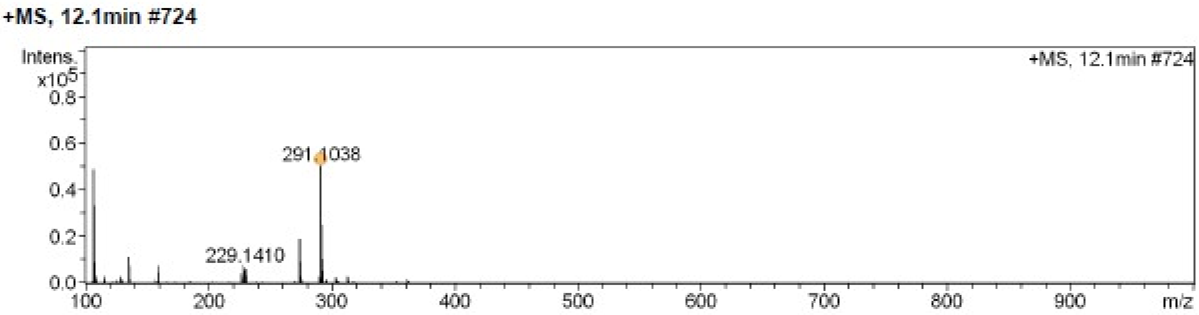

Meas. m/z	#	Ion Formula	m/z	err [ppm]	mSigma	# Sigma	Score	rdb	e⁻ Conf	N-Rule
291.1038	1	C17H12FN4	291.1041	1.0	6.5	2	100.00	13.5	even	ok

Figure S25. HRMS (ESI) report of **7f**

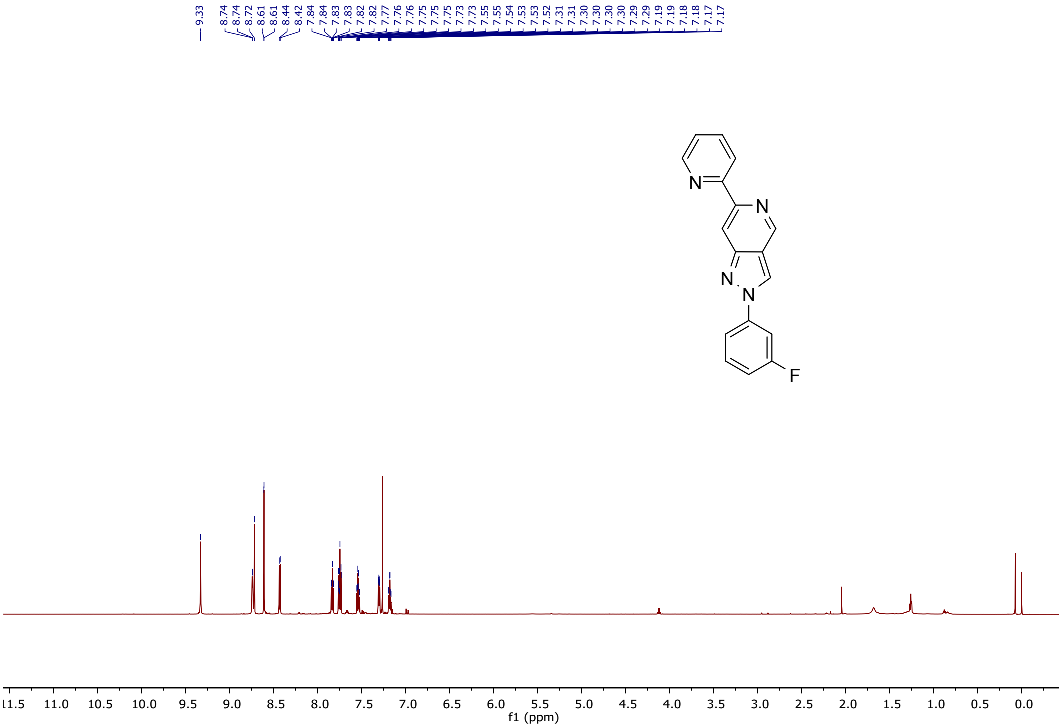

Figure S26. ^{1}H NMR spectrum (700 MHz, $CDCl_3$) of 2-(3-fluorophenyl)-6-(pyridin-2-yl)-2*H*-pyrazolo[4,3-*c*]pyridine (**7g**)

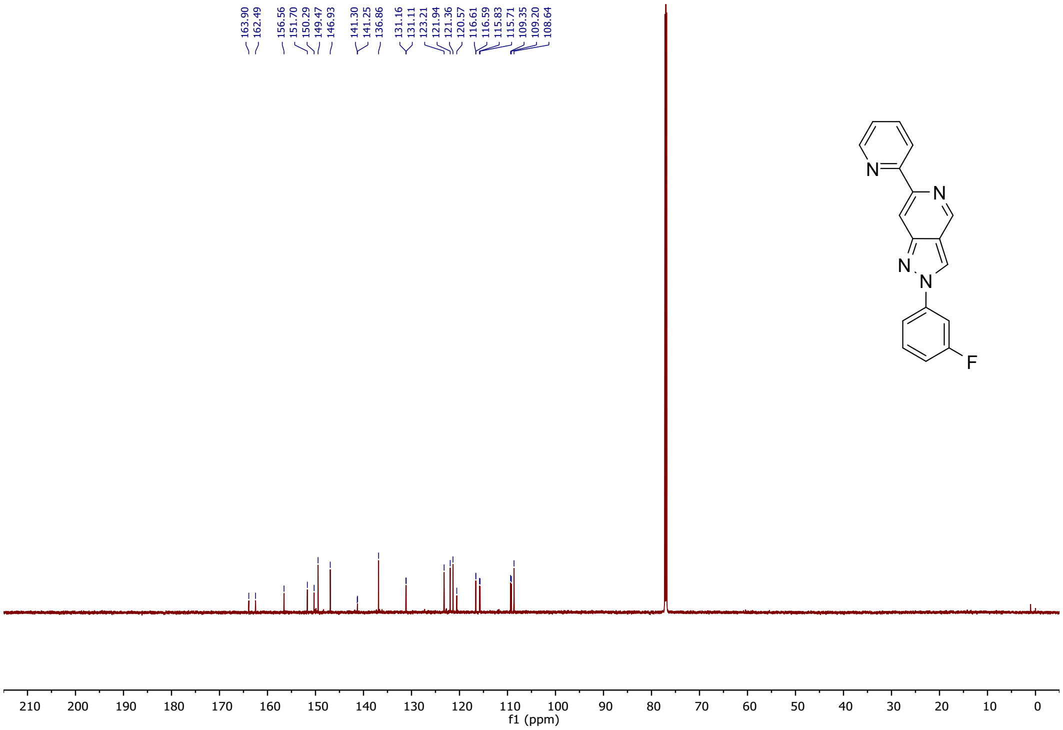

Figure S27. ^{13}C NMR spectrum (176 MHz, $CDCl_3$) of 2-(3-fluorophenyl)-6-(pyridin-2-yl)-2*H*-pyrazolo[4,3-*c*]pyridine (**7g**)

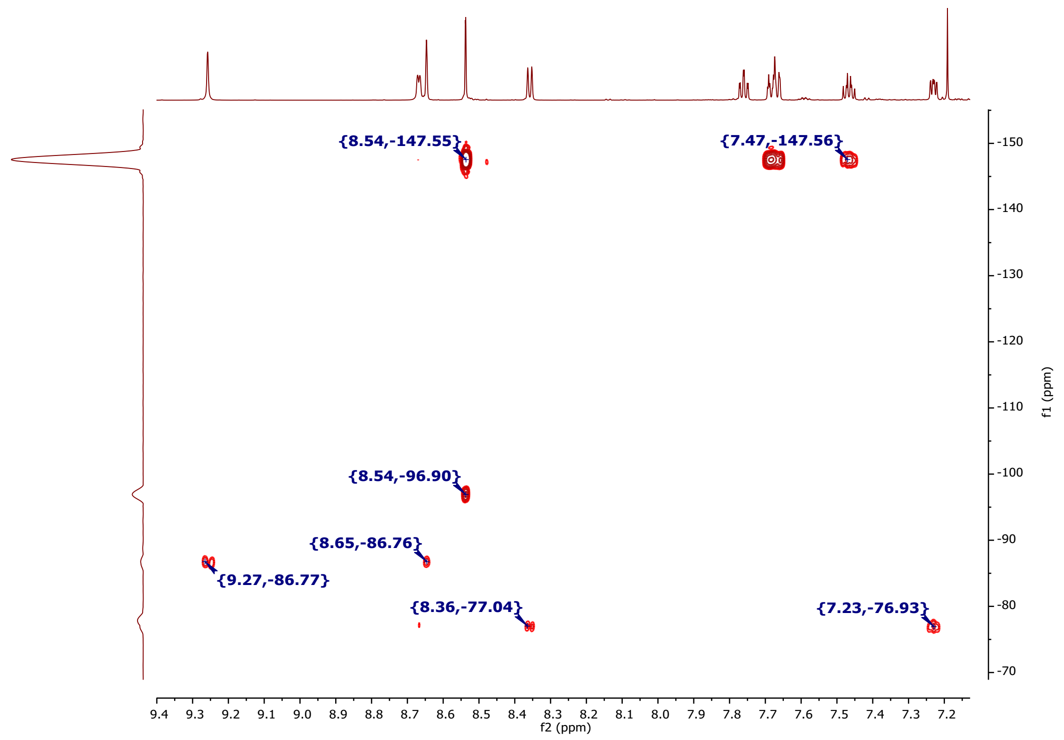

Figure S28. ^{1}H-^{15}N HMBC NMR spectrum (71 MHz, $CDCl_3$) of 2-(3-fluorophenyl)-6-(pyridin-2-yl)-2*H*-pyrazolo[4,3-*c*]pyridine (**7g**)

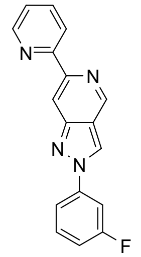

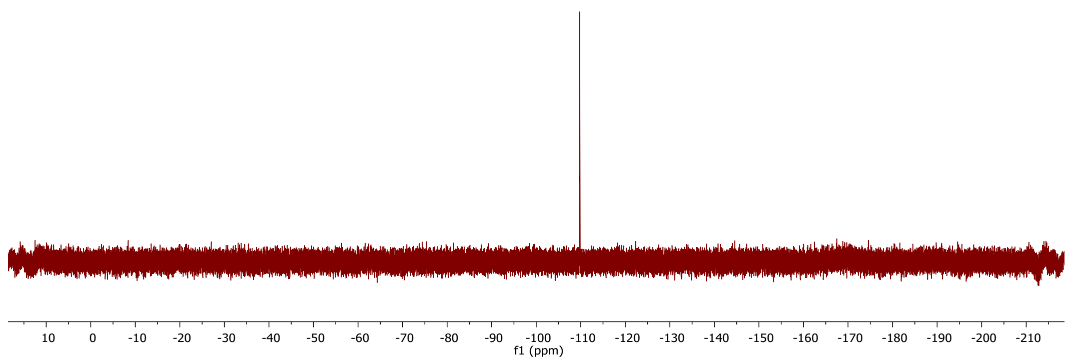

Figure S29. ^{19}F NMR spectrum (376 MHz, $CDCl_3$) of 2-(3-fluorophenyl)-6-(pyridin-2-yl)-2*H*-pyrazolo[4,3-*c*]pyridine (**7g**)

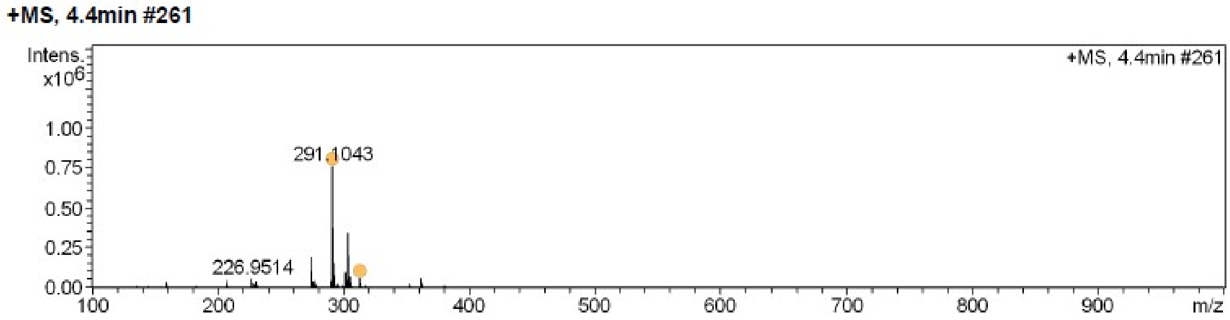

Meas. m/z	#	Ion Formula	m/z	err [ppm]	mSigma	# Sigma	Score	rdb	e⁻ Conf	N-Rule
291.1043	1	C17H12FN4	291.1041	-0.8	9.0	1	100.00	13.5	even	ok
313.0857	1	C17H11FN4Na	313.0860	0.8	3.0	1	100.00	13.5	even	ok

Figure S30. HRMS (ESI) report of **7g**

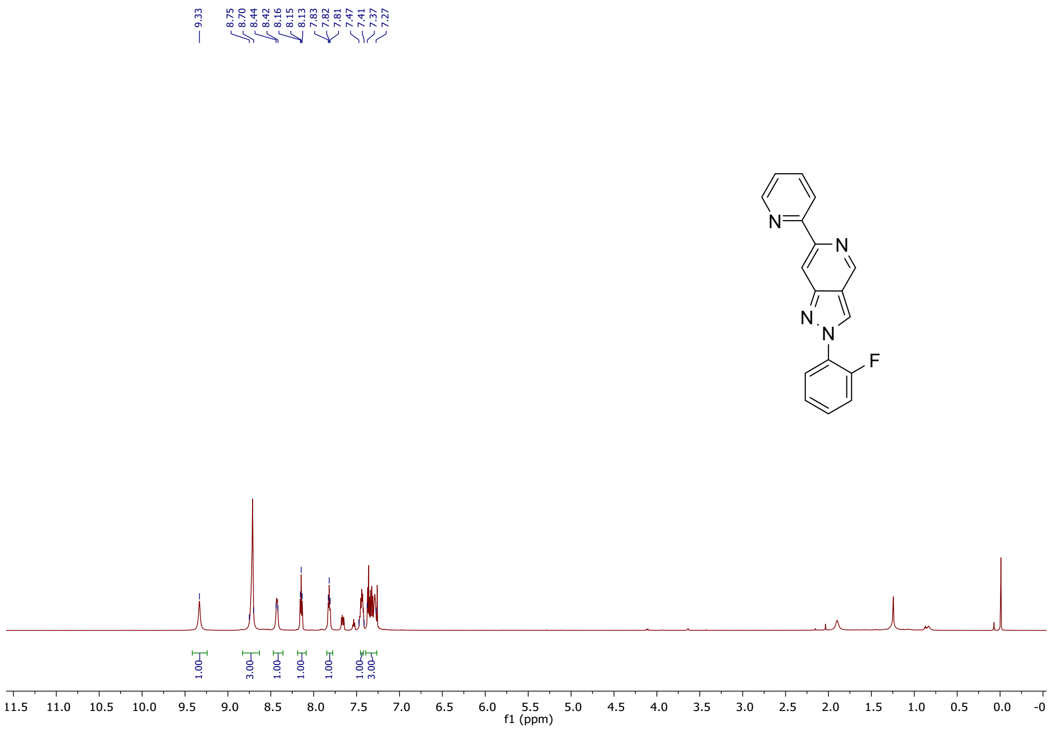

Figure S31. ^{1}H NMR spectrum (700 MHz, $CDCl_3$) of 2-(2-fluorophenyl)-6-(pyridin-2-yl)-2*H*-pyrazolo[4,3-*c*]pyridine (**7h**)

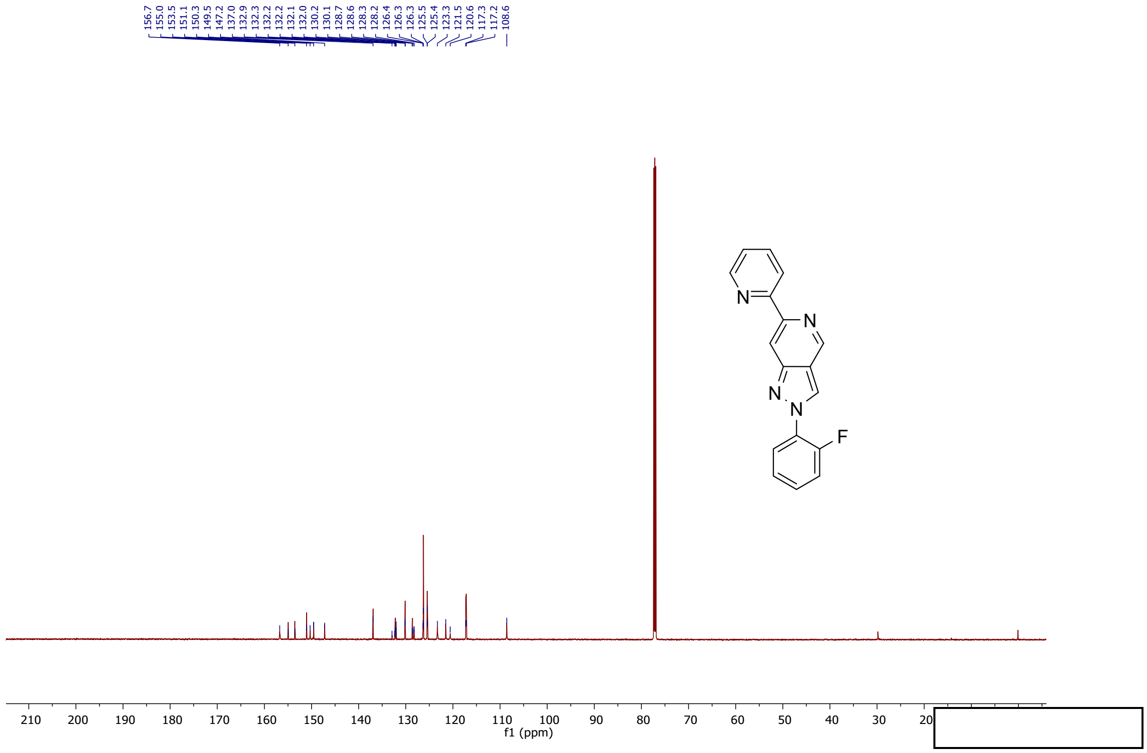

Figure S32. ^{13}C NMR spectrum (176 MHz, $CDCl_3$) of 2-(2-fluorophenyl)-6-(pyridin-2-yl)-2*H*-pyrazolo[4,3-*c*]pyridine (**7h**)

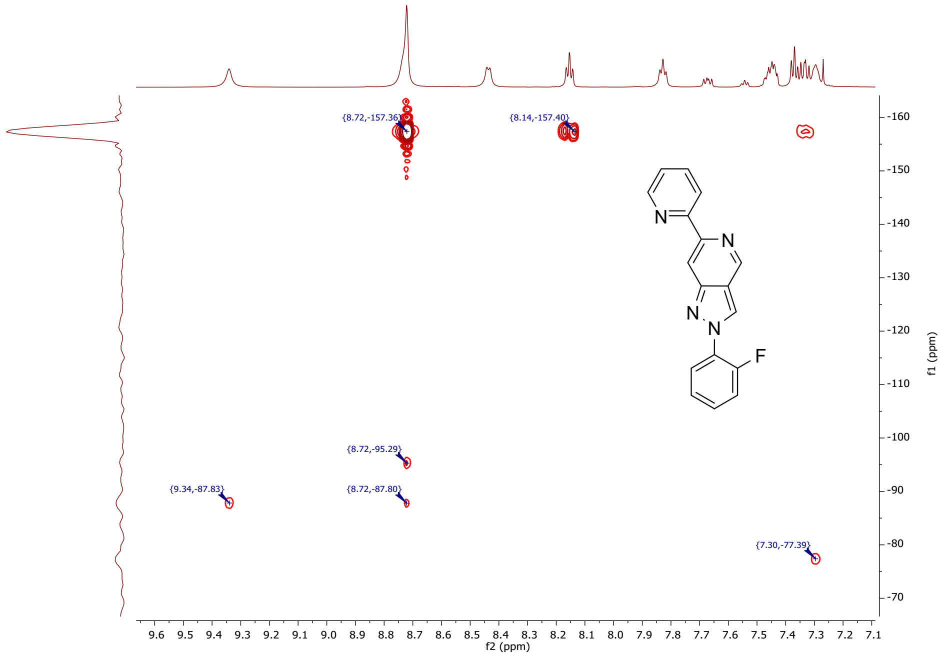

Figure S33. ^{1}H-^{15}N HMBC NMR spectrum (71 MHz, $CDCl_3$) of 2-(2-fluorophenyl)-6-(pyridin-2-yl)-2*H*-pyrazolo[4,3-*c*]pyridine (**7h**)

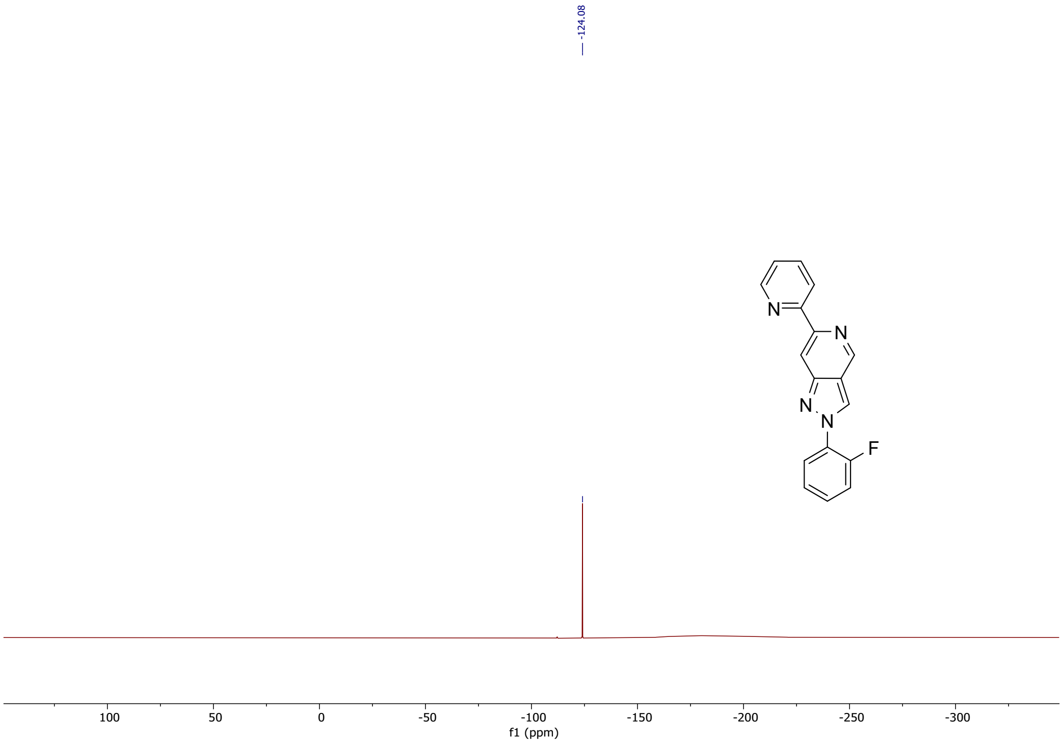

Figure S34. ^{19}F NMR spectrum (376 MHz, $CDCl_3$) of 2-(2-fluorophenyl)-6-(pyridin-2-yl)-2*H*-pyrazolo[4,3-*c*]pyridine (**7h**)

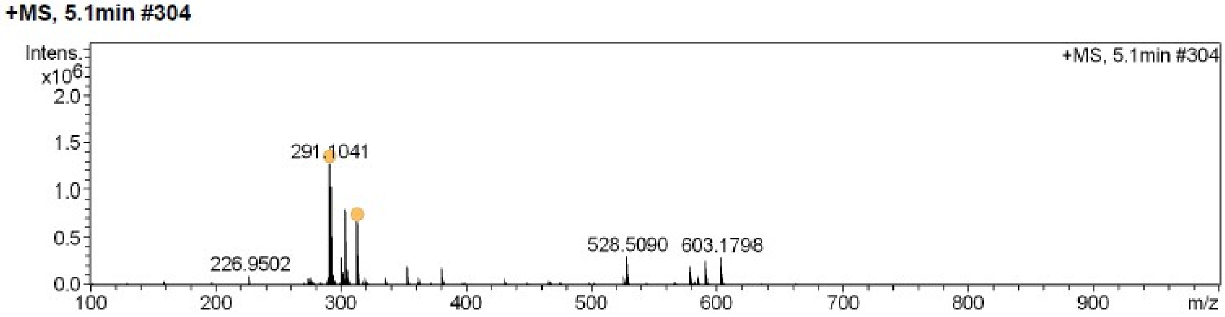

Meas. m/z	#	Ion Formula	m/z	err [ppm]	mSigma	# Sigma	Score	rdb	e¯ Conf	N-Rule
291.1041	1	C17H12FN4	291.1041	0.2	351.4	1	100.00	13.5	even	ok
313.0855	1	C17H11FN4Na	313.0860	-1.7	1.1	1	100.00	13.5	even	ok
	1	C17H11FN4Na	313.0860	-1.7	1.1	1	100.00	13.5	even	ok

Figure S35. HRMS (ESI) report of **7h**
