# Supplementary material for: Synthesis and antimitotic activity of 2-phenyl-6-pyridinyl-2H-pyrazolo[4,3-c]pyridines
Source: RSC Adv. 2026 May 12;16(27):24822–37. doi: 10.1039/d5ra09208f (PMC13161753; doi:10.1039/d5ra09208f)
Supplement: RA-016-D5RA09208F-s002 [file RA-016-D5RA09208F-s002.pdf]

Supplementary Information (SI) for RSC Advances.
This journal is © The Royal Society of Chemistry 2026

Supporting Information

Synthesis and antimitotic activity of 2-phenyl-6-pyridinyl-2*H*-pyrazolo[4,3-*c*]pyridines

Vaida Aleksienė,[†a] Eva Řezníčková,[†b] Aurimas Bieliauskas,[a] Veronika Vojáčková,[b] Veronika Molitorová,[b] Austėja Šalvytė-Nikliauzienė,[c] Sergey Belyakov,[d] Asta Žukauskaitė,[e] Eglė Arbačiauskienė,[c] Vladimír Kryštof*[b,f] and Algirdas Šačkus*[a,c]

[a] Institute of Synthetic Chemistry, Faculty of Chemical Technology, Kaunas University of Technology, K. Baršausko g. 59, LT-51423 Kaunas, Lithuania. E-mail: algirdas.sackus@ktu.lt

[b] Department of Experimental Biology, Faculty of Science, Palacký University, Šlechtitelů 27, CZ-77900 Olomouc, Czech Republic. E-mail: vladimir.krystof@upol.cz

[c] Department of Organic Chemistry, Faculty of Chemical Technology, Kaunas University of Technology, Radvilėnų pl. 19, LT-50254 Kaunas, Lithuania

[d] Latvian Institute of Organic Synthesis, Aizkraukles 21, LV-1006 Riga, Latvia

[e] Department of Chemical Biology, Faculty of Science, Palacký University, Šlechtitelů 27, CZ-77900 Olomouc, Czech Republic

[f] Institute of Molecular and Translational Medicine, Faculty of Medicine and Dentistry, Palacký University, Hněvotínská 5, CZ-77900 Olomouc, Czech Republic

† Vaida Aleksienė and Eva Řezníčková contributed equally to this work

* Correspondence:

Algirdas Šačkus
algirdas.sackus@ktu.lt

Vladimír Kryštof
vladimir.krystof@upol.cz

K562 / **7f** (µM) / 24h treatment
0 – 0.020 – 0.078 – 0.313 – 1.25 – 5 – 0

K562 / **7f** (µM) / 24h treatment
0 – 0.020 – 0.078 – 0.313 – 1.25 – 5 – 0

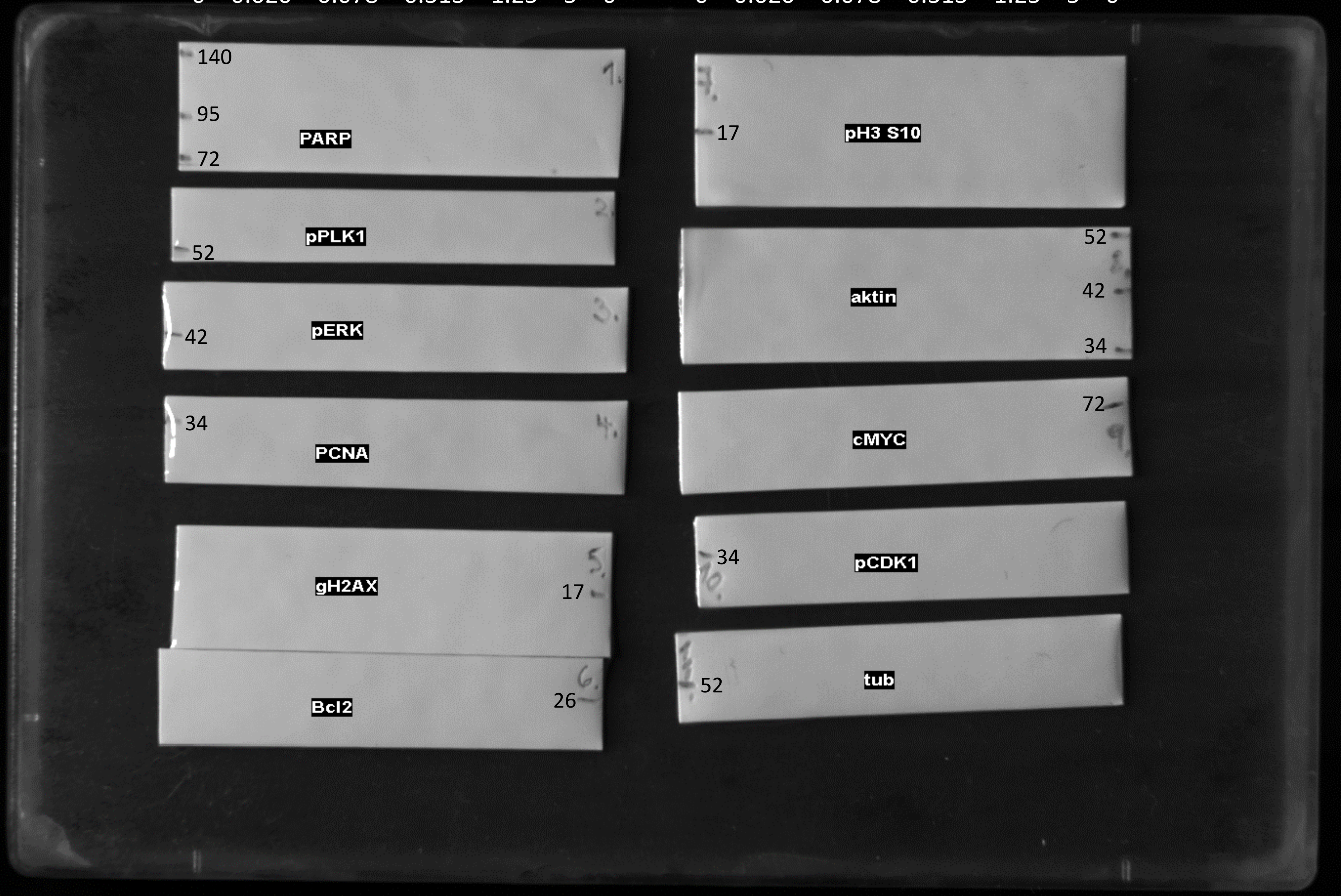

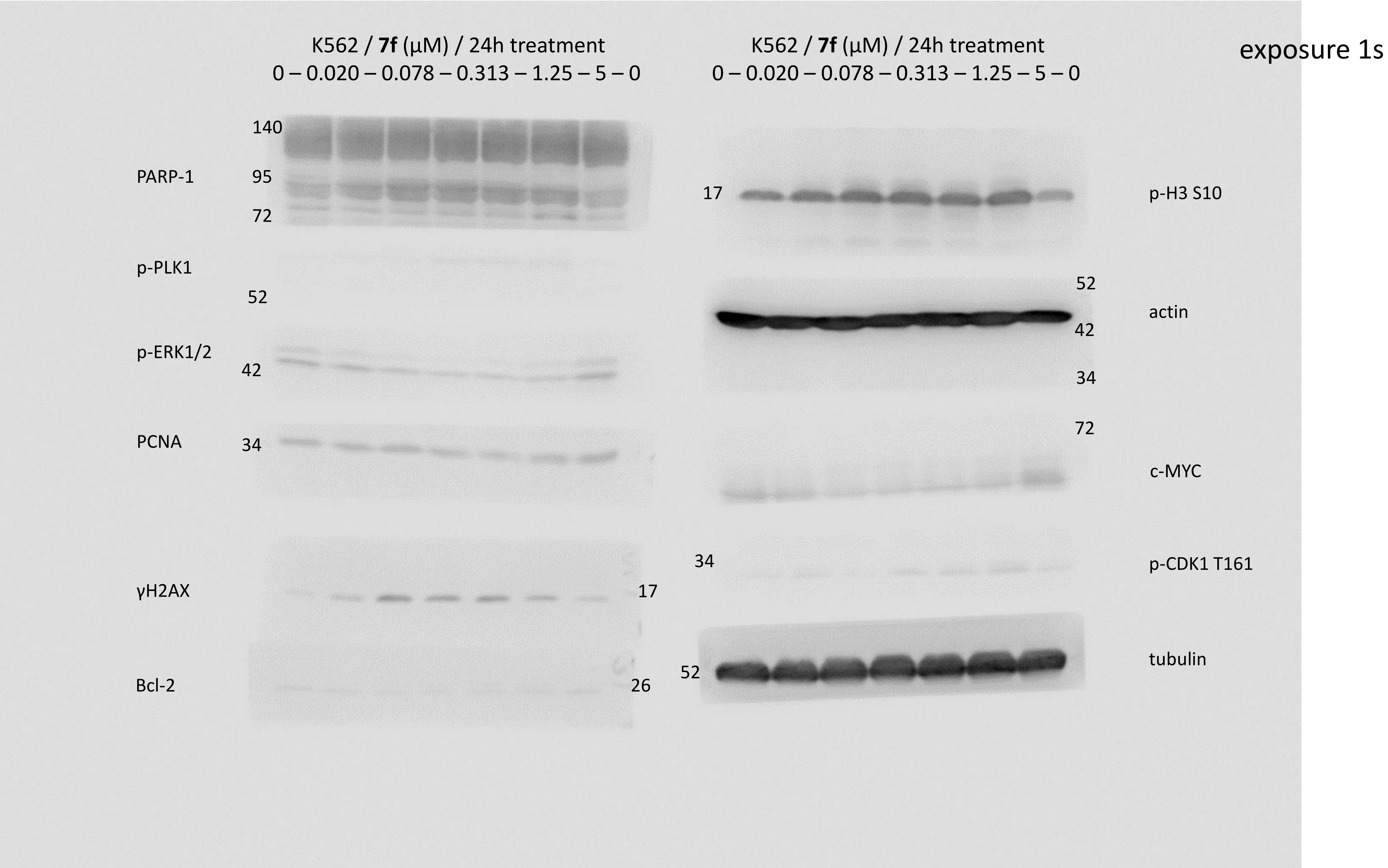
K562 / 7f (µM) / 24h treatment
0 – 0.020 – 0.078 – 0.313 – 1.25 – 5 – 0
K562 / 7f (µM) / 24h treatment
0 – 0.020 – 0.078 – 0.313 – 1.25 – 5 – 0
exposure 1s
PARP-1
140
95
72
p-PLK1
52
p-ERK1/2
42
PCNA
34
γH2AX
17
Bcl-2
26
17
p-H3 S10
52
actin
42
34
72
c-MYC
34
p-CDK1 T161
52
tubulin

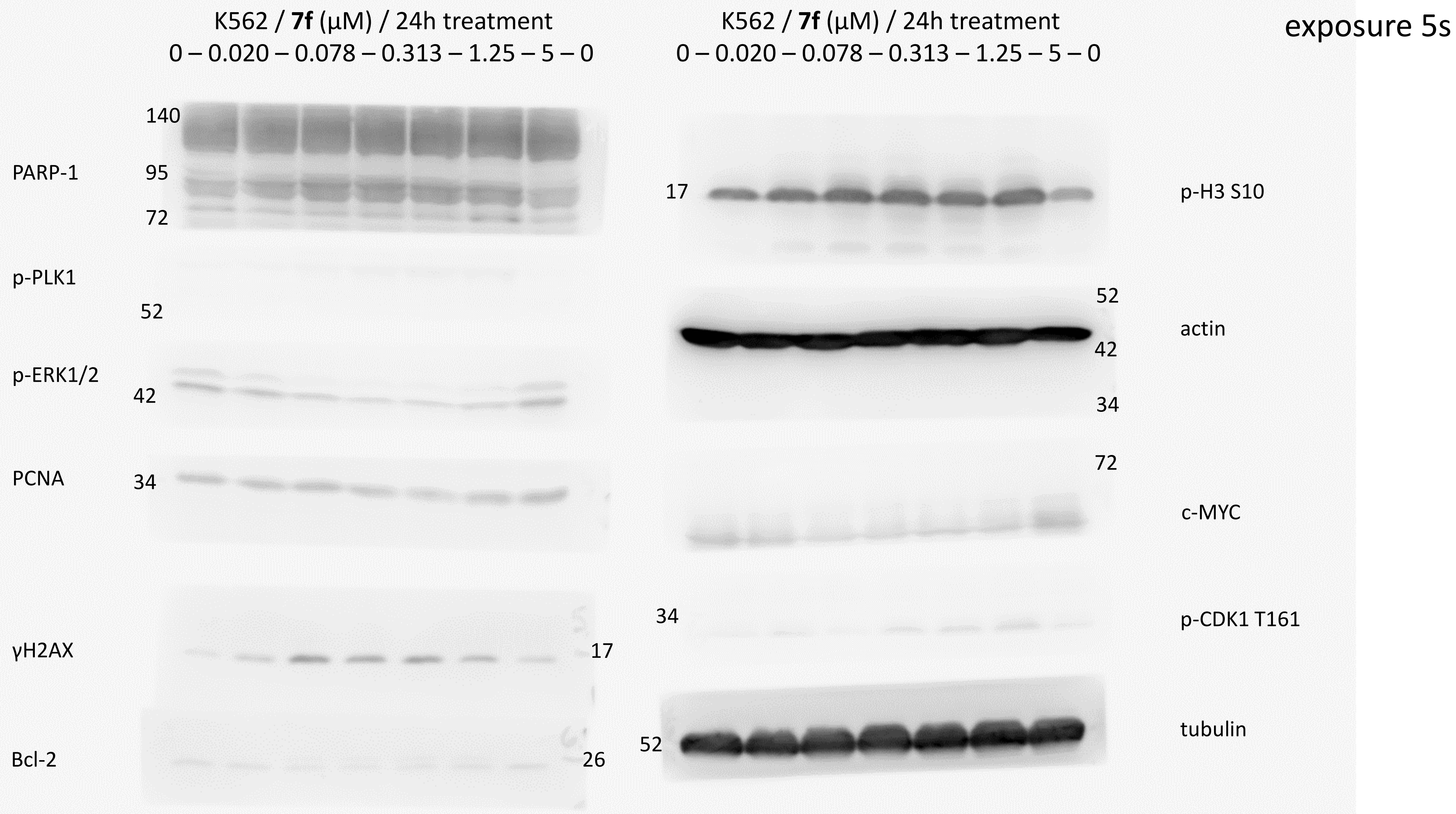
K562 / 7f (µM) / 24h treatment
0 – 0.020 – 0.078 – 0.313 – 1.25 – 5 – 0
K562 / 7f (µM) / 24h treatment
0 – 0.020 – 0.078 – 0.313 – 1.25 – 5 – 0
exposure 5s
PARP-1
140
95
72
p-PLK1
52
p-ERK1/2
42
PCNA
34
γH2AX
17
Bcl-2
26
p-H3 S10
17
actin
52
42
34
c-MYC
72
p-CDK1 T161
34
tubulin
52

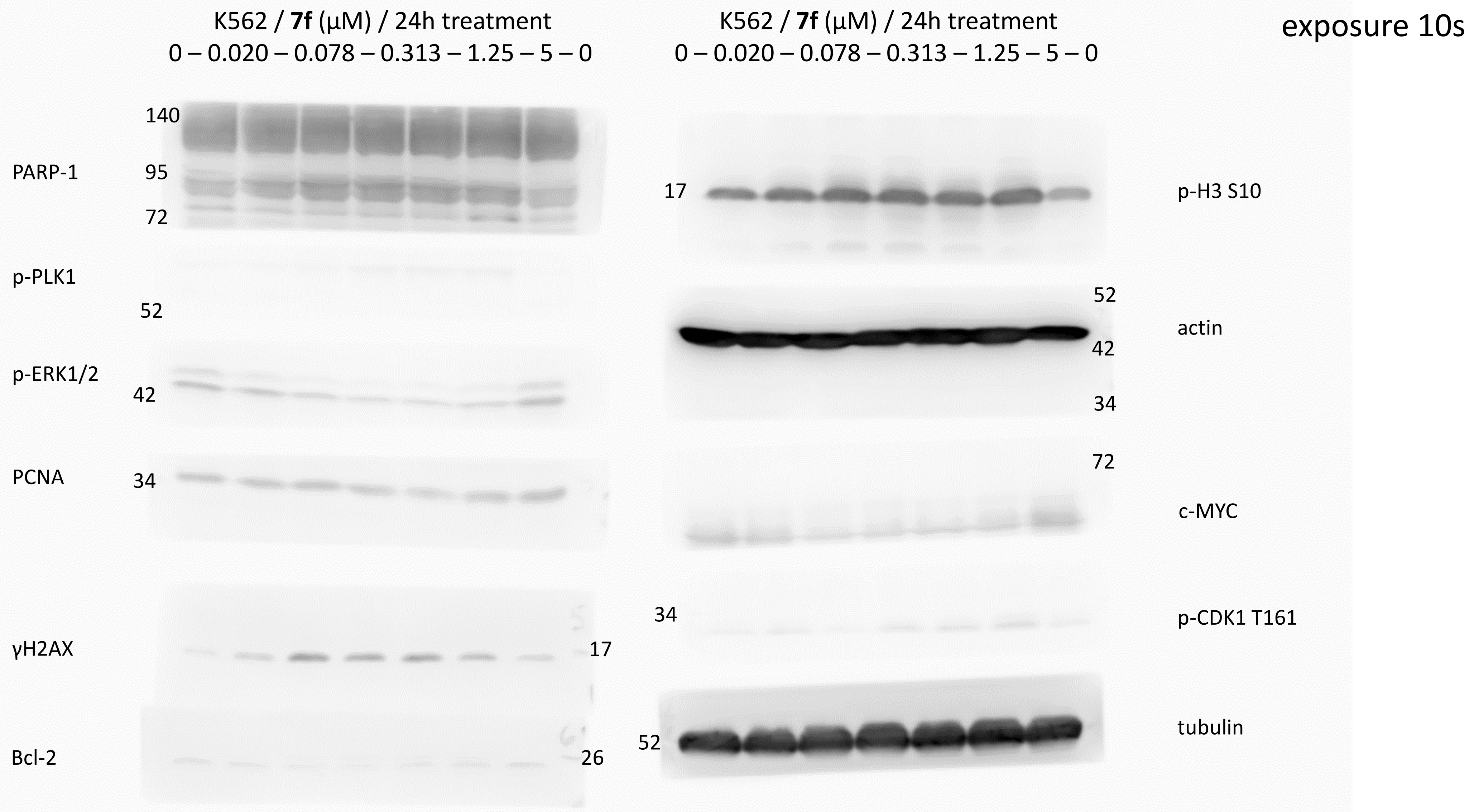
K562 / 7f (µM) / 24h treatment
0 – 0.020 – 0.078 – 0.313 – 1.25 – 5 – 0
K562 / 7f (µM) / 24h treatment
0 – 0.020 – 0.078 – 0.313 – 1.25 – 5 – 0
exposure 10s
PARP-1
140
95
72
p-PLK1
52
p-ERK1/2
42
PCNA
34
γH2AX
17
Bcl-2
26
17
p-H3 S10
52
actin
42
34
72
c-MYC
34
p-CDK1 T161
52
tubulin

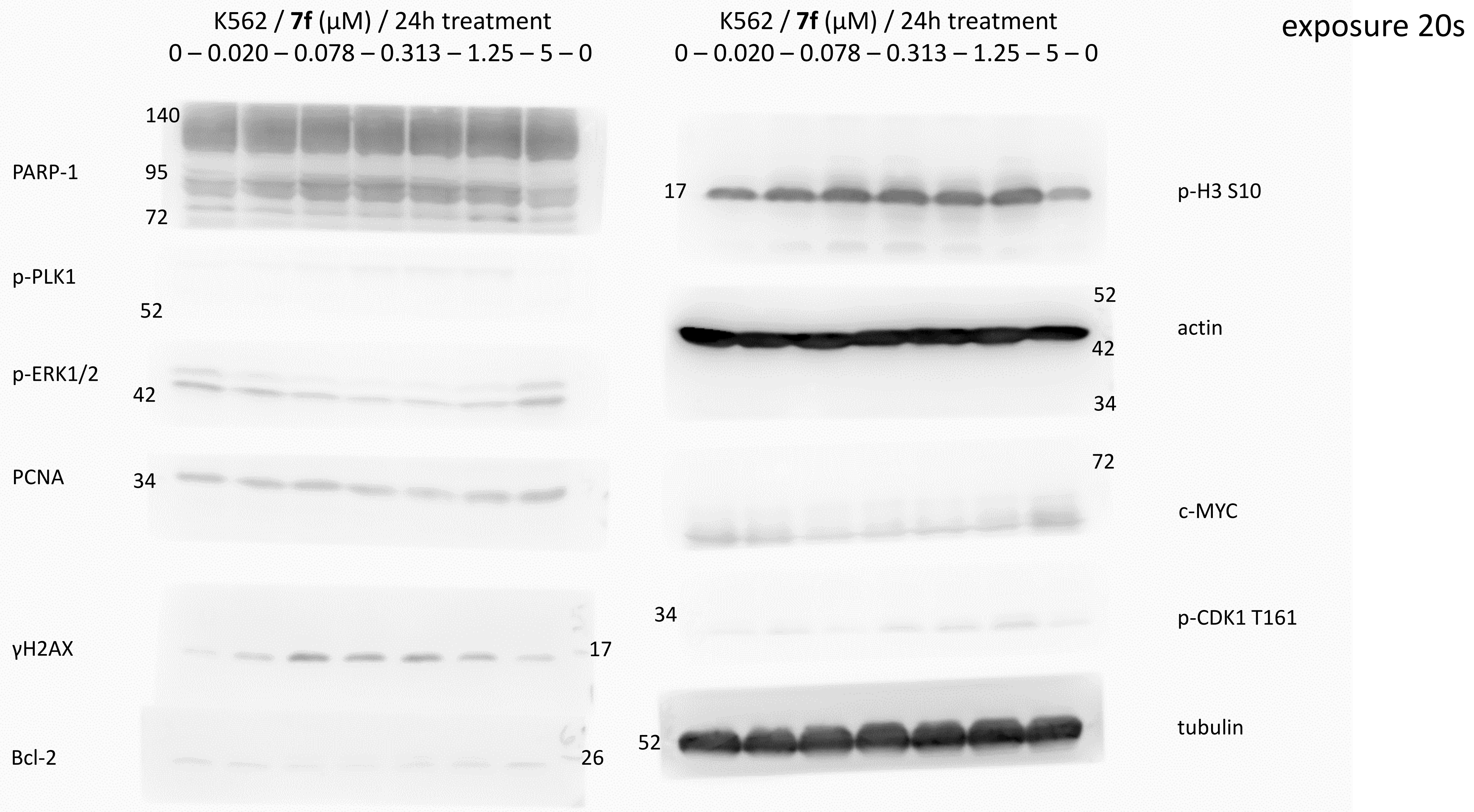
K562 / 7f (µM) / 24h treatment
0 – 0.020 – 0.078 – 0.313 – 1.25 – 5 – 0
K562 / 7f (µM) / 24h treatment
0 – 0.020 – 0.078 – 0.313 – 1.25 – 5 – 0
exposure 20s
PARP-1
140
95
72
p-PLK1
52
p-ERK1/2
42
PCNA
34
γH2AX
17
Bcl-2
26
p-H3 S10
17
actin
52
42
34
c-MYC
72
p-CDK1 T161
34
tubulin
52

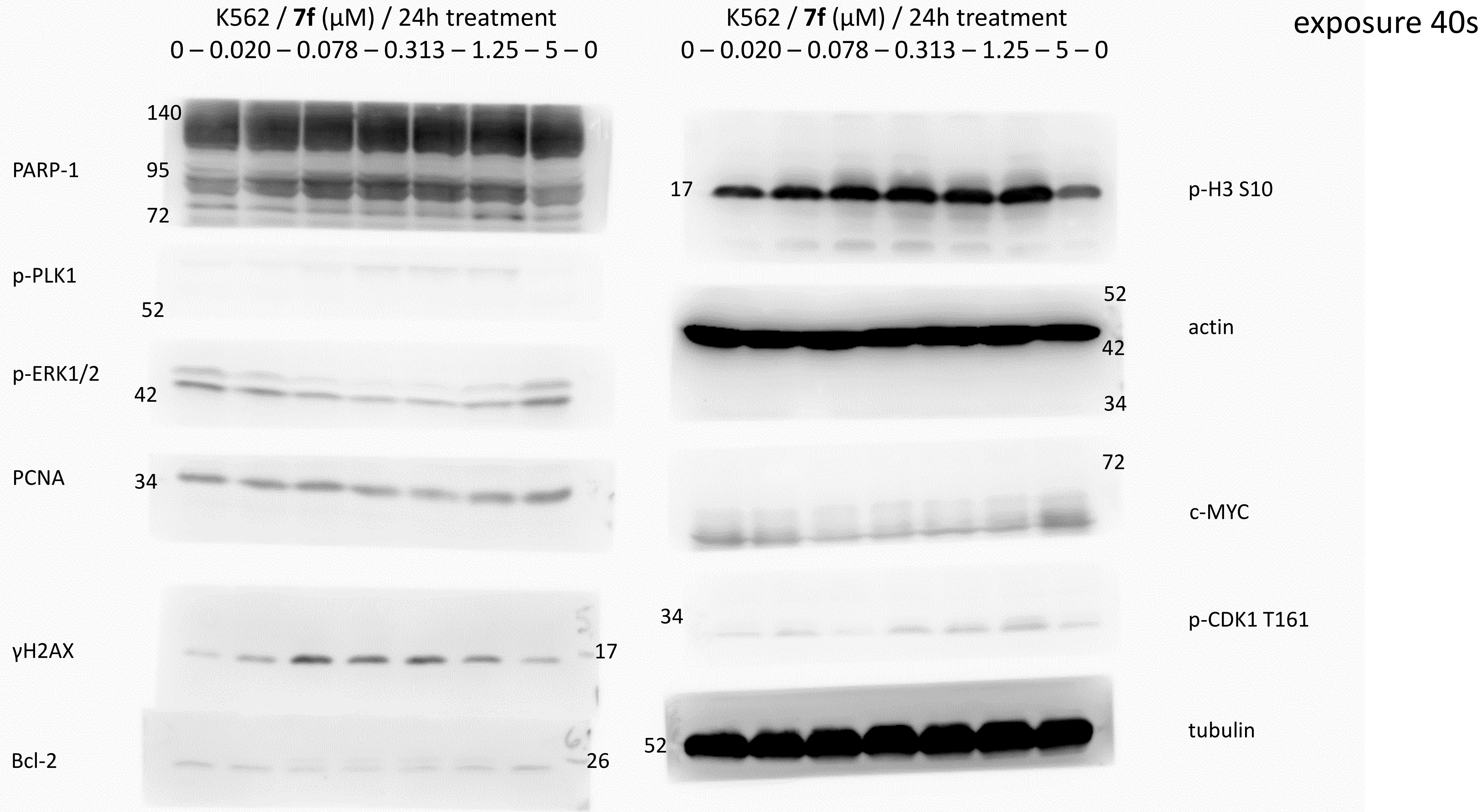
K562 / **7f** (µM) / 24h treatment
0 – 0.020 – 0.078 – 0.313 – 1.25 – 5 – 0
K562 / **7f** (µM) / 24h treatment
0 – 0.020 – 0.078 – 0.313 – 1.25 – 5 – 0
exposure 40s
PARP-1
140
95
72
p-PLK1
52
p-ERK1/2
42
PCNA
34
γH2AX
17
Bcl-2
26
p-H3 S10
17
actin
52
42
34
c-MYC
72
p-CDK1 T161
34
tubulin
52

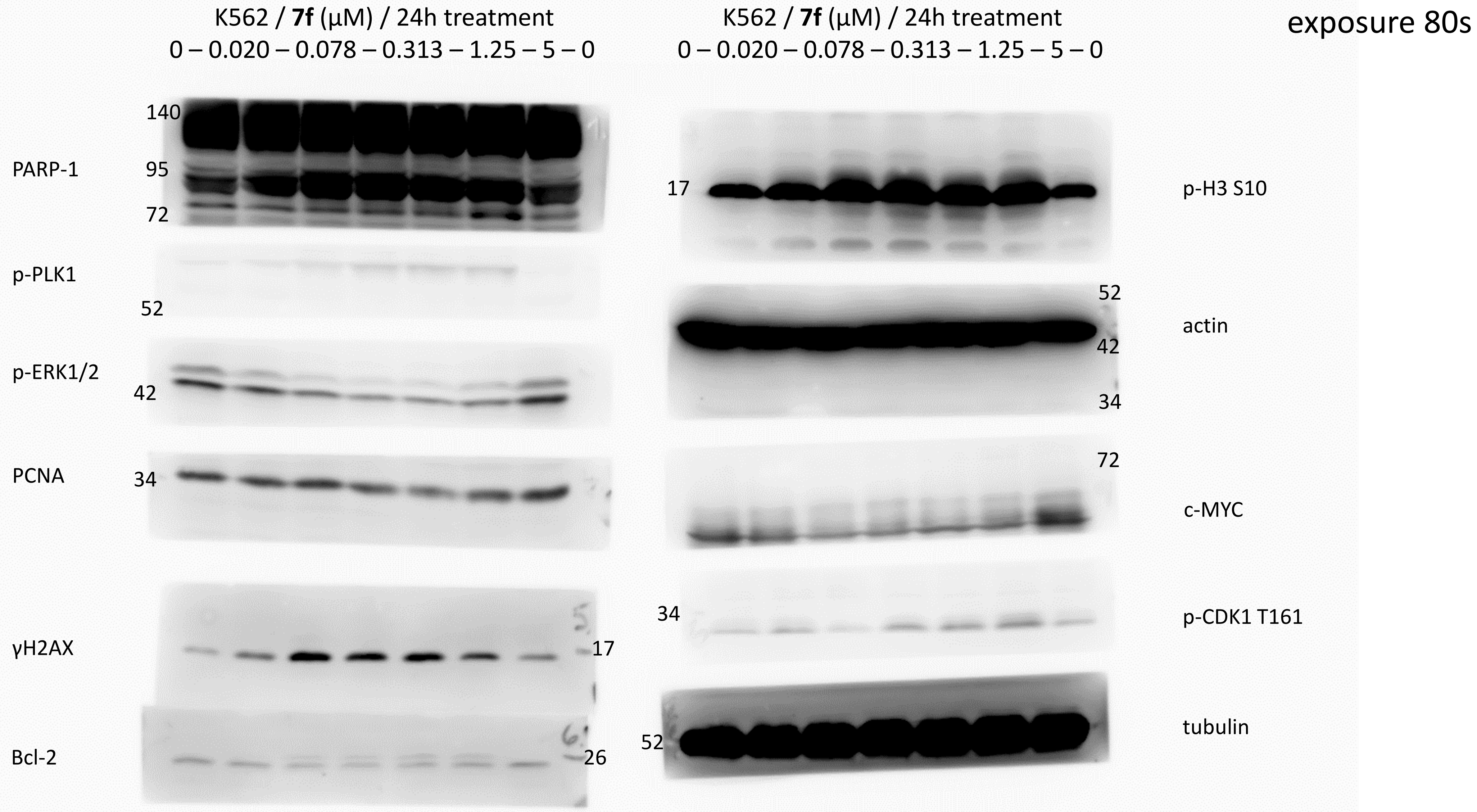
K562 / 7f (µM) / 24h treatment
0 – 0.020 – 0.078 – 0.313 – 1.25 – 5 – 0
K562 / 7f (µM) / 24h treatment
0 – 0.020 – 0.078 – 0.313 – 1.25 – 5 – 0
exposure 80s
PARP-1
140
95
72
p-PLK1
52
p-ERK1/2
42
PCNA
34
γH2AX
17
Bcl-2
26
p-H3 S10
17
actin
52
42
34
c-MYC
72
p-CDK1 T161
34
tubulin
52

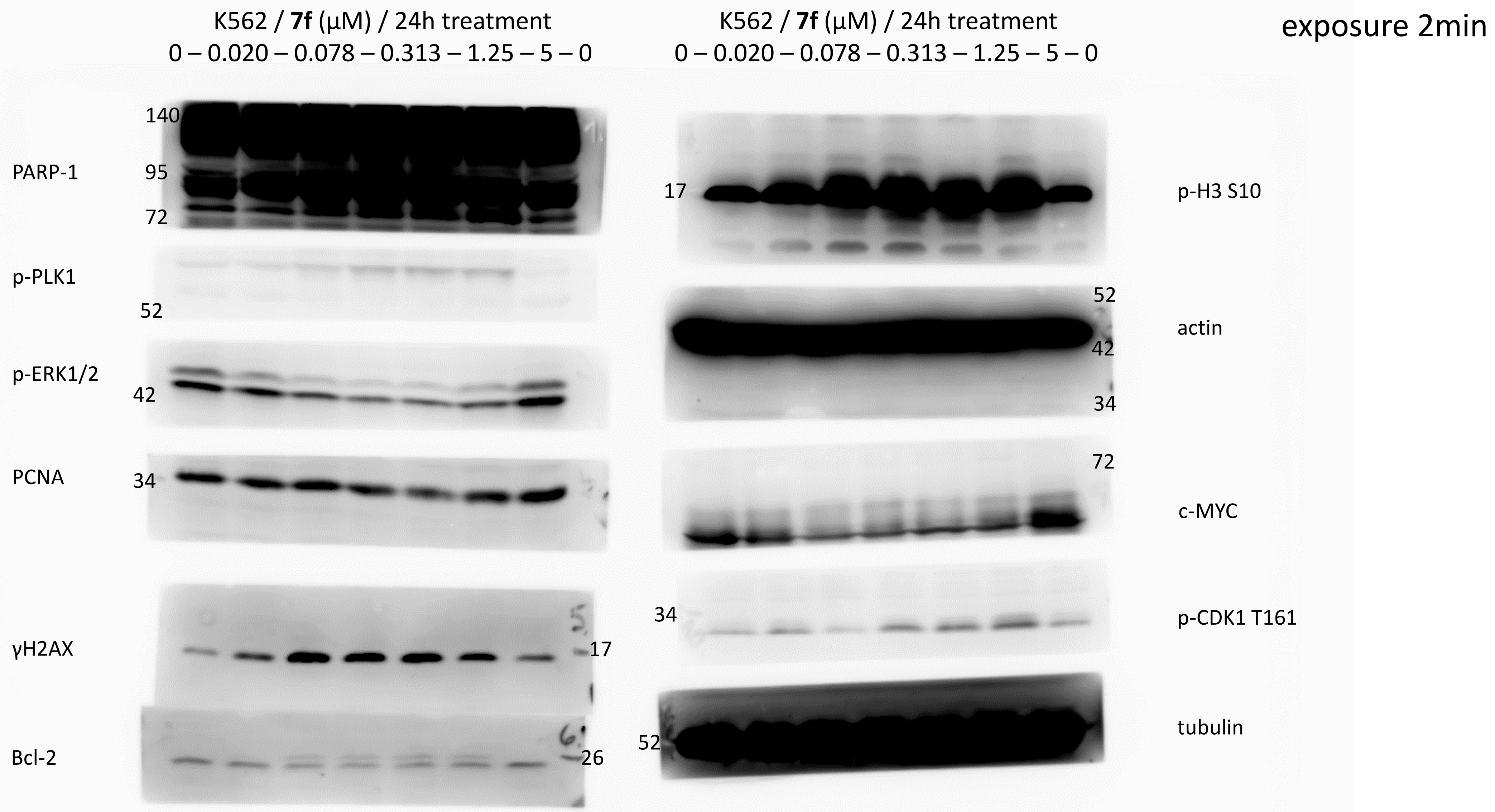
K562 / 7f (µM) / 24h treatment
0 – 0.020 – 0.078 – 0.313 – 1.25 – 5 – 0
K562 / 7f (µM) / 24h treatment
0 – 0.020 – 0.078 – 0.313 – 1.25 – 5 – 0
exposure 2min
PARP-1
140
95
72
p-PLK1
52
p-ERK1/2
42
PCNA
34
γH2AX
17
Bcl-2
26
p-H3 S10
17
actin
52
42
34
c-MYC
72
p-CDK1 T161
34
tubulin
52

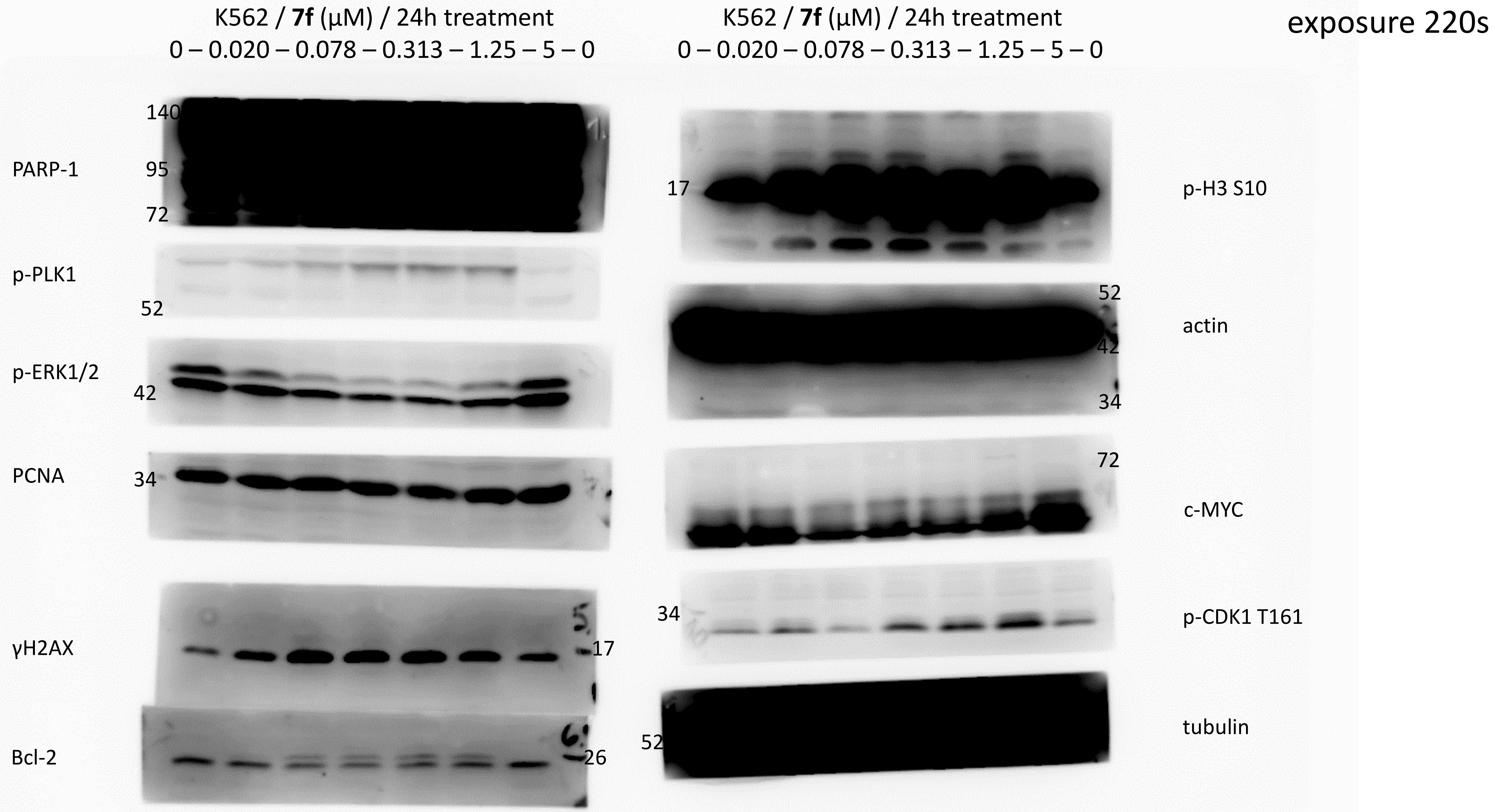
K562 / 7f (µM) / 24h treatment
0 – 0.020 – 0.078 – 0.313 – 1.25 – 5 – 0
K562 / 7f (µM) / 24h treatment
0 – 0.020 – 0.078 – 0.313 – 1.25 – 5 – 0
exposure 220s
PARP-1
140
95
72
p-PLK1
52
p-ERK1/2
42
PCNA
34
γH2AX
17
Bcl-2
26
17
p-H3 S10
52
actin
42
34
72
c-MYC
34
p-CDK1 T161
52
tubulin

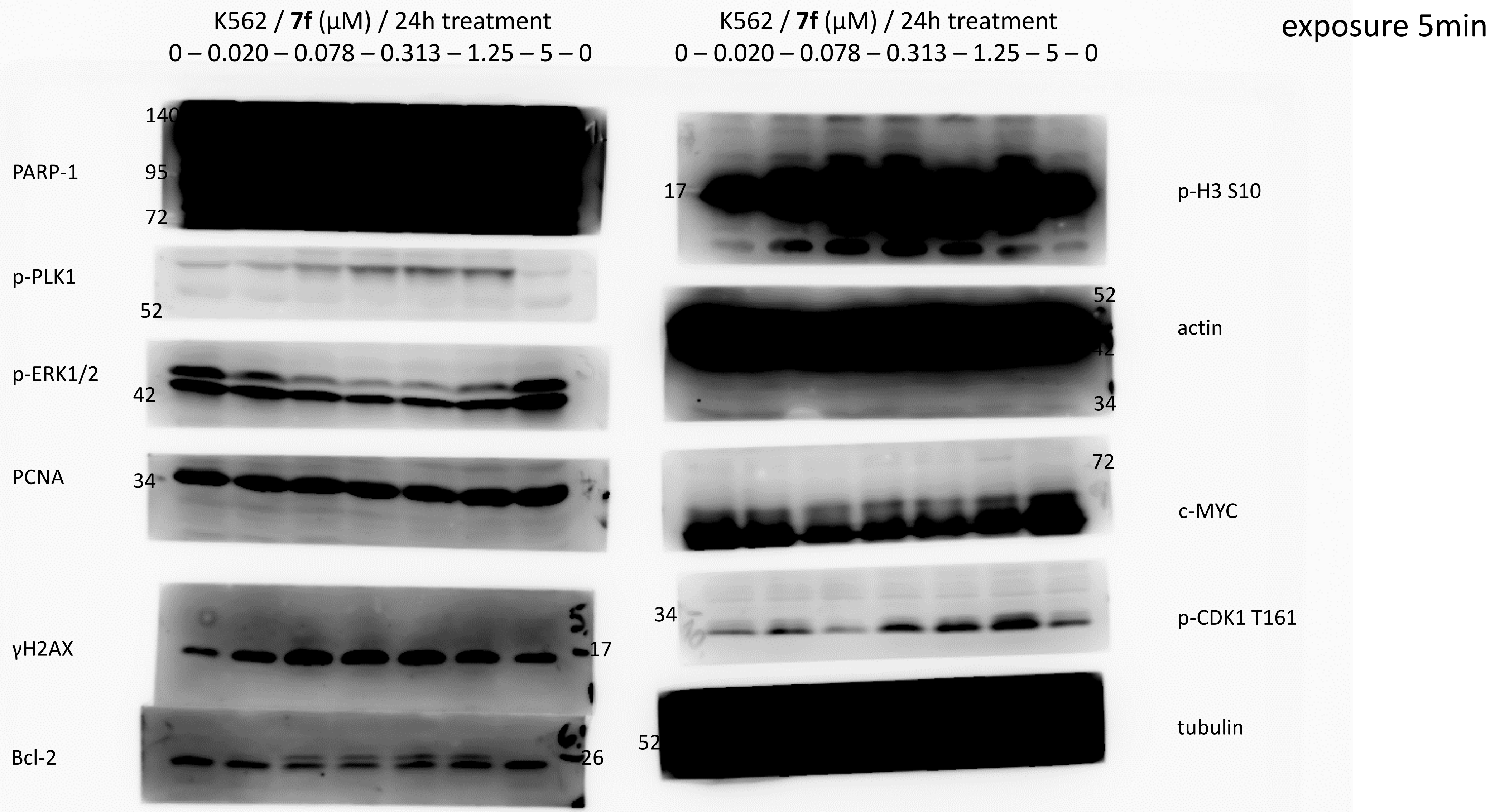

K562 / 7f (μM) / 24h treatment
0 – 0.020 – 0.078 – 0.313 – 1.25 – 5 – 0
K562 / 7f (μM) / 24h treatment
0 – 0.020 – 0.078 – 0.313 – 1.25 – 5 – 0
exposure 5min
PARP-1
140
95
72
p-PLK1
52
p-ERK1/2
42
PCNA
34
γH2AX
17
Bcl-2
26
p-H3 S10
17
actin
52
34
c-MYC
72
p-CDK1 T161
34
tubulin
52

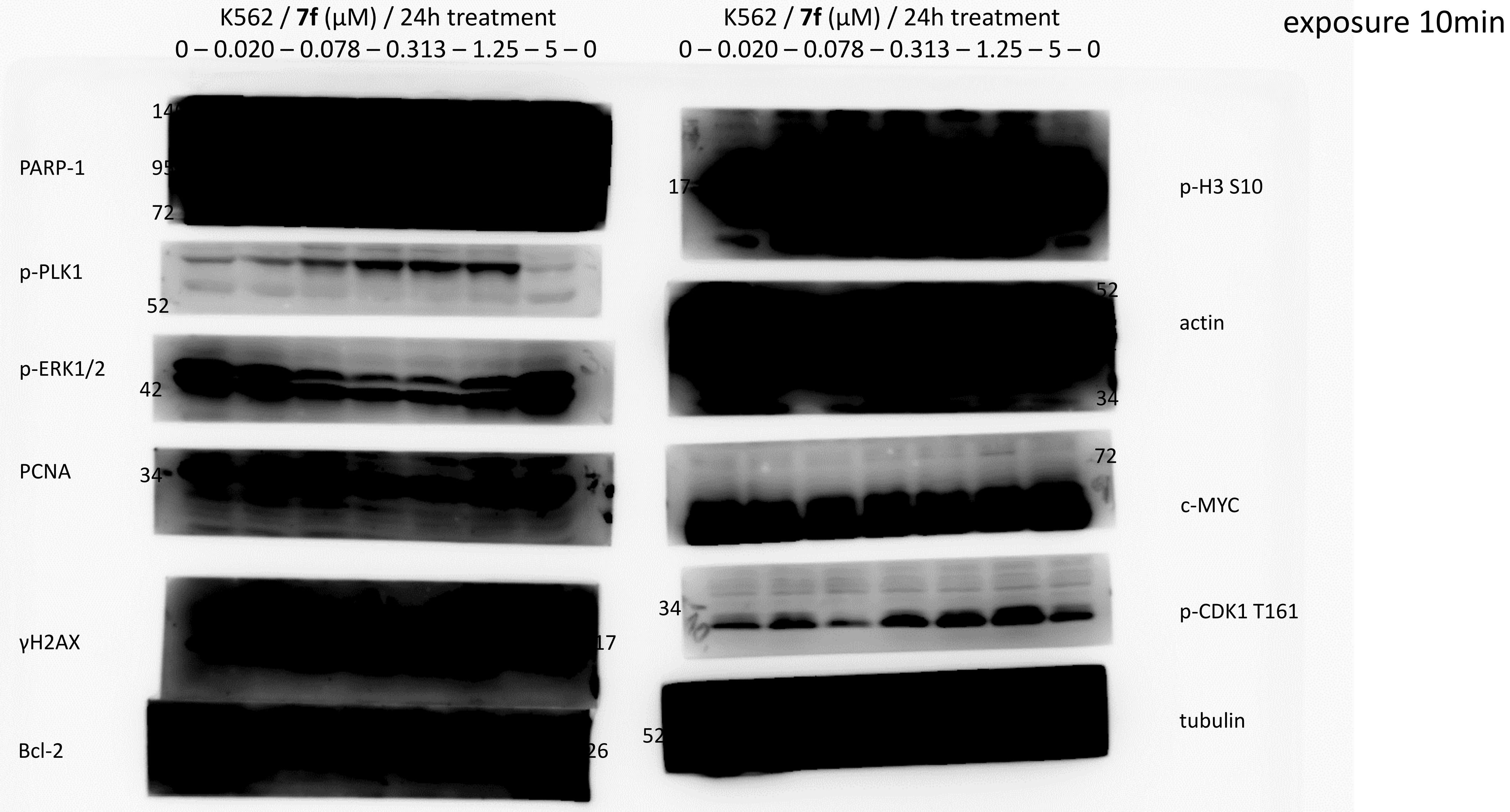
K562 / 7f (µM) / 24h treatment
0 – 0.020 – 0.078 – 0.313 – 1.25 – 5 – 0
K562 / 7f (µM) / 24h treatment
0 – 0.020 – 0.078 – 0.313 – 1.25 – 5 – 0
exposure 10min
PARP-1
p-PLK1
52
p-ERK1/2
42
PCNA
34
γH2AX
17
Bcl-2
26
p-H3 S10
17
actin
52
34
c-MYC
72
p-CDK1 T161
34
tubulin
52

K562 / **7f** (µM) /24h treatment

0 – 0.020 – 0.078 – 0.313 – 1.25 – 5 – 0

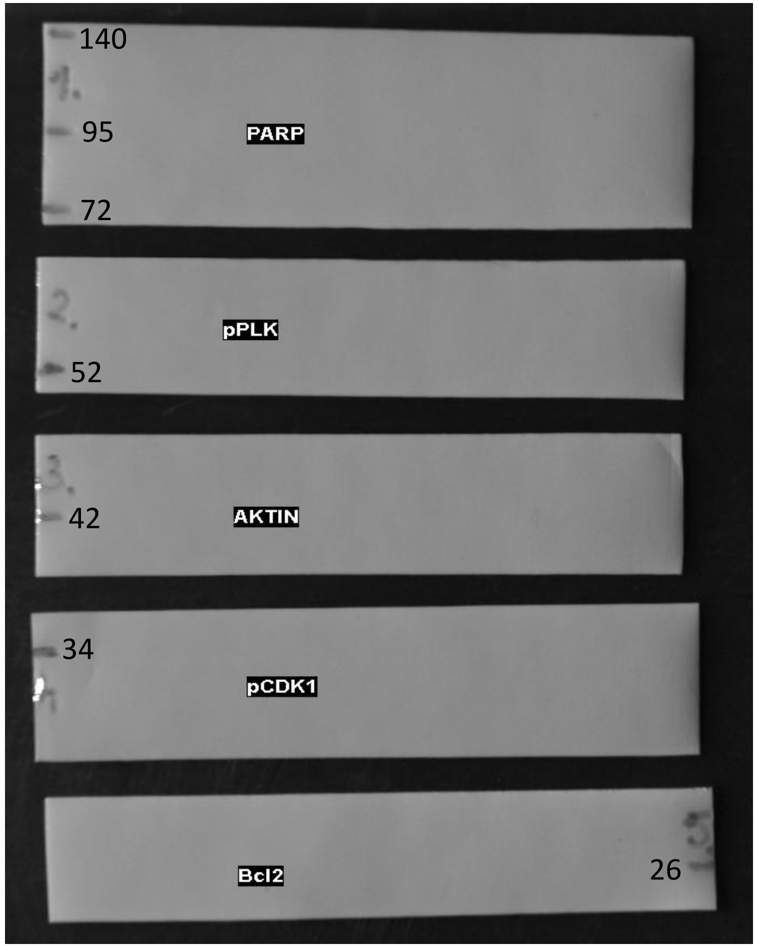

exposure 1s

K562 / **7f** (μM) /24h treatment
0 – 0.020 – 0.078 – 0.313 – 1.25 – 5 – 0

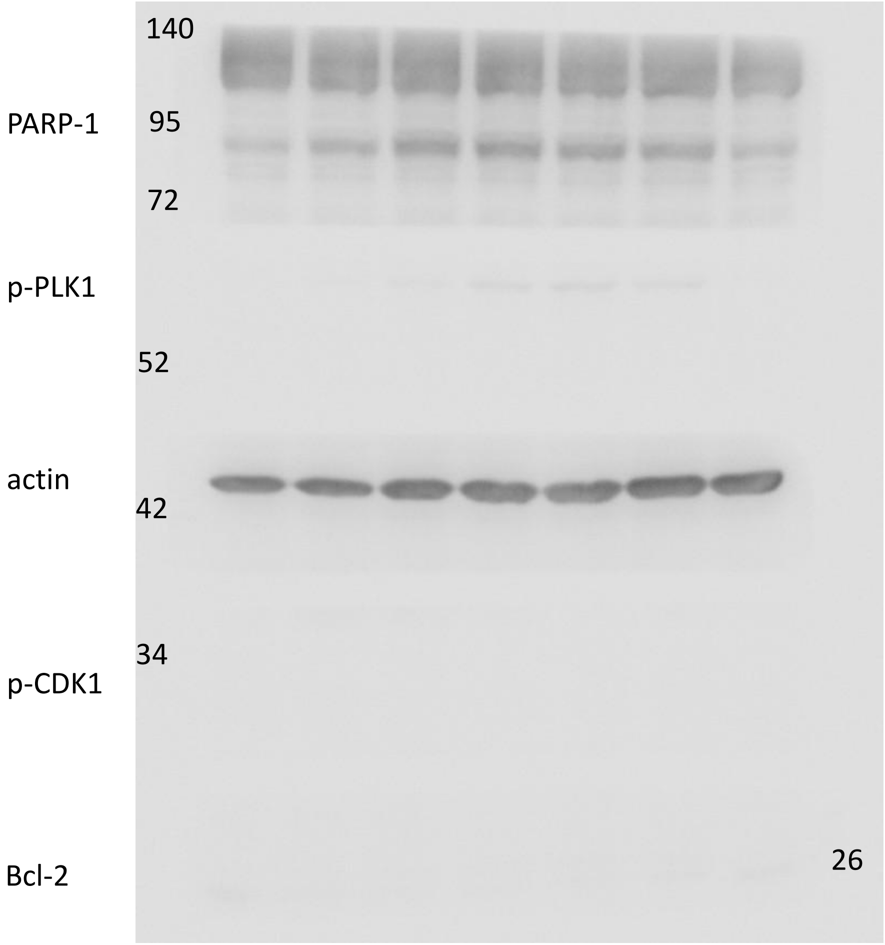

exposure 5s

K562 / **7f** (μM) /24h treatment
0 – 0.020 – 0.078 – 0.313 – 1.25 – 5 – 0

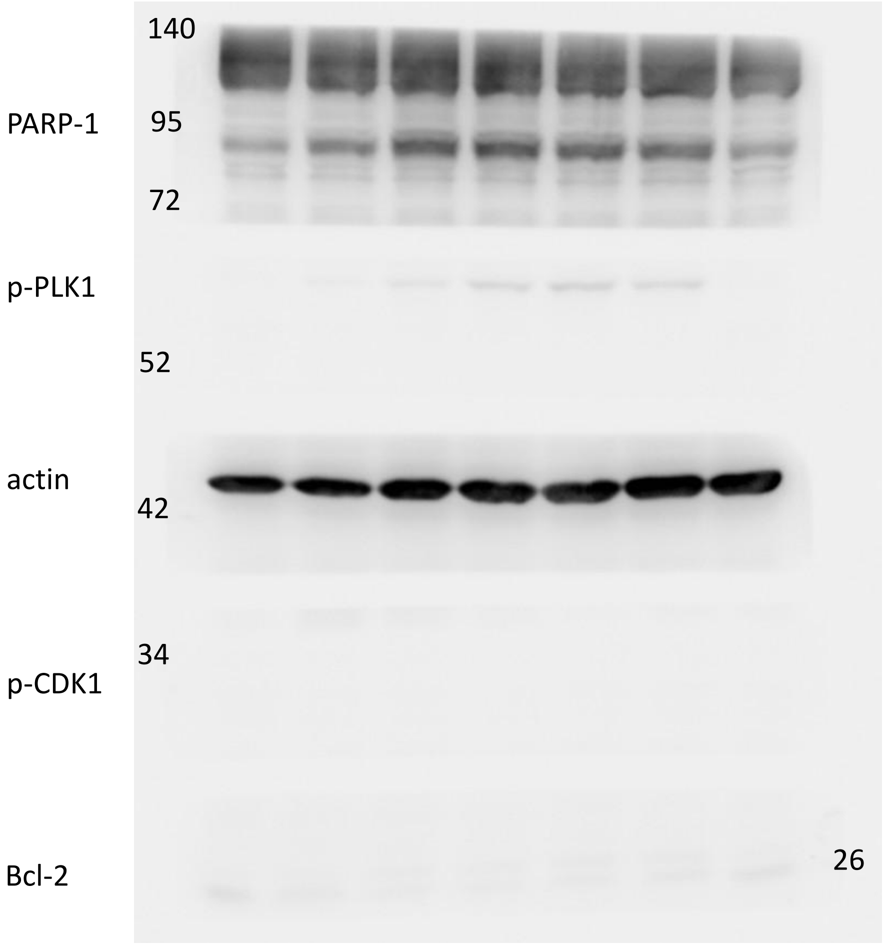

exposure 10s

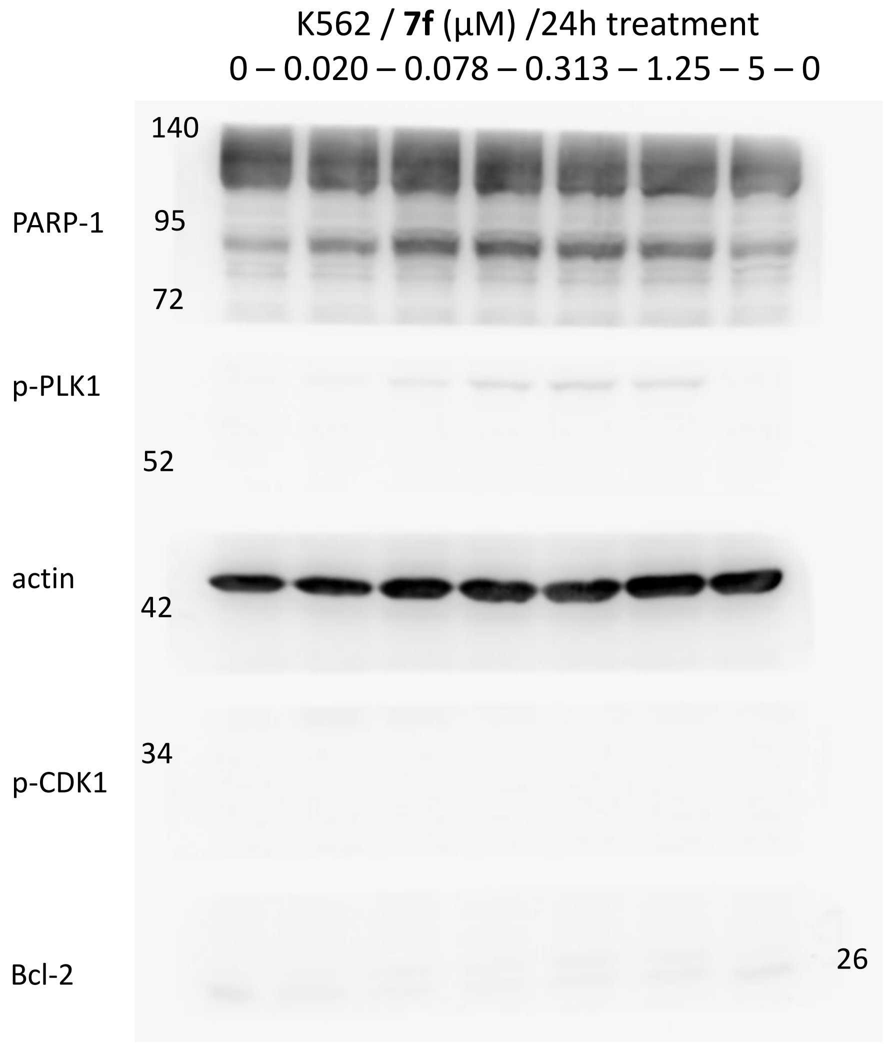

exposure 20s

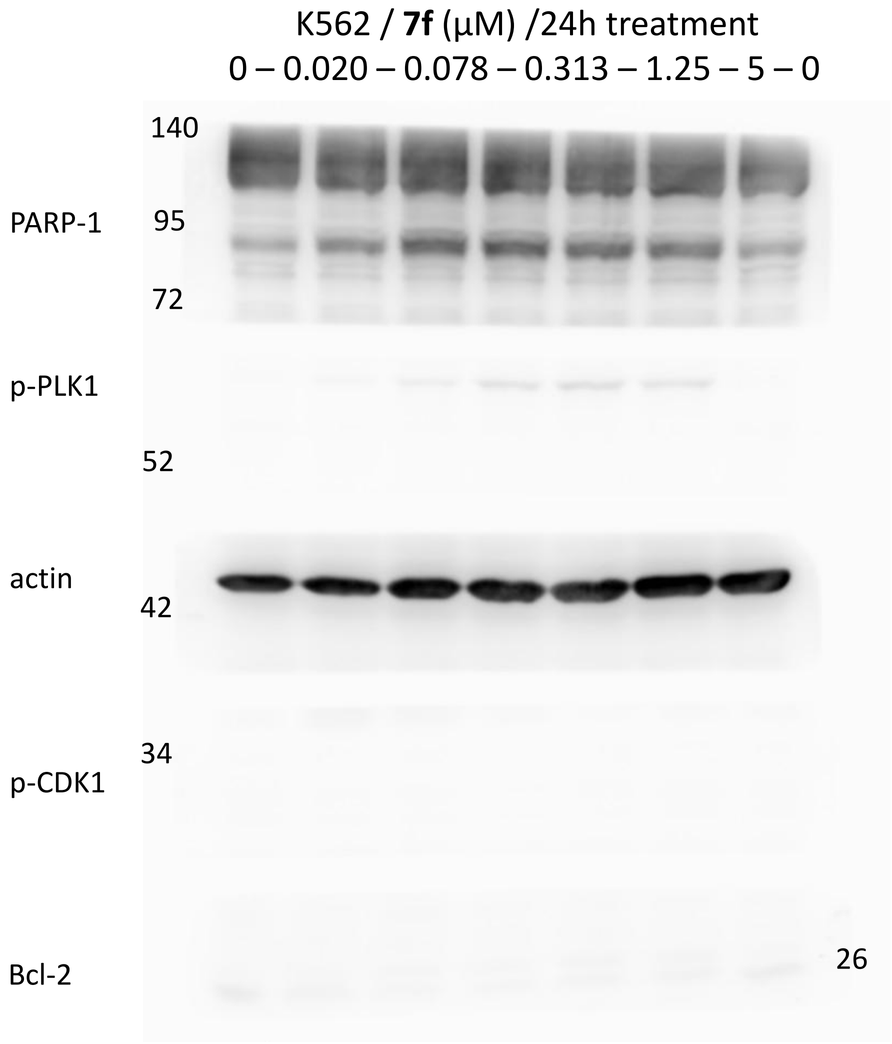

exposure 40s

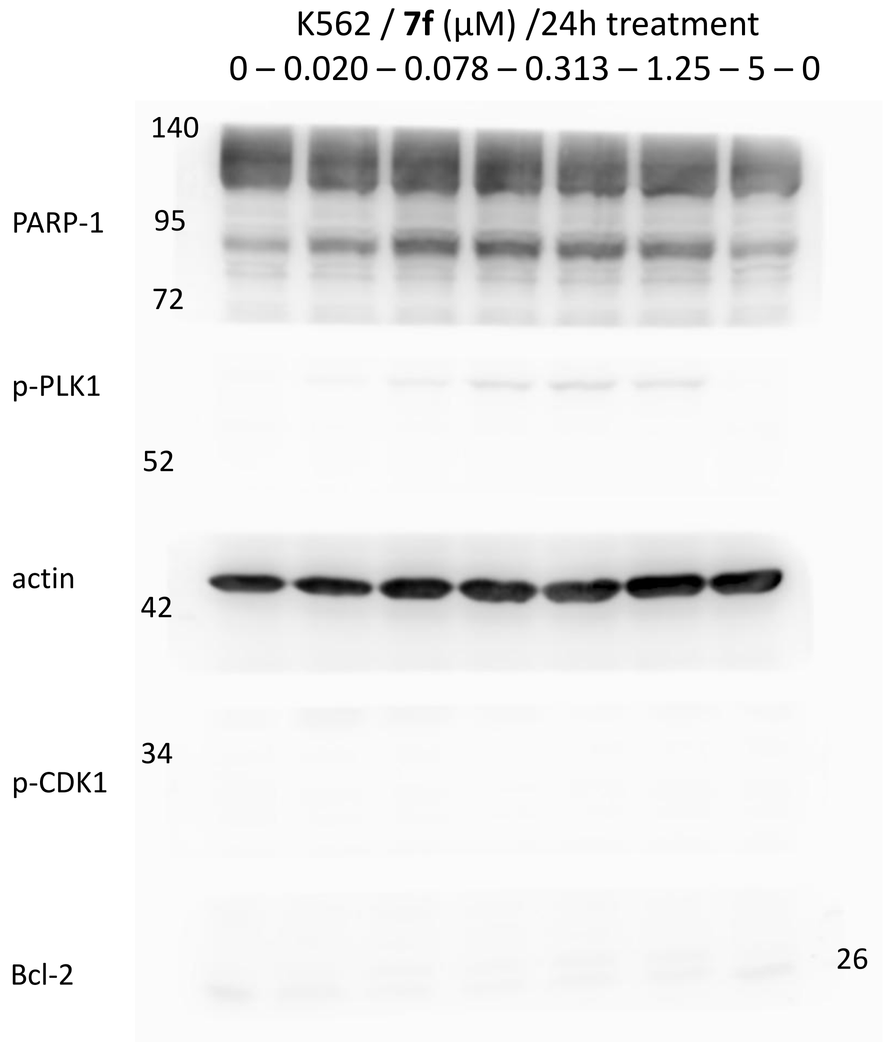

exposure 80s

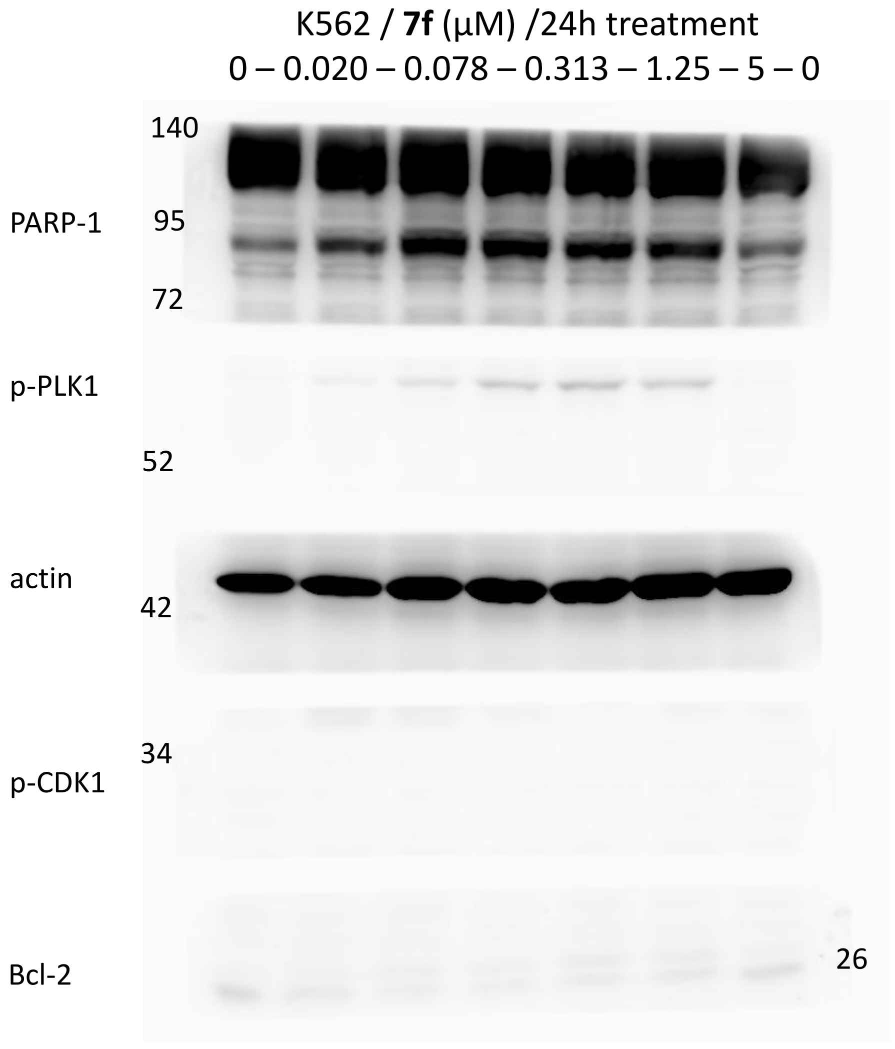

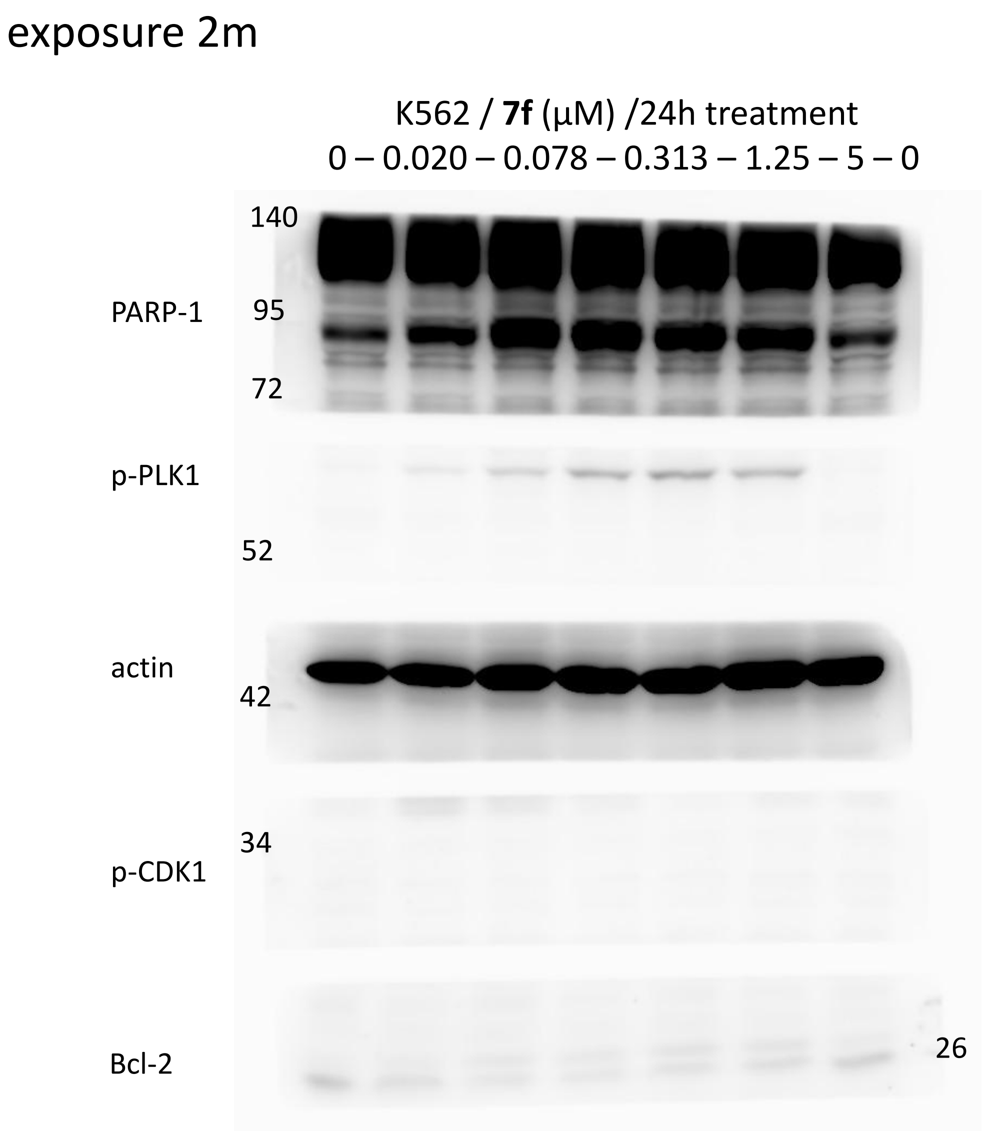
exposure 2m
K562 / **7f** (µM) /24h treatment
0 – 0.020 – 0.078 – 0.313 – 1.25 – 5 – 0
140
PARP-1
95
72
p-PLK1
52
actin
42
34
p-CDK1
Bcl-2
26

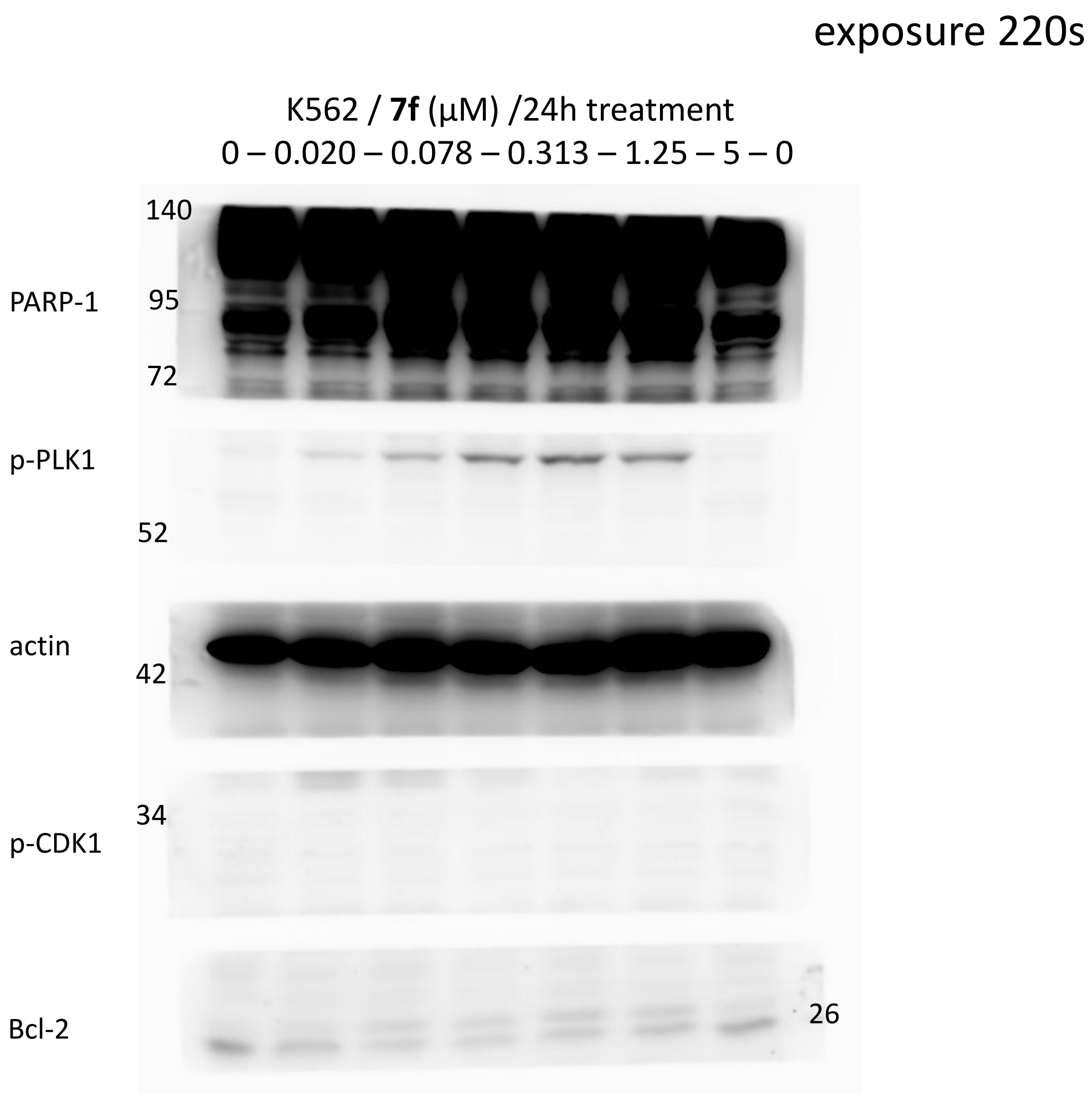
exposure 220s
K562 / **7f** (µM) /24h treatment
0 – 0.020 – 0.078 – 0.313 – 1.25 – 5 – 0
140
PARP-1
95
72
p-PLK1
52
actin
42
34
p-CDK1
Bcl-2
26

exposure 5m

exposure 10m

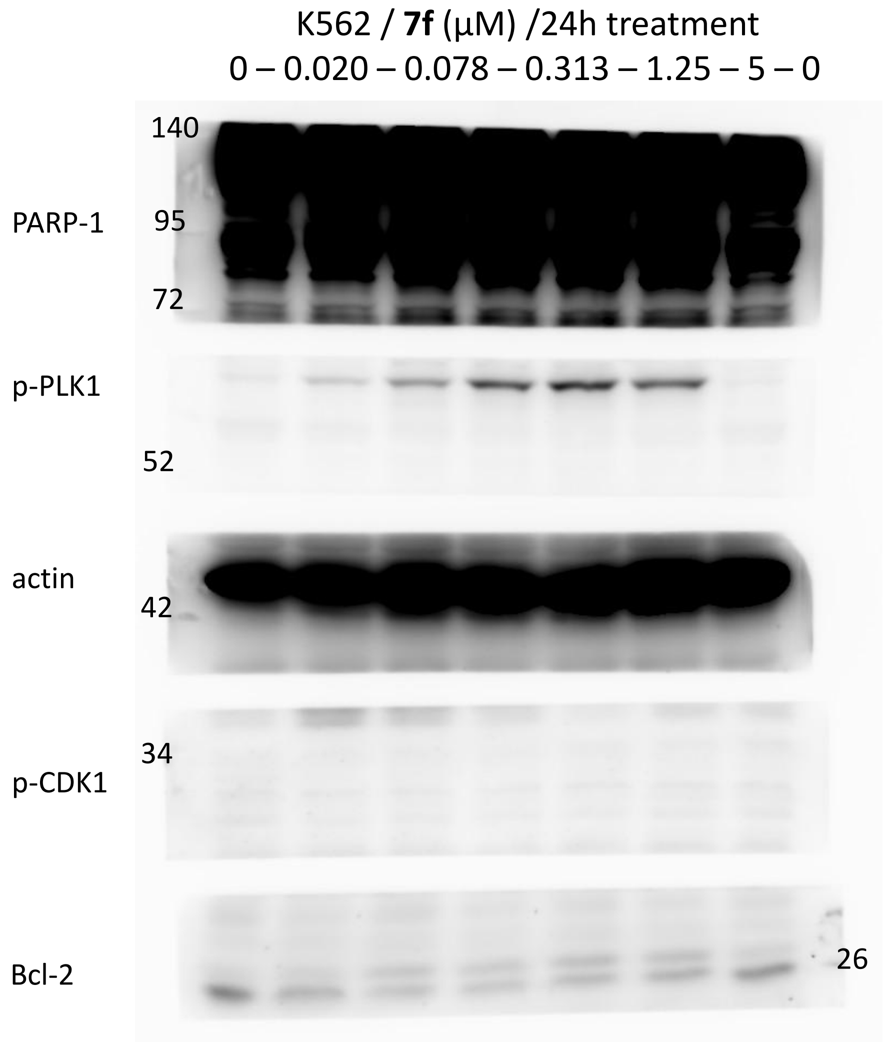

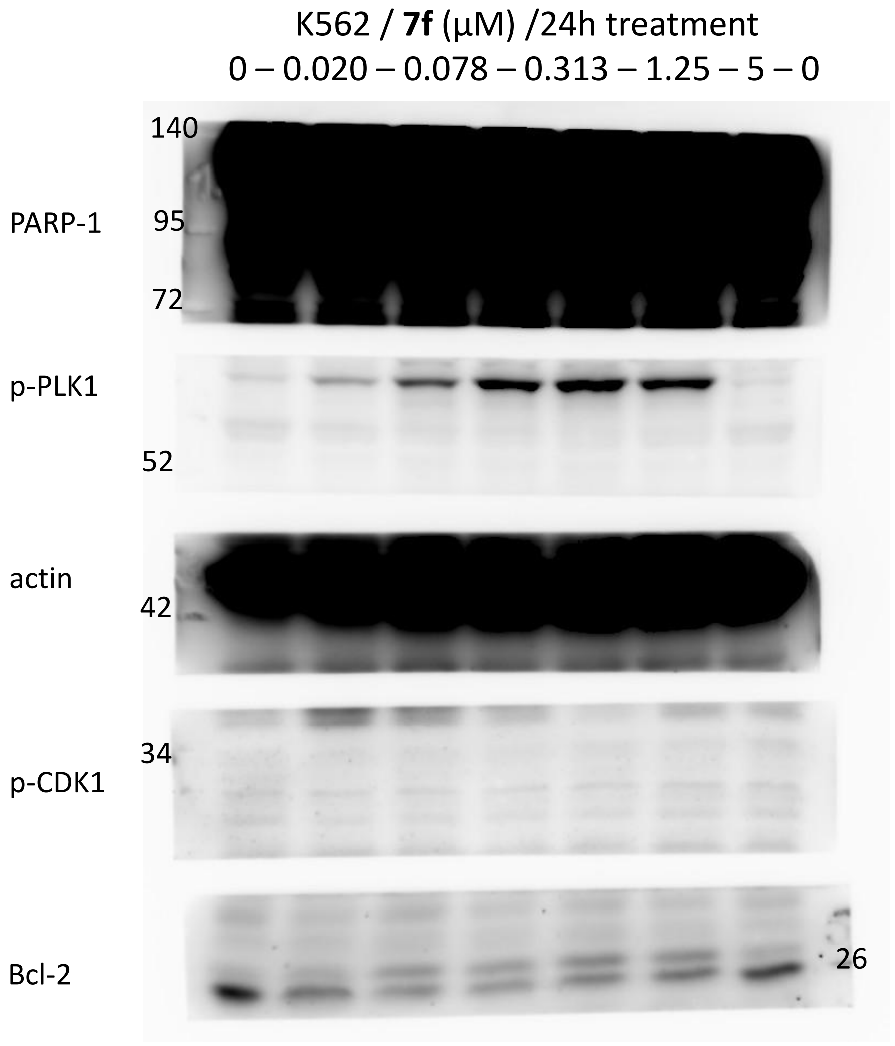

K562 / **7f** (µM) /24h treatment K562 / **7f** (µM) /24h treatment

0 – 0.020 – 0.078 – 0.313 – 1.25 – 5 – 0 0 – 0.020 – 0.078 – 0.313 – 1.25 – 5 – 0

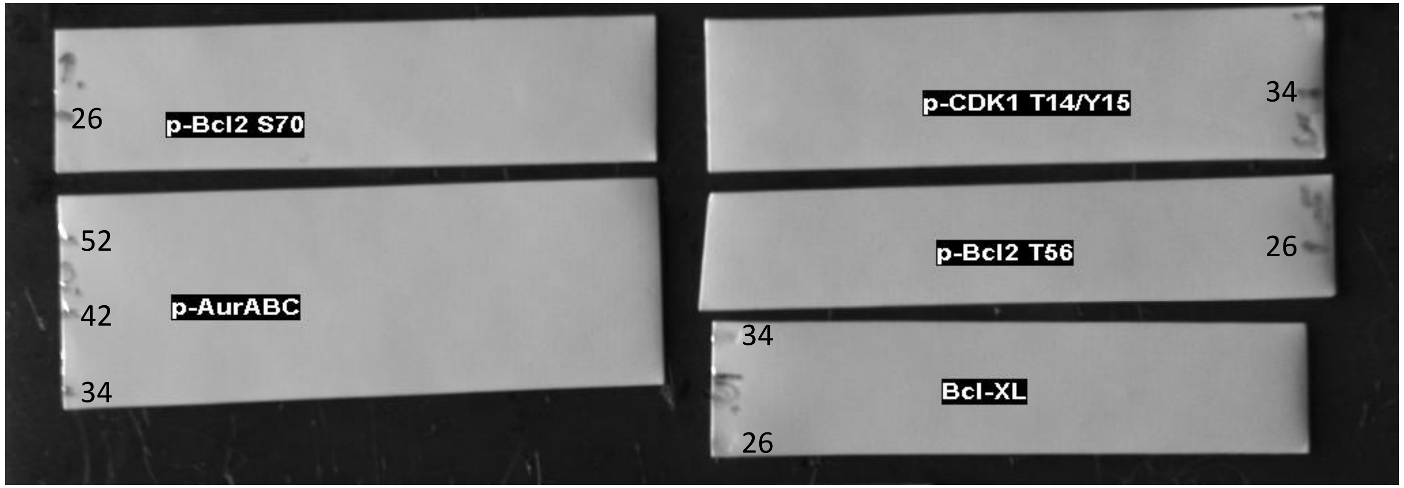

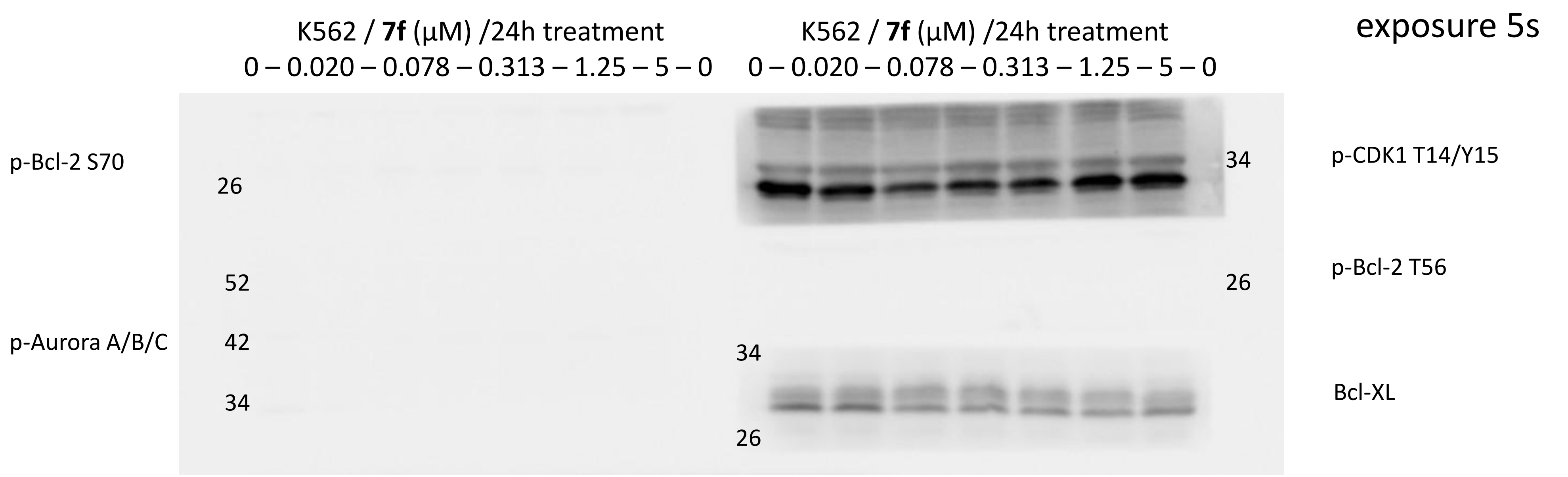
K562 / 7f (μM) /24h treatment
0 – 0.020 – 0.078 – 0.313 – 1.25 – 5 – 0
K562 / 7f (μM) /24h treatment
0 – 0.020 – 0.078 – 0.313 – 1.25 – 5 – 0
exposure 5s
p-Bcl-2 S70
26
52
p-Aurora A/B/C
42
34
34
p-CDK1 T14/Y15
p-Bcl-2 T56
26
34
Bcl-XL
26

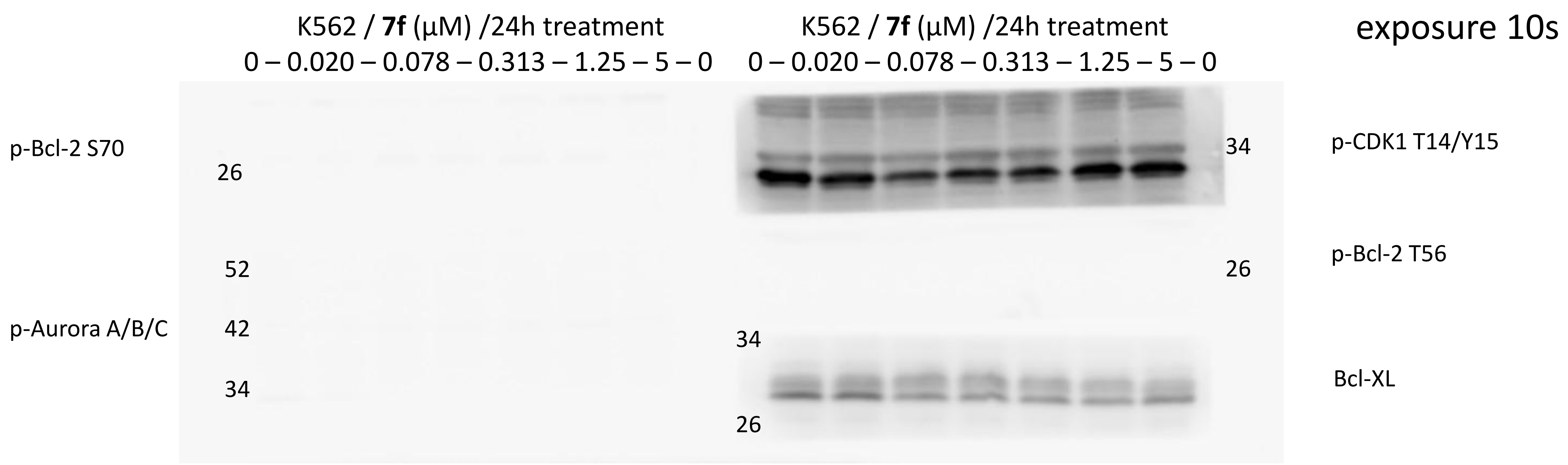
K562 / 7f (μM) /24h treatment
0 – 0.020 – 0.078 – 0.313 – 1.25 – 5 – 0
K562 / 7f (μM) /24h treatment
0 – 0.020 – 0.078 – 0.313 – 1.25 – 5 – 0
exposure 10s
p-Bcl-2 S70
26
52
p-Aurora A/B/C
42
34
34
p-CDK1 T14/Y15
p-Bcl-2 T56
26
34
Bcl-XL
26

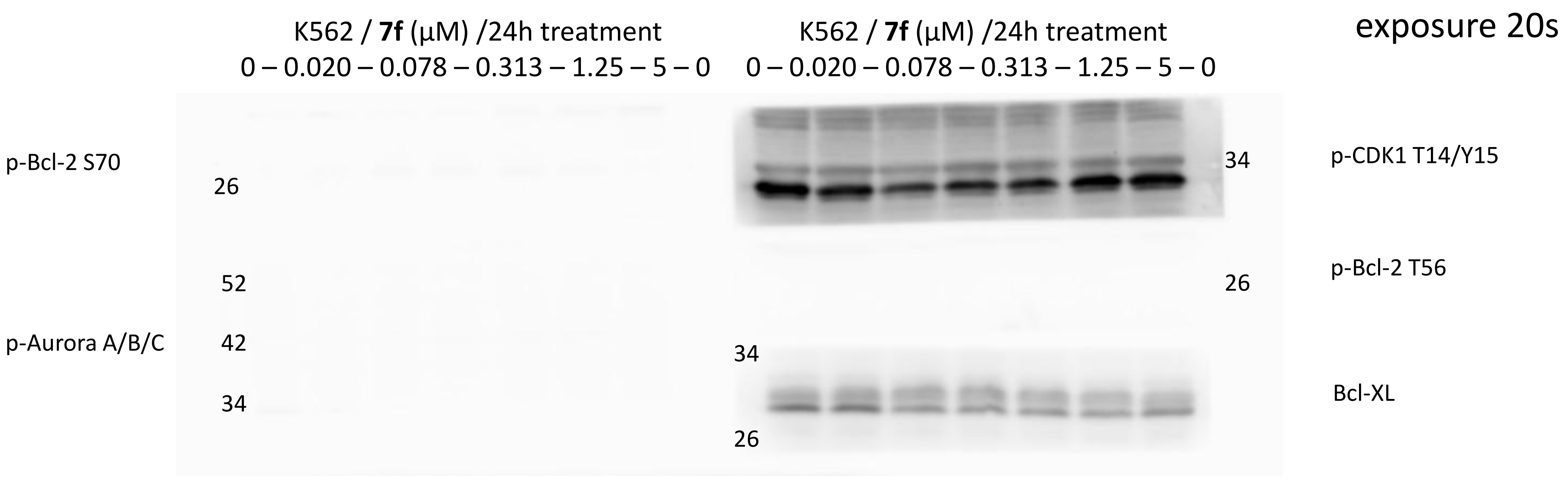
exposure 20s
K562 / 7f (µM) /24h treatment
0 – 0.020 – 0.078 – 0.313 – 1.25 – 5 – 0
K562 / 7f (µM) /24h treatment
0 – 0.020 – 0.078 – 0.313 – 1.25 – 5 – 0
p-Bcl-2 S70
26
52
p-Aurora A/B/C
42
34
34
26
34
26
p-CDK1 T14/Y15
p-Bcl-2 T56
Bcl-XL

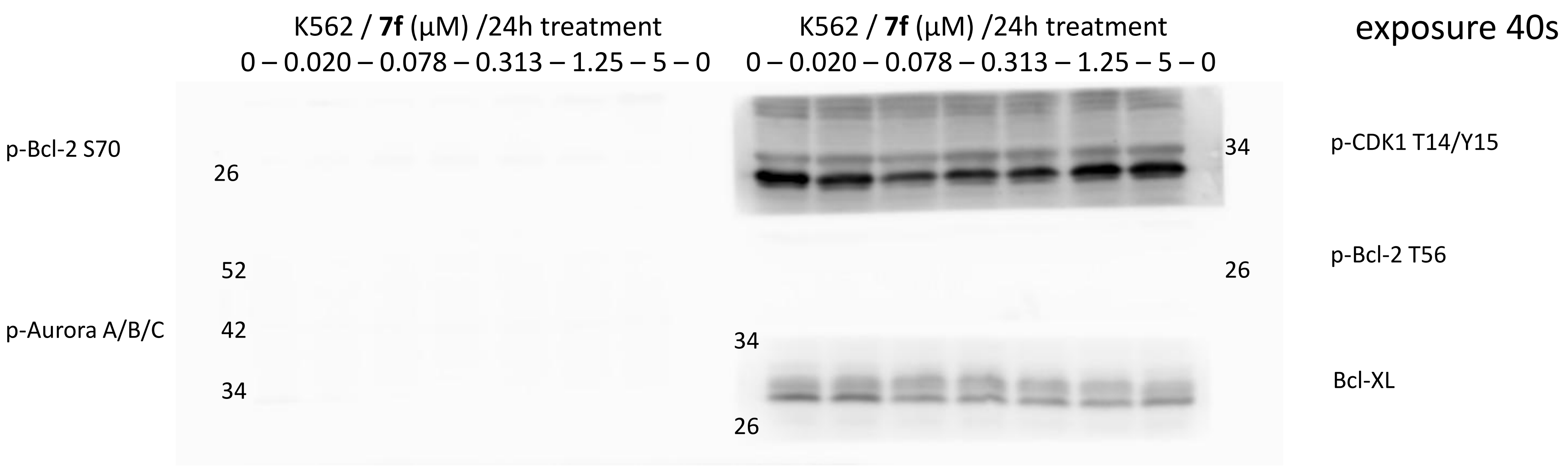
exposure 40s
K562 / 7f (µM) /24h treatment
0 – 0.020 – 0.078 – 0.313 – 1.25 – 5 – 0
K562 / 7f (µM) /24h treatment
0 – 0.020 – 0.078 – 0.313 – 1.25 – 5 – 0
p-Bcl-2 S70
26
52
p-Aurora A/B/C
42
34
34
26
34
26
p-CDK1 T14/Y15
p-Bcl-2 T56
Bcl-XL

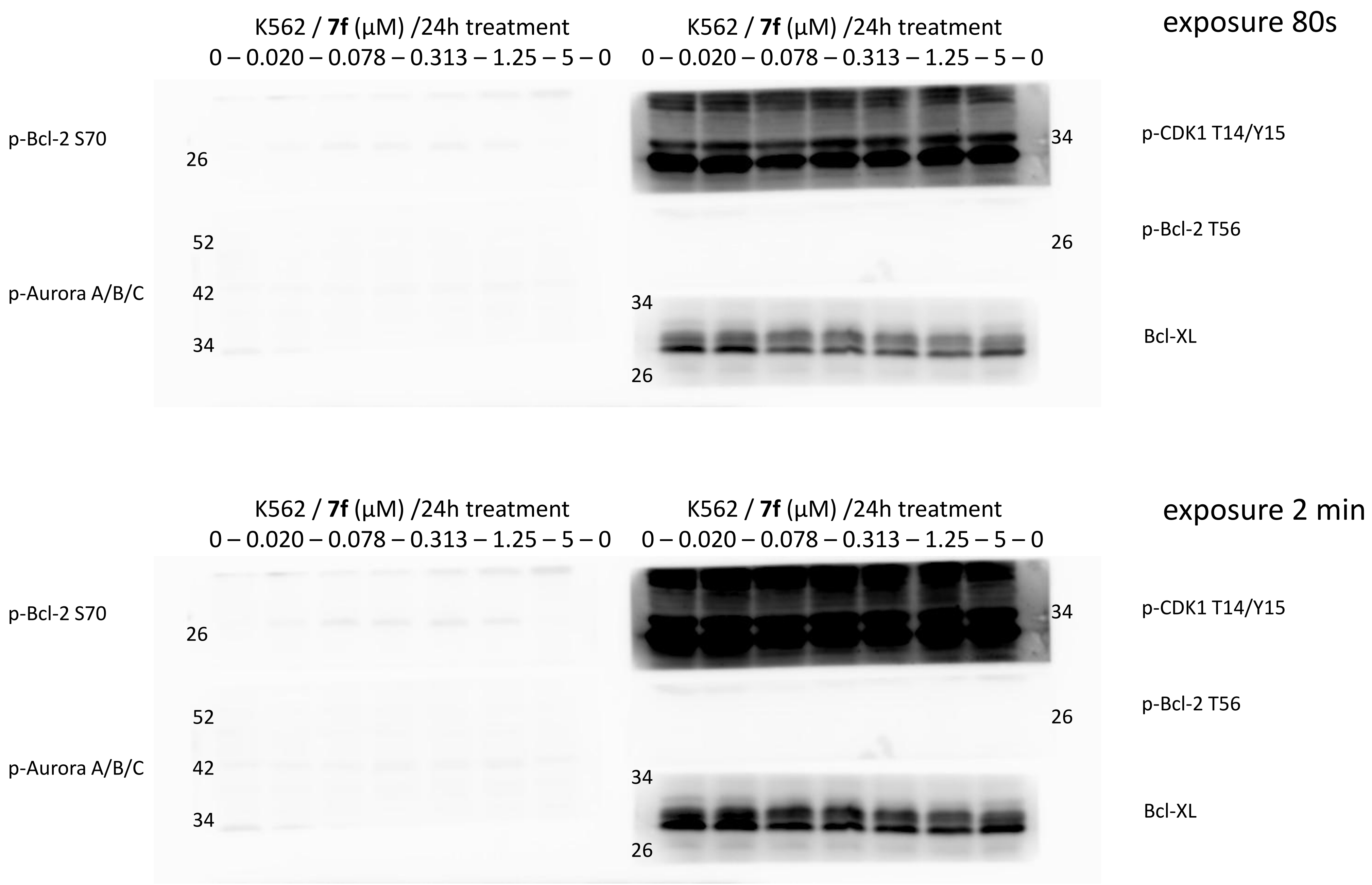
K562 / 7f (μM) /24h treatment
0 – 0.020 – 0.078 – 0.313 – 1.25 – 5 – 0
K562 / 7f (μM) /24h treatment
0 – 0.020 – 0.078 – 0.313 – 1.25 – 5 – 0
exposure 80s
p-Bcl-2 S70
26
p-CDK1 T14/Y15
34
p-Bcl-2 T56
26
52
p-Aurora A/B/C
42
34
34
Bcl-XL
26
K562 / 7f (μM) /24h treatment
0 – 0.020 – 0.078 – 0.313 – 1.25 – 5 – 0
K562 / 7f (μM) /24h treatment
0 – 0.020 – 0.078 – 0.313 – 1.25 – 5 – 0
exposure 2 min
p-Bcl-2 S70
26
p-CDK1 T14/Y15
34
p-Bcl-2 T56
26
52
p-Aurora A/B/C
42
34
34
Bcl-XL
26

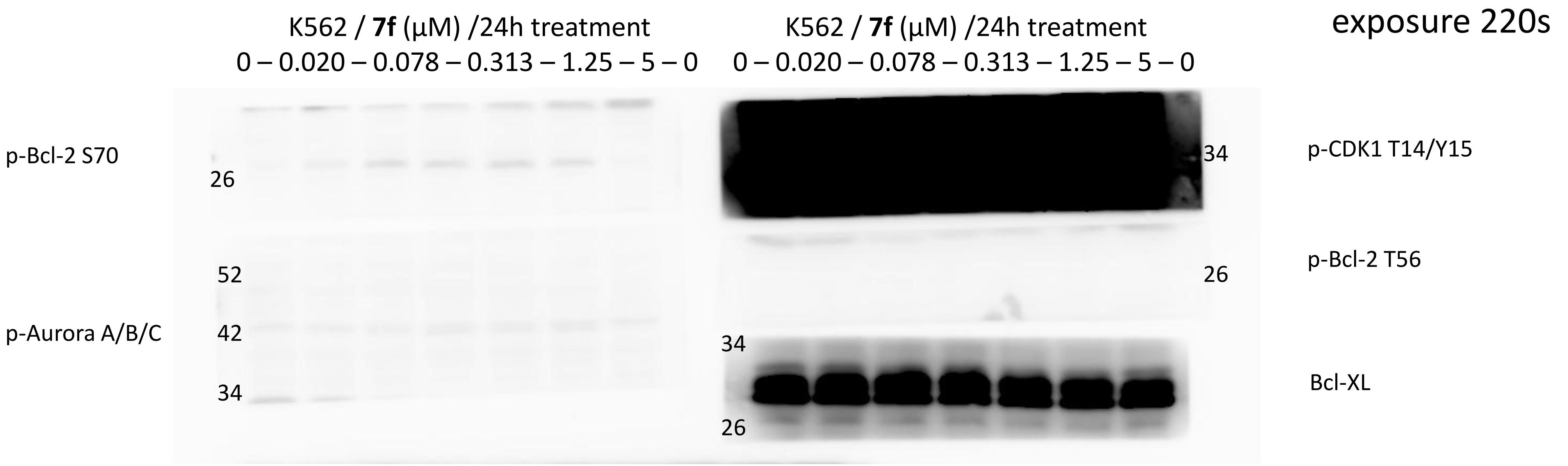
exposure 220s
K562 / 7f (µM) /24h treatment
0 – 0.020 – 0.078 – 0.313 – 1.25 – 5 – 0
K562 / 7f (µM) /24h treatment
0 – 0.020 – 0.078 – 0.313 – 1.25 – 5 – 0
p-Bcl-2 S70
26
52
p-Aurora A/B/C
42
34
34
p-CDK1 T14/Y15
p-Bcl-2 T56
26
34
Bcl-XL
26

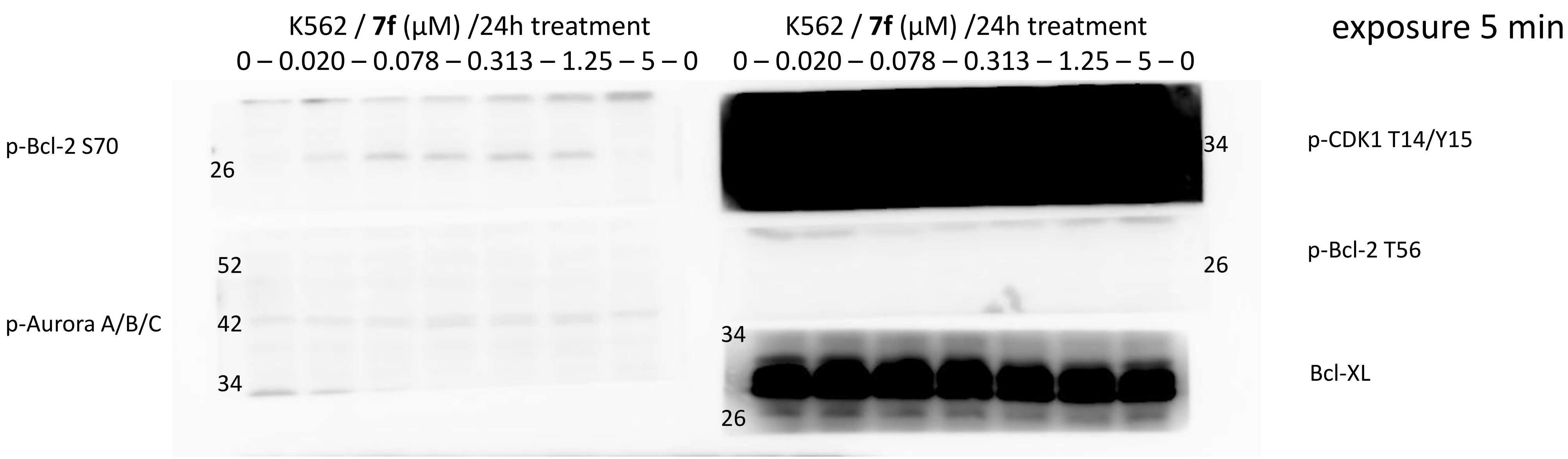
exposure 5 min
K562 / 7f (µM) /24h treatment
0 – 0.020 – 0.078 – 0.313 – 1.25 – 5 – 0
K562 / 7f (µM) /24h treatment
0 – 0.020 – 0.078 – 0.313 – 1.25 – 5 – 0
p-Bcl-2 S70
26
52
p-Aurora A/B/C
42
34
34
p-CDK1 T14/Y15
p-Bcl-2 T56
26
34
Bcl-XL
26

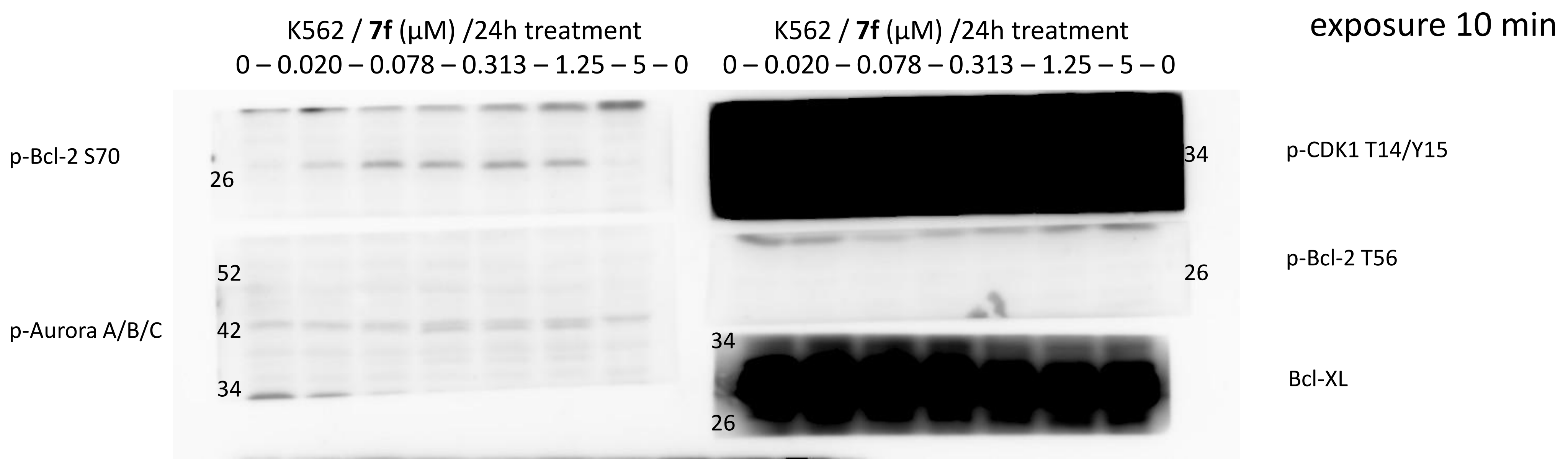
exposure 10 min
K562 / 7f (µM) /24h treatment
0 – 0.020 – 0.078 – 0.313 – 1.25 – 5 – 0
K562 / 7f (µM) /24h treatment
0 – 0.020 – 0.078 – 0.313 – 1.25 – 5 – 0
p-Bcl-2 S70
26
p-Aurora A/B/C
52
42
34
34
p-CDK1 T14/Y15
26
p-Bcl-2 T56
34
26
Bcl-XL

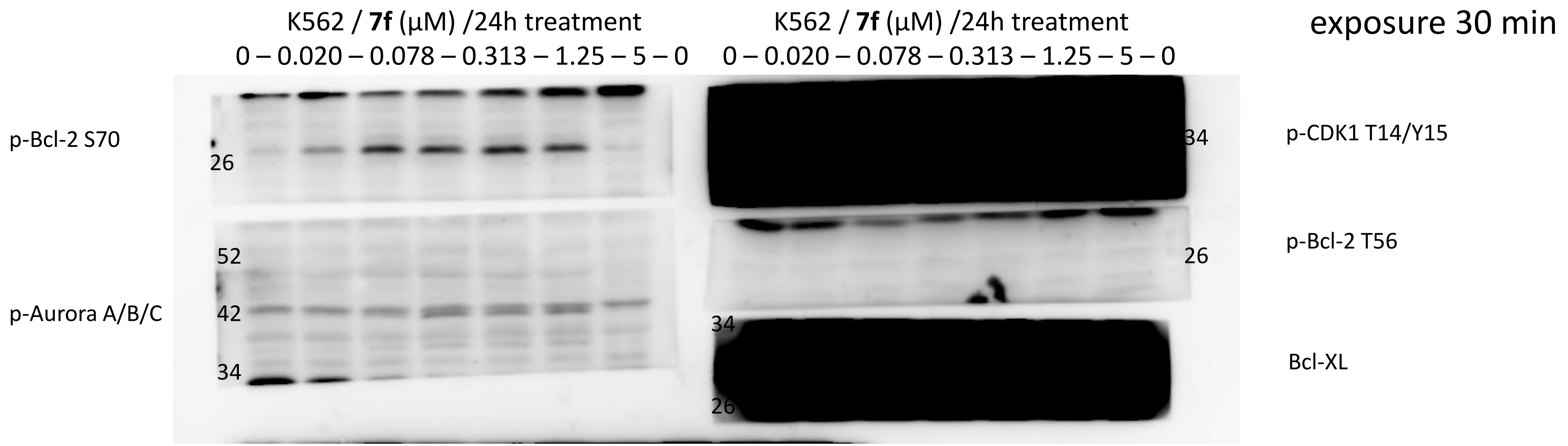
exposure 30 min
K562 / 7f (µM) /24h treatment
0 – 0.020 – 0.078 – 0.313 – 1.25 – 5 – 0
K562 / 7f (µM) /24h treatment
0 – 0.020 – 0.078 – 0.313 – 1.25 – 5 – 0
p-Bcl-2 S70
26
p-Aurora A/B/C
52
42
34
34
p-CDK1 T14/Y15
26
p-Bcl-2 T56
34
26
Bcl-XL
